# Evolutionary morphology of sperm in pholcid spiders (Pholcidae, Synspermiata)

**DOI:** 10.1186/s40850-022-00148-3

**Published:** 2022-09-26

**Authors:** Tim M. Dederichs, Bernhard A. Huber, Peter Michalik

**Affiliations:** 1 Zoologisches Institut und Museum, Universität Greifswald, Loitzer Straße 26, Greifswald, 17489 Germany; 2 Zoologisches Forschungsmuseum Alexander Koenig - Leibniz-Institut für Biodiversität der Tiere (LIB), Adenaueralle 127, Bonn, 53113 Germany

**Keywords:** Arachnida, Male genitalia, Sperm conjugation, Sperm evolution, Sperm transfer form, Spiders

## Abstract

**Background:**

Pholcidae represent one of the largest and most diverse spider families and have been subject to various studies regarding behavior and reproductive biology. In contrast to the solid knowledge on phylogeny and general reproductive morphology, the primary male reproductive system is strongly understudied, as it has been addressed only for few species. Those studies however suggested a high diversity of sperm and seminal secretions across the family. To address this disparity and reconstruct the evolution of sperm traits, we investigate the primary male reproductive system of pholcid spiders by means of light, X-ray, and transmission electron microscopy using a comprehensive taxon sampling with 46 species from 33 genera, representing all five subfamilies.

**Results:**

Our data show a high disparity of sperm morphology and seminal secretions within pholcids. We document several sperm characters that are unique for pholcids, such as a helical band (Pholcinae) or a lamellate posterior centriolar adjunct material (Modisiminae). Character mapping revealed several putative synapomorphies for individual taxa. With regard to sperm transfer forms, we found that synspermia occur only in the subfamily Ninetinae, whereas the other subfamilies have cleistospermia. In several species with cleistospermia, we demonstrate that spermatids remain fused until late stages of spermiogenesis before ultimately separating shortly before the coiling process. Additionally, we explored the previously hypothesized correlation between sperm size and minimum diameter of the spermophor in the male palpal organ. We show that synspermia differ strongly in size whereas cleistospermia are rather uniform, but neither transfer form is positively correlated with the diameter of the spermophor.

**Conclusions:**

Our data revealed a dynamic evolution of sperm characters, with convergences across all subfamilies and a high level of homoplasy. The present diversity can be related to subfamily level and allows for assignments of specific subtypes of spermatozoa. Our observations support the idea that Ninetinae are an ancestral clade within Pholcidae that have retained synspermia and that synspermia represent the ancestral sperm transfer form of Pholcidae.

**Supplementary Information:**

The online version contains supplementary material available at 10.1186/s40850-022-00148-3.

## Background

Daddy long-legs spiders (Pholcidae) are among the spiders best-known to the general public as some species, such as the long-bodied cellar spider *Pholcus phalangioides* (Fuesslin, 1775), have a synanthropic lifestyle. The family currently counts more than 1,800 described species in over 90 genera [1] representing one of the most species-rich spider families. Pholcid spiders are morphologically and ecologically highly diverse (e.g. [2, 3]) and phylogenetic relationships have been addressed in numerous morphological and molecular studies hypothesizing five subfamilies—Pholcinae (922 spp.), Smeringopinae (125 spp.), Modisiminae (480 spp.), Arteminae (99 spp.) and Ninetinae (34 spp.) (e.g. [3–7]).

Pholcid systematics has been extensively studied during the last decades and 68% of the known species have been described since the year 2000 (www.pholcidae.de). The biology of the group is much less well known but apparently equally diverse. For example, pholcid spiders show an interesting reproductive biology with different reproductive strategies, many sexual dimorphisms, and a high disparity of genitalic structures including asymmetry, genital polymorphism, and fundamentally different configurations of the female internal genitalia (e.g. [2, 8–15]). In contrast to the vast knowledge on the gross morphology of female and male genitalia, the primary male reproductive system is severely understudied. Only three species have been investigated in detail: *Pholcus phalangioides* (Pholcinae) [16–18], *Holocnemus pluchei* (Scopoli, 1763) (Smeringopinae) [19, 20] and *Psilochorus simoni* (Berland, 1911) (Modisiminae) [21]. Moreover, Michalik and Ramírez [22] included the ninetine *Gertschiola macrostyla* (Mello-Leitão, 1941) in their comprehensive study on spider spermatozoa, but did not describe the sperm morphology in detail. These previous studies suggested a remarkable diversity of sperm structures, raising questions about the selective forces driving sperm evolution. At the same time, this morphological diversity is potentially informative in phylogeny reconstruction. For example, the investigation of sperm structures across the spider tree of life by Michalik and Ramírez [22] recovered synapomorphies for a wide range of taxa, such as synspermia as the characteristic of the Synspermiata – a clade of haplogyne spiders that includes Pholcidae and that was also recovered by all consecutive phylogenomic studies [23–25].

Male spiders transfer sperm with their modified pedipalps, which can be complex or simple depending on the group [26]. The sperm cells are transferred in a coiled state, in so-called transfer forms [27]. Based on the influential studies of Gerd Alberti, three major types of transfer forms can be distinguished within spiders—coenospermia (aggregations of multiple individual sperm cells with a common secretory sheath), synspermia (aggregations of several fused sperm cells forming a syncytium) and cleistospermia (single individual sperm cells, each with its own secretory sheath) [16, 27, 28]. For pholcid spiders, previous studies had suggested that cleistospermia are the common transfer form in this family, but the study by Michalik and Ramirez [22] briefly reported the presence of synspermia in the ninetine *Gertschiola macrostyla*. This finding is of particular interest as the subfamily Ninetinae has long been grouped at the same taxonomic level as all other Pholcidae together [29] or as the possible sister-group to the remaining subfamilies [7]. This suggests that synspermia is the plesiomorphic transfer form within this family. Moreover, the presence of two different types of transfer forms is also of interest with regard to the evolution of male genitalia. Since synspermia are usually much larger than cleistospermia [22], a positive correlation between the minimum spermophor diameter and the dimension of a single sperm transfer unit can be hypothesized (see also [30–32]). Lipke, Ramírez and Michalik [33] addressed this issue in Orsolobidae, but could not find a positive correlation, possibly because of the low sample size. However, a co-evolution between sperm length and genitalia has been reported for insects (e.g. [34]).

Sexual selection not only acts on the evolution of spermatozoa [35], but has also resulted in a diversity of seminal products that are transferred into the female sperm storage organs together with the sperm cells. It is known from insects that seminal proteins are strategically allocated in the female genital tract as a response to potential sperm competition (e.g. [36–39]). In contrast to insects, spider males do not have accessory glands, but produce seminal secretions directly in the testes and deferent ducts. The seminal fluid of spiders bears a huge interspecific diversity and can contain a variety of structurally different secretory droplets [22, 40]. It was hypothesized that diversification of such seminal secretions may be driven by postcopulatory sexual selection (e.g. [41–43]). One spider group with a particularly high degree of seminal secretion diversity seems to be Pholcidae. The three previously investigated species had very different secretions not only structurally but also in the number of different types of secretory droplets [21].

In this study, we address the evolutionary morphology of the primary male reproductive system including spermiogenesis, sperm cells and sperm transfer forms of pholcid spiders using light, transmission, and X-ray microscopy. With 46 species from 33 genera and all five subfamilies, our taxon sampling is the most comprehensive for such an intrafamiliar study in spiders to date. Thus, we provide one of the most detailed insights into the evolution of sperm within a species-rich spider family. Our analysis builds on the framework for spider sperm morphology proposed by Michalik and Ramírez [22]. We reconstructed possible evolutionary scenarios of sperm traits by mapping characters on the phylogeny in Huber, Eberle and Dimitrov [7] [based on the molecular study of [3], one of the most comprehensive molecular phylogenies at family level currently available in arachnology.

## Results

### Primary male reproductive system

**Fig. 1:**
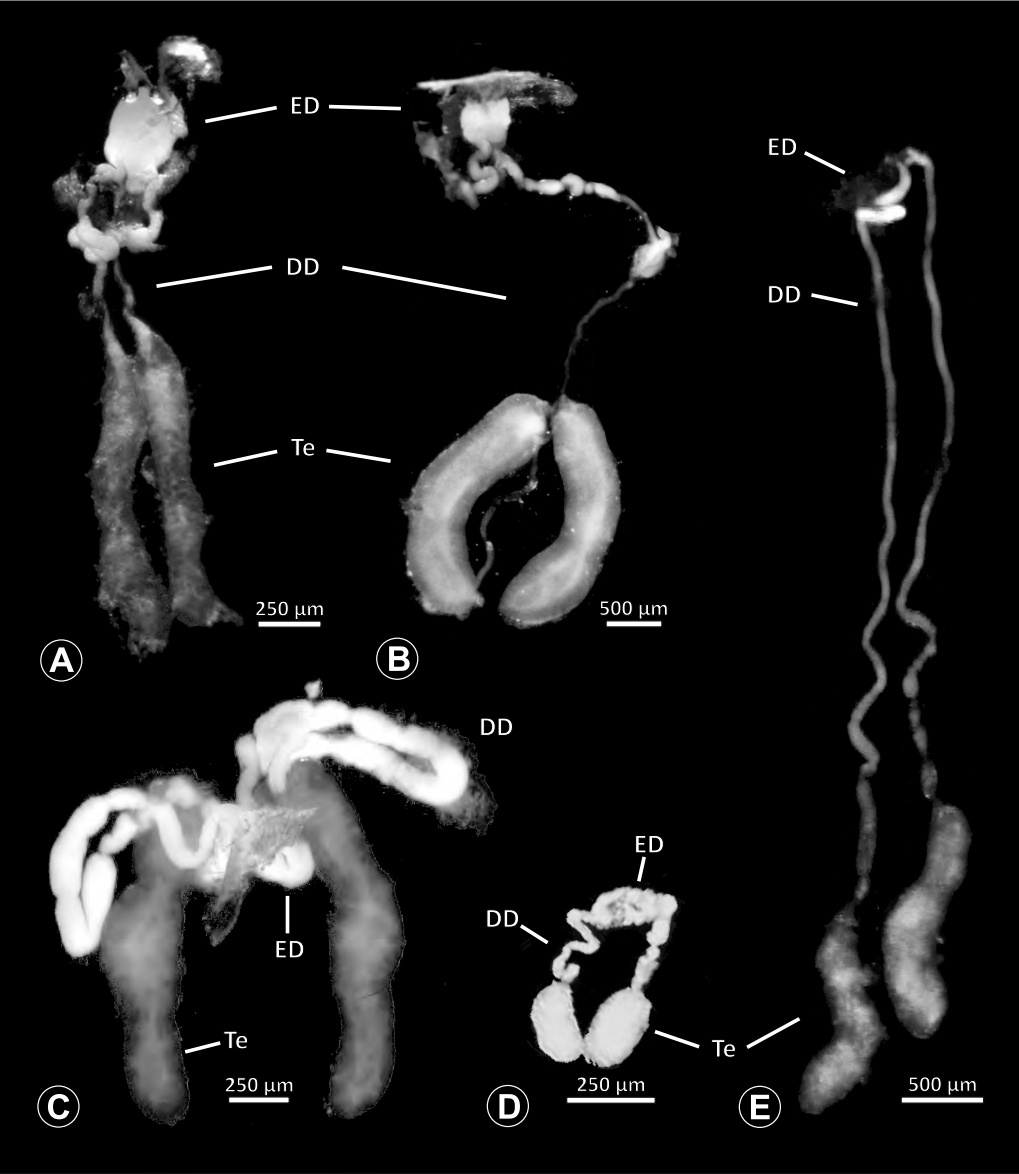
Gross Morphology of the male reproductive system in various pholcid taxa. **A** *Modisimus elongatus*. **B** *Physocyclus globosus*. **C** *Aetana loboc*. **D** *Galapa bella*. **E** *Panjange camiguin*

**Fig. 2:**
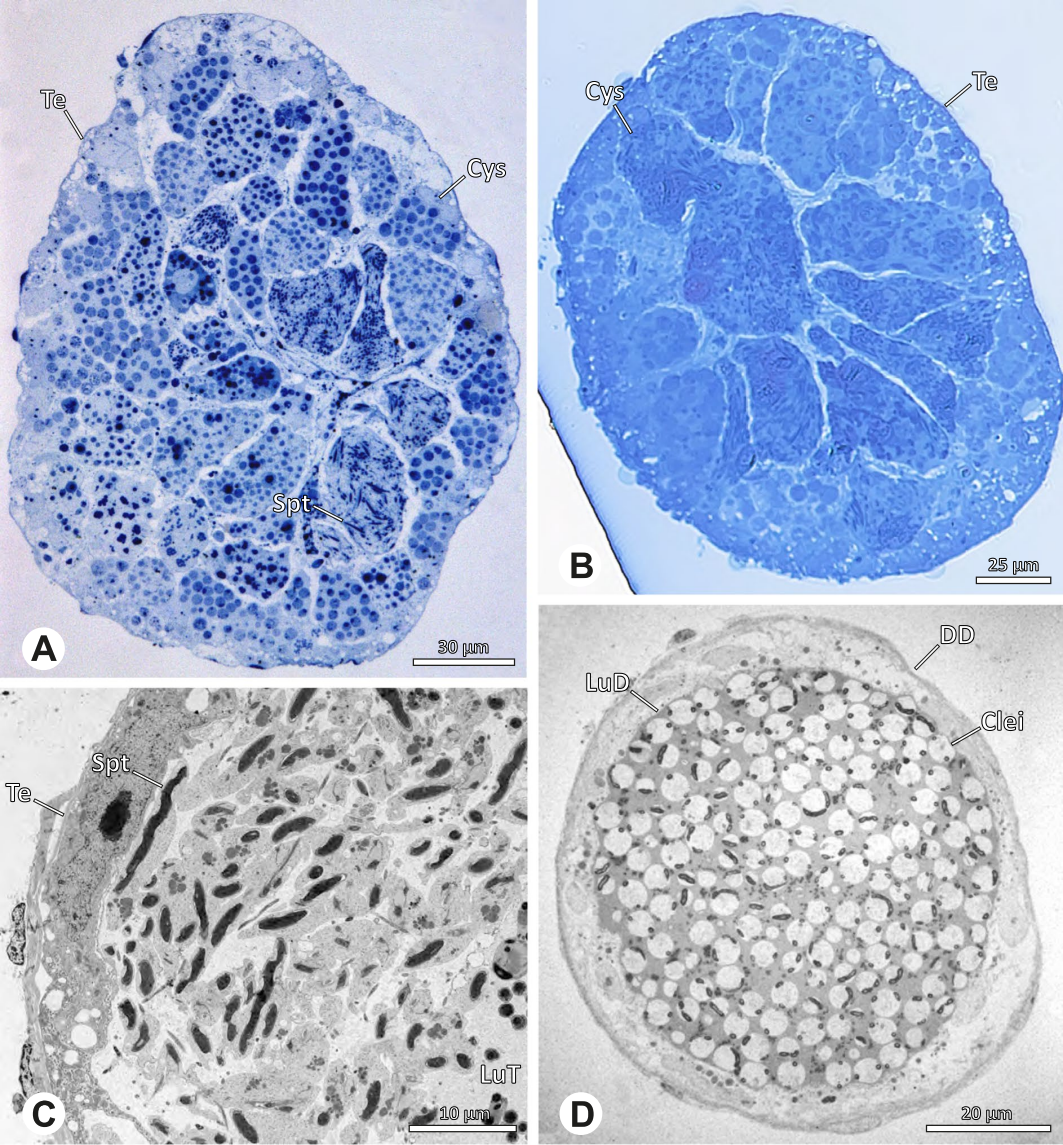
Light- (LM) and transmission electron (TEM) microscopy of testis and deferent duct of different pholcid taxa. **A** *Pholcus bamboutos*, LM, testis. **B** *Belisana* cf. *kinabalu*, LM, testis. **C** *Quamtana oku*, TEM, testis. **D** *Smeringopina bineti*, TEM, deferent duct

**Fig. 3:**
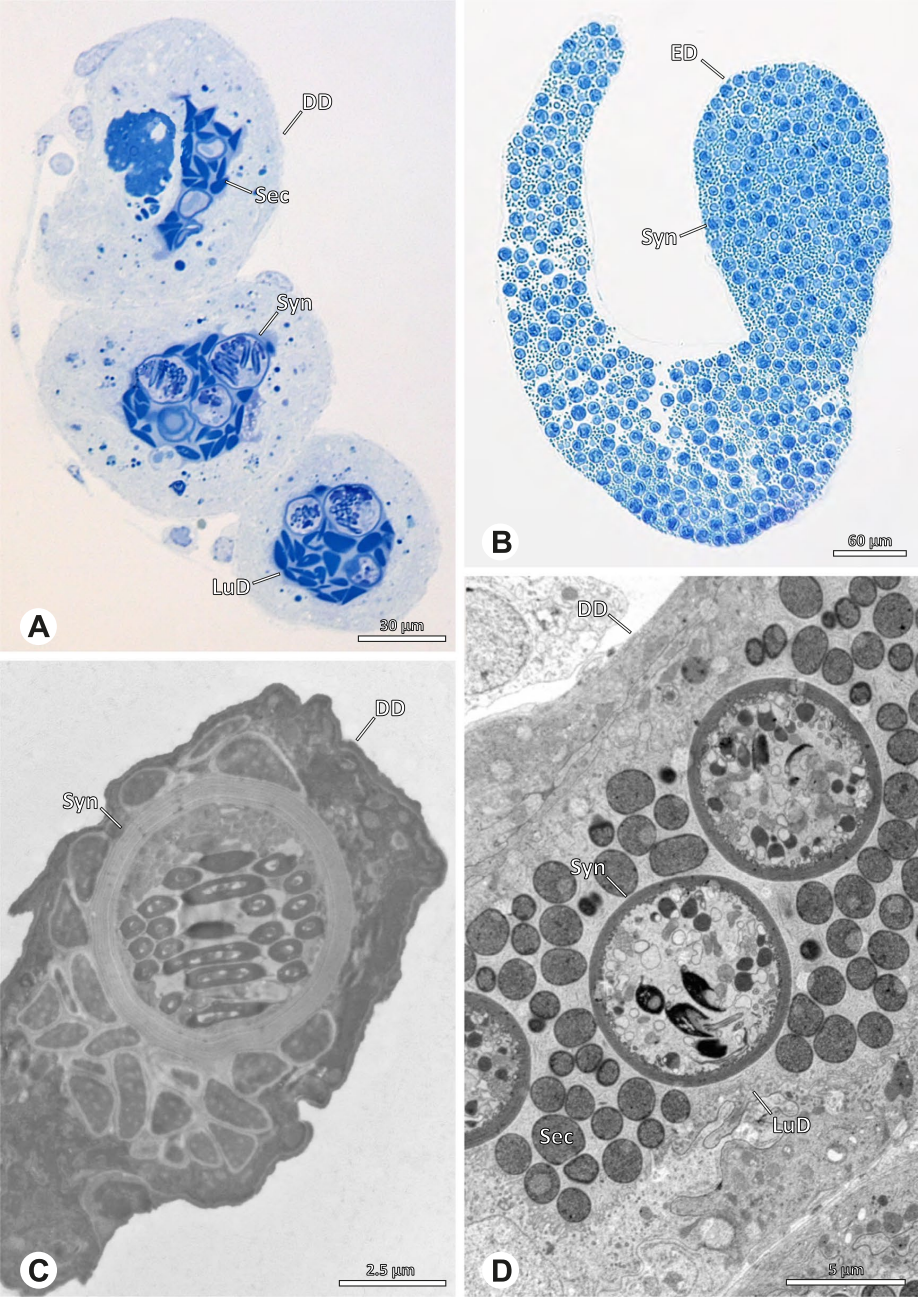
Light (LM) and transmission electron (TEM) microscopy of the deferent duct of different species of the pholcid subfamily Ninetinae. **A** *Gertschiola macrostyla*, LM. **B** *Nerudia sp.* n. ‘Arg58’, LM. **C** *Guaranita goloboffi*, TEM. **D** *Nerudia sp.* n. ‘Mic20’, TEM

**Fig. 4:**
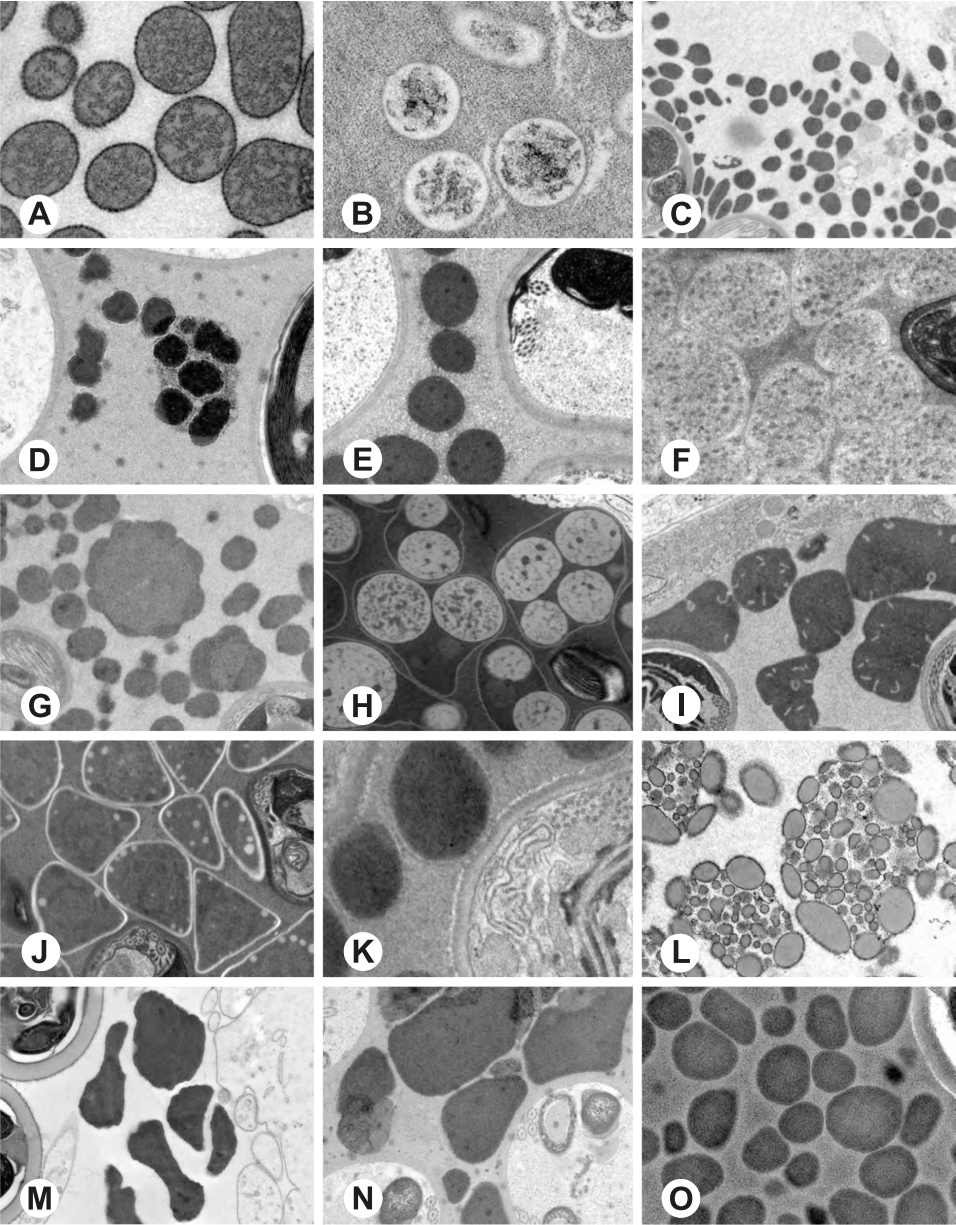
Seminal secretions in the deferent ducts of different pholcid taxa, TEM. **A** *Artema bunkpurugu*. **B** *Physocyclus globosus*. **C** *Smeringopus* cf*. roeweri*. **D** *Smeringopina bineti*. **E** *Smeringopus cylindrogaster*. **F** *Carapoia nairae*. **G** *Chibchea salta*. **H** *Ciboneya antraia*. **I** *Mesabolivar cyaneotaeniatus*. **J** *Modisimus elongatus*. **K** *Tupigea teresopolis*. **L** *Aetana poring*. **M** *Panjange camiguin*. **N** *Leptopholcus guineensis*. **O** *Metagonia* cf. *petropolis*

**Fig. 5:**
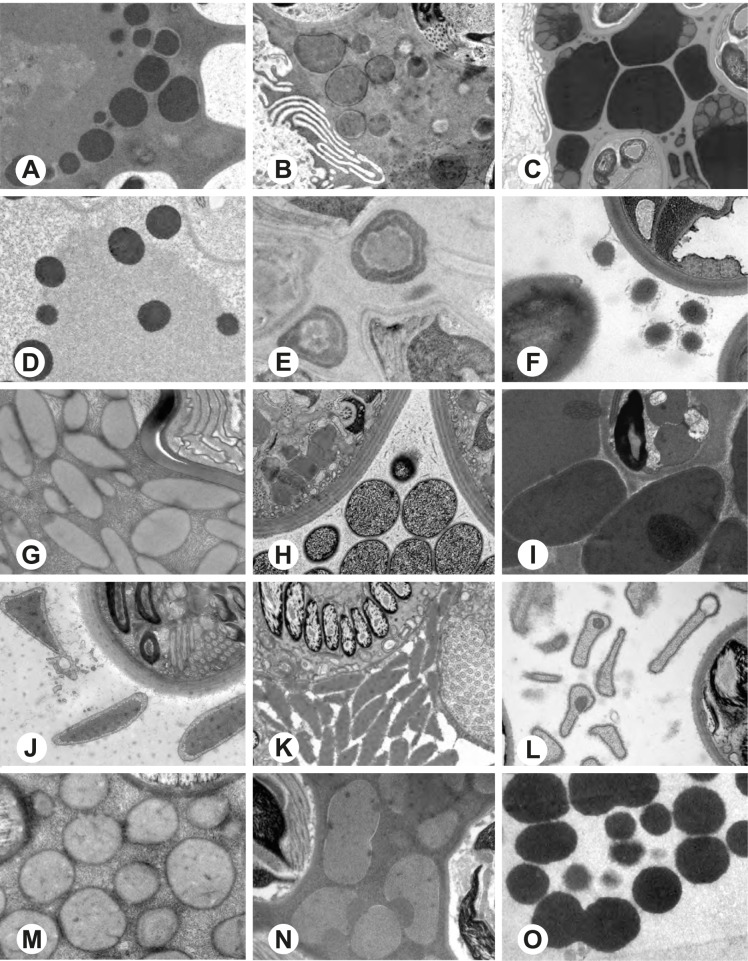
Seminal secretions in the deferent ducts of different pholcid taxa, TEM. **A** *Pehrforsskalia conopyga*. **B** *Pholcus bamboutos*. **C** *Pholcus guineensis*. **D** *Quamtana oku*. **E** *Spermophora awalai*. **F** *Pholcophora* sp. n. ‘Mex22’. **G** *Kambiwa neotropica*. **H** *Nerudia* sp. n. ‘Mic20’. **I** *Gertschiola macrostyla*. **J** *Guaranita goloboffi*. **K** *Galapa bella*. **L** *Tolteca hesperia*. **M** *Canaima?* sp. n. ‘Dup118’ **N** *Mecolaesthus* sp. n. ‘Ecu60’ **O** *Priscula* sp. n. ‘Ecu93’

**Fig. 6:**
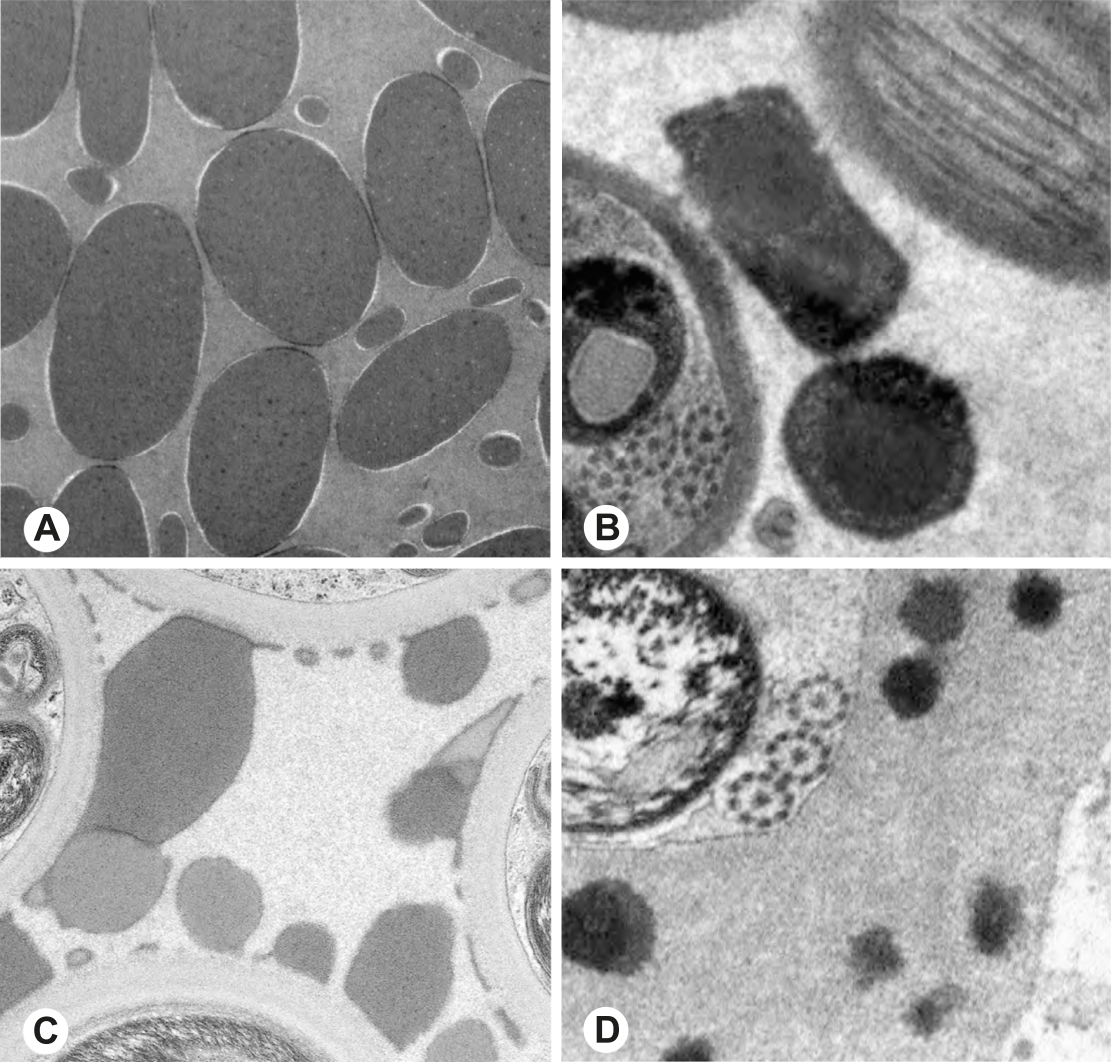
Seminal secretions in the deferent ducts of different pholcid taxa, TEM. **A** *Stygopholcus skotophilus*. **B** *Spermophora senoculata*. **C** *Pholcus opilionoides*. **D** *Cantikus sabah*

The gross morphology of the male reproductive tract in pholcid spiders follows the general organization in spiders [22]: paired testes and deferent ducts that fuse distally into an ejaculatory duct (Fig. 1). The testes are generally elongated and tubular (Fig. 1A, B, C, E), while Ninetinae show more compact to oval testes (Fig. 1D). Variation also occurs with respect to the morphology of the deferent ducts, which can be relatively short (Fig. 1A) to notably long (Fig. 1D). The testis is organized in cysts of developing spermatids (Fig. 2A, B), where different stages of spermatogenesis can be observed. The cysts are surrounded by extensions of the epithelial (somatic) cells, which border the lumen in the centre of the testis. The deferent ducts vary in the thickness of the epithelium (Fig. 2D vs. Fig. 3A), but generally have a layer of microvilli on the inner surface (Fig. 3D). The lumen of the deferent ducts contains the sperm transfer forms embedded in electron-dense secretions. The latter show an intergeneric diversity across the examined taxa (Figs. 4, 5 and 6). The ejaculatory duct shows a similarly organized epithelium and contains sperm and specific secretions as well (Fig. 3B).

## Spermiogenesis

**Fig. 7:**
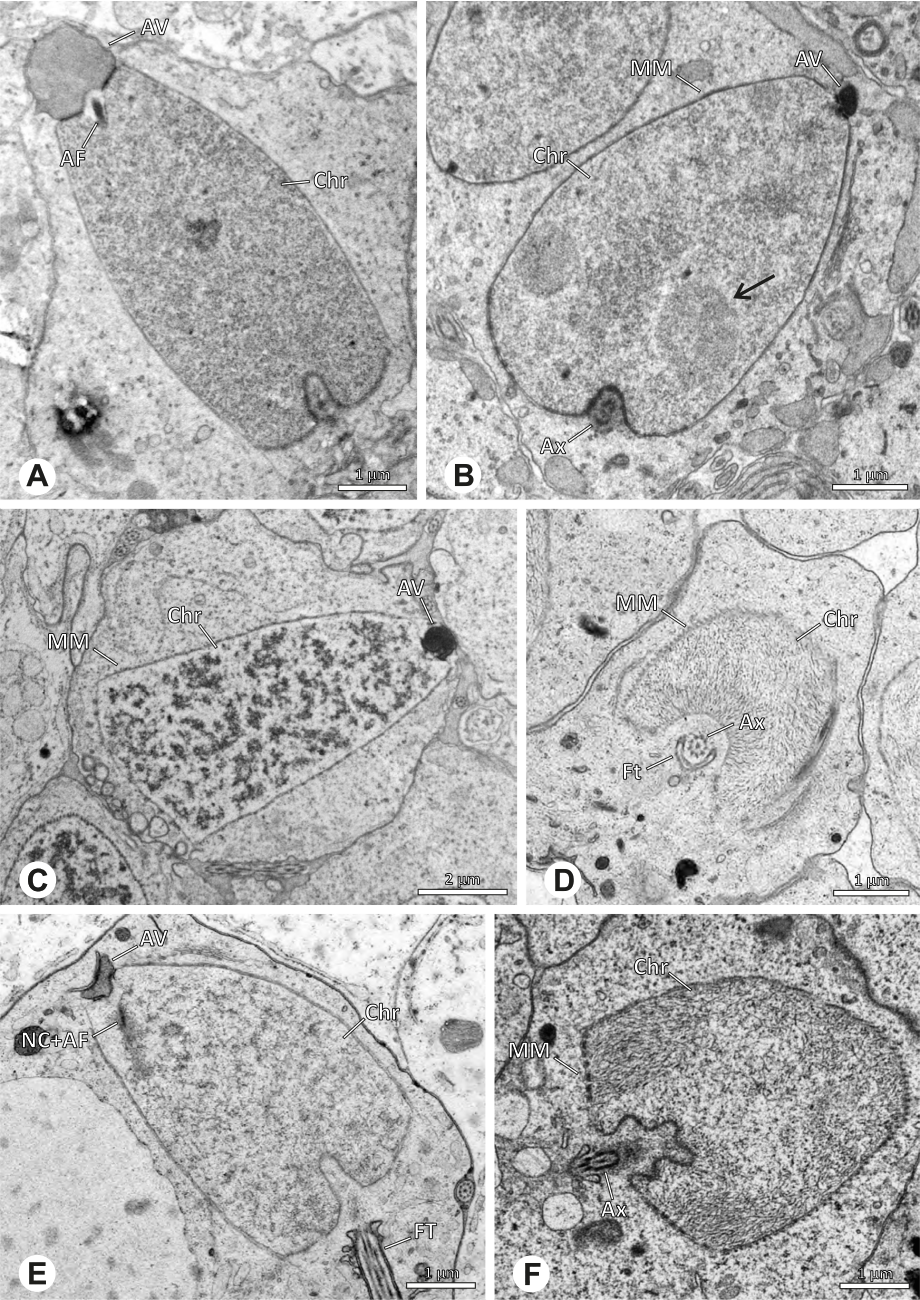
Early stages of spermiogenesis in different pholcid taxa. TEM. **A** *Priscula* sp. n. ‘Ecu93’*.***B** *Carapoia nairae.* The arrow indicates the globular condensation pattern of the chromatin. **C** *Chibchea salta*. **D** *Guaranita goloboffi*. **E** *Nerudia* sp. n. ‘Mic20’. **F** *Gertschiola macrostyla*

**Fig. 8:**
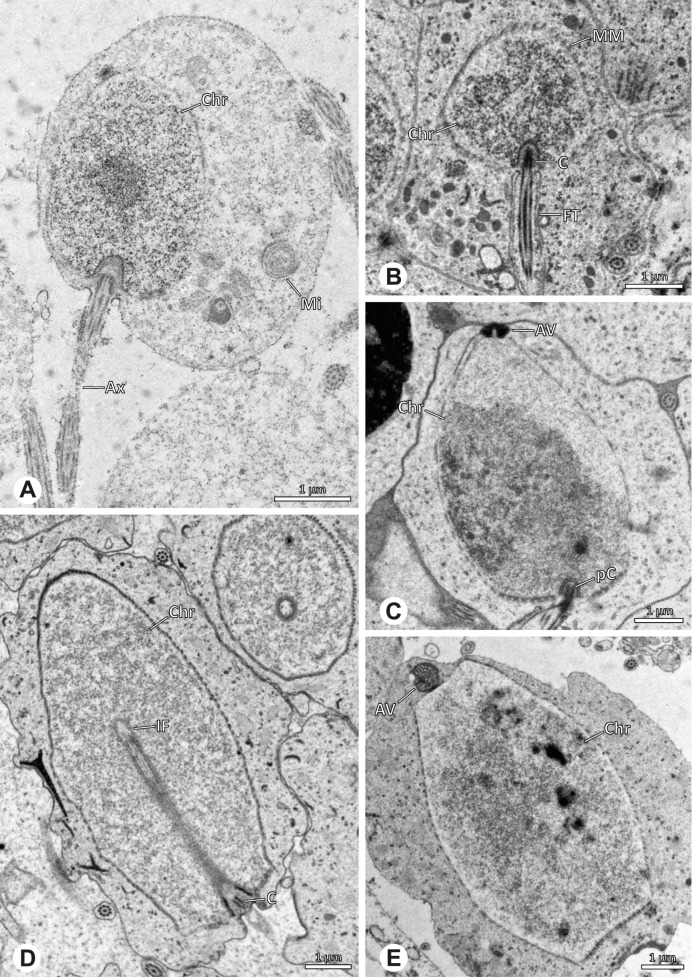
Early stages of spermiogenesis in different pholcid taxa. TEM. **A** *Panjange camiguin*. **B** *Spermophora awalai***C** *Pholcus bamboutos*. **D** *Smeringopina bineti*. **E** *Smeringopus cylindrogaster*. Note the uniform shape of spermatids in this stage

Spermiogenesis in the studied species follows a relatively uniform differentiation pattern. However, some species-specific deviations were observed and will be addressed in the species level descriptions below. Generally, spermatogenesis begins with the development of spermatogonia in the testis resulting in cysts of spermatids of the same developmental stage (Fig. 2A, B). In these early spermatids, the acrosomal vacuole and acrosomal filament begin to form, with the latter originating from electron dense material within the subacrosomal space. The acrosomal filament then begins to extend through the nucleus (Fig. 7). The condensation of nuclear chromatin progresses and the nucleus becomes more elongated while being surrounded by a manchette of microtubules (e.g., Fig. 7B, E). The condensation pattern of chromatin can vary among species from fibrillar (Fig. 7E) to globular (Fig. 7B). The distribution of strongly or less strongly condensed chromatin in the nucleus also varies: most investigated species show a uniform distribution throughout the nucleus, as for example *Artema bunkpurugu*, *Nerudia* sp. n. ‘Arg58’, or *Gertschiola macrostyla* (Figs. 7A, E, F); in others, we observed scattered (e.g., *Chibchea salta* Huber, 2000, Fig. 7C) or notably heterogenous distributions (e.g., *Pholcus bamboutos* Huber, 2011, Fig. 8C). The formation of the axoneme is initiated in early spermatids by the migration of the centrioles towards the posterior pole of the nucleus resulting in a flagellar tunnel (e.g. Figs. 7E and 8B). The implantation fossa forms as an indentation on the posterior pole of the nucleus (Figs. 7F and 8D) and its dimension and depth vary across species. Mid spermatids are characterized by a further elongation of the acrosomal vacuole (AV) with an extension of the subacrosomal space throughout the entire AV (Fig. 7A). The nucleus elongates asymmetrically and the chromatin condenses further and exhibits a mostly fibrillar condensation pattern (Fig. 7A).

**Fig. 9:**
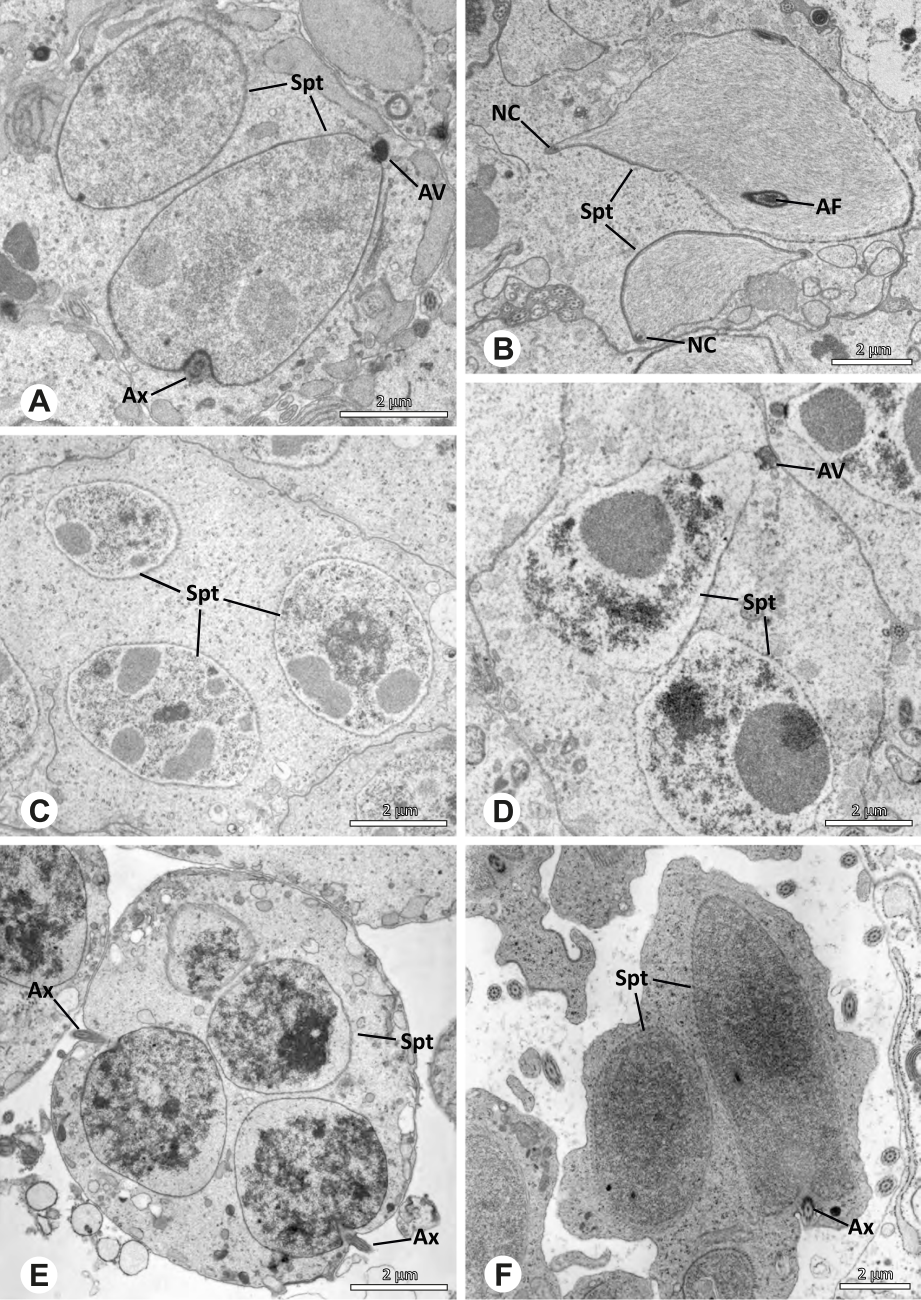
Initial fusion of early to mid spermatids during spermiogenesis in different pholcid species. Multiple spermatids were observed to remain fused during spermiogenesis before ultimately separating in later stages of development. **A** *Carapoia nairae*. **B** *Chibchea salta*. **C** *Mesabolivar iguazu*. **D** *Carapoia lutea*. **E** *Smeringopus* cf. *roeweri*, note the axonemes of two different spermatids. **F** *Holocnemus pluchei*

In several species, we observed an initial fusion of spermatids at early to mid stages (Fig. 9). The spermatids stay fused until they ultimately separate within the testes before the coiling process.

Late spermatids are characterized by a nucleus with a very dense chromatin condensation, which makes it appear electron dense. However, specific condensation patterns were observed, as e.g. the heterogenous condensation in *Mesabolivar cyaneotaeniatus* (Keyserling, 1891) (Fig. 36B, see below). At the end of spermiogenesis, the main cell components coil within the sperm cells. During this coiling process, which takes place in the testis, the manchette of microtubules is first reduced and then disintegrates completely in all studied species. The coiled spermatids finally compact and are surrounded by a secretion sheath. This sheath is usually formed in the deferent duct, where the sperm are also embedded in specific types of seminal secretions (Figs. 4, 5 and 6).

## Spermatozoa and transfer forms

Pholcid spiders show a high intrafamiliar disparity of sperm morphology, which will be addressed below in detail. In general, the spermatozoa follow the general pattern described for spiders in being flagellate with a long axoneme having a 9 + 3 microtubular pattern, and in having an asymmetrically elongated nucleus and an acrosomal vacuole, which is mostly cylindrical containing a narrow subacrosomal space. All investigated species have two centrioles orthogonally adjacent to each other. The centriolar region varies only in certain Pholcinae with a proximal centriole being twice the length of the distal centriole and electron-dense material that resemble a so-called “water-wheel” configuration (sensu [16]). Variations can be observed in all sperm cell components as e. g. the different parts of the nucleus or centriolar adjunct material. At the end of spermiogenesis, spermatozoa form so-called transfer forms, where sperm are encapsulated by a secretion sheath formed within the deferent ducts. Sperm are transferred as cleistospermia with the exception of most studied Ninetinae, which use synspermia as transfer form.

**Fig. 10:**
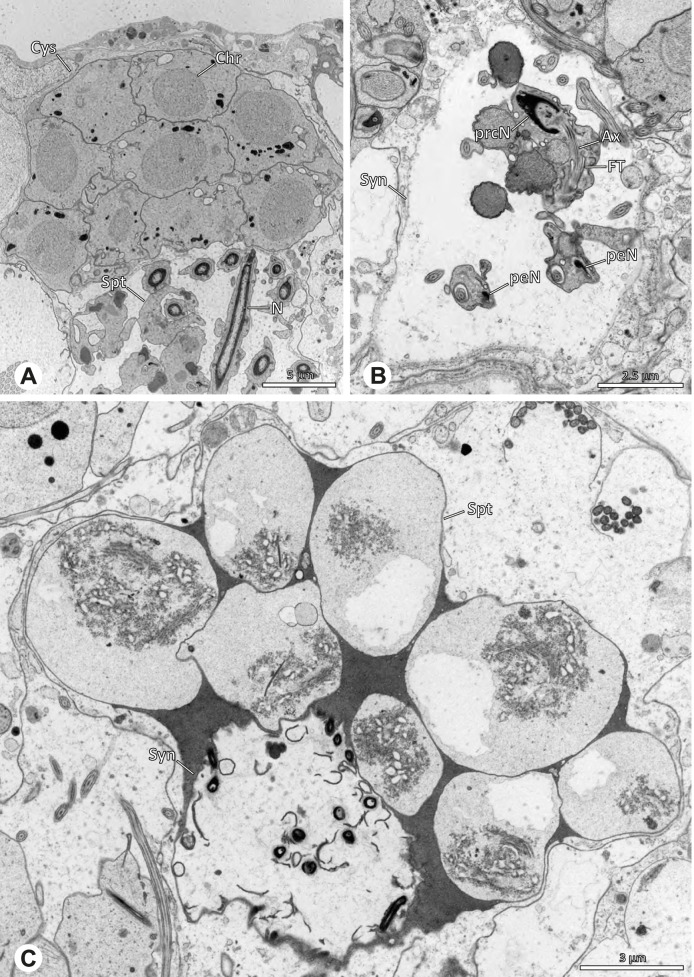
Formation of synspermia in *Guaranita goloboffi*. TEM. **A** Cysts of early and mid to late spermatids. **B** Multiple mid to late spermatids. **C** Fused late spermatids, accompanied by membranous remains of individual spermatids

**Fig. 11:**
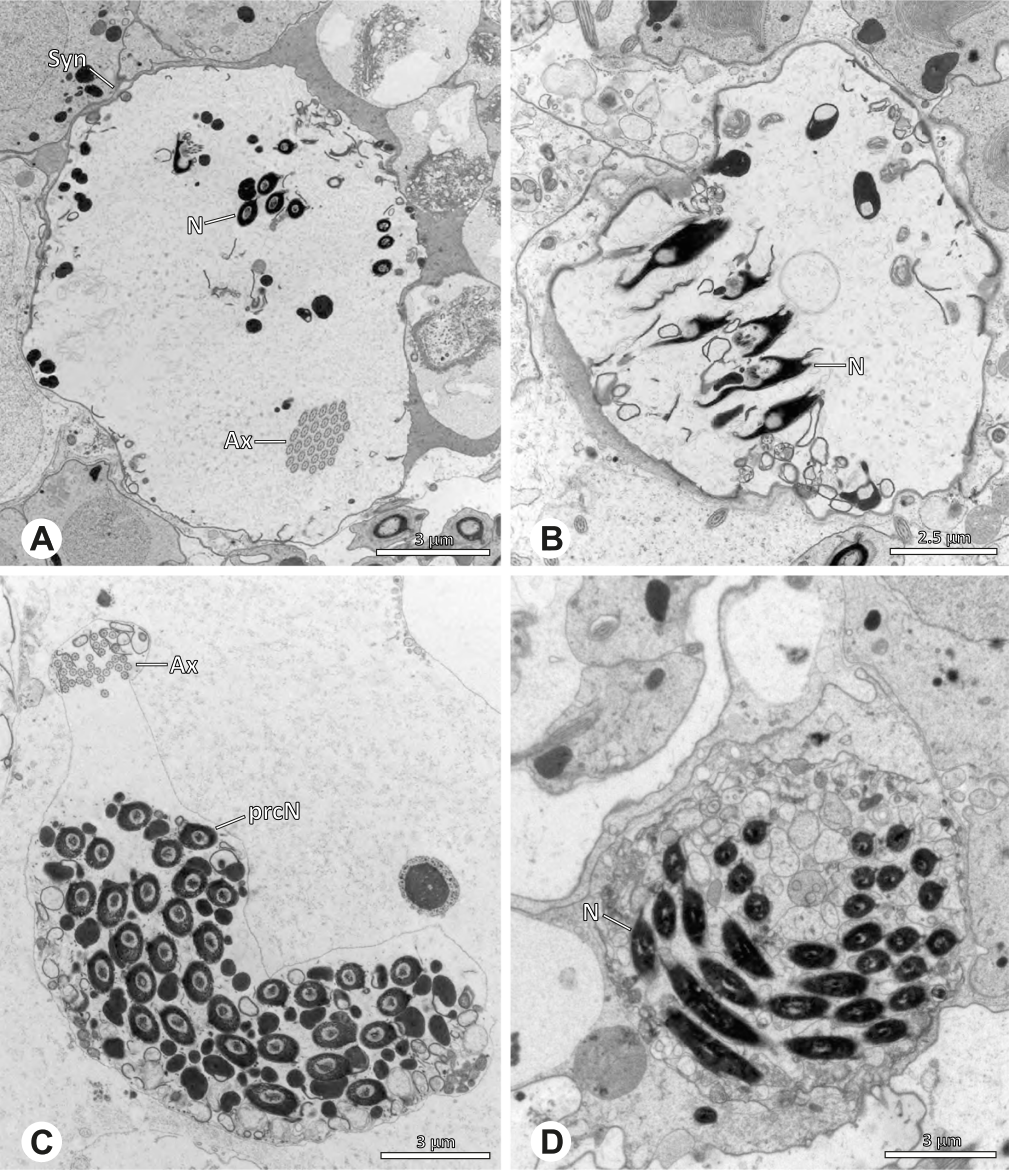
Formation of synspermia in *Guaranita goloboffi*. TEM. **A**, **B** Early synsperm with only loosely packed spermatids. **C**, **D** During further development, the spermatids become more densely packed within the synsperm. Note the increasing compactness of the synsperm and the occurrence of membranous remains and secretions in the cytoplasm

The formation of synspermia can be exemplified using *Guaranita goloboffi* Huber, 2000. As typical for spider spermatogenesis, spermatids develop within cysts (Fig. 10A) and are connected via cellular bridges as also shown in Costa-Ayub and Faraco [44] and Michalik, Dallai, Giusti and Alberti [45]. During the coiling process, spermatids fuse completely in the testis lumen, with part of their membranes disintegrating, leaving membranous remains in the cytoplasm of the aggregate (Figs. 10C and 11B). In further stages of the coiling process, the spermatids arrange more closely, nearly parallel to each other (Fig. 11C). Notably, the axonemes appear to be coiling altogether beside the nuclei, which remains in this configuration also in fully developed transfer forms (Fig. 16). During further coiling and compacting, the spermatids become densely embedded in the membranous remains and various secretions (Fig. 11D).

In the following, detailed descriptions of spermatozoa and sperm transfer forms are given ordered systematically by subfamily. Within the subfamily, species are ordered alphabetically. For species listed as ‘spp.’, individuals of different species have been investigated. Since the variation between those species was very low, the characters are summarized in one description and apply, if not stated otherwise, to all studied species.

### Ninetinae | *Galapa bella* (Gertsch & Peck, 1992) (Fig. 12)

**Fig. 12:**
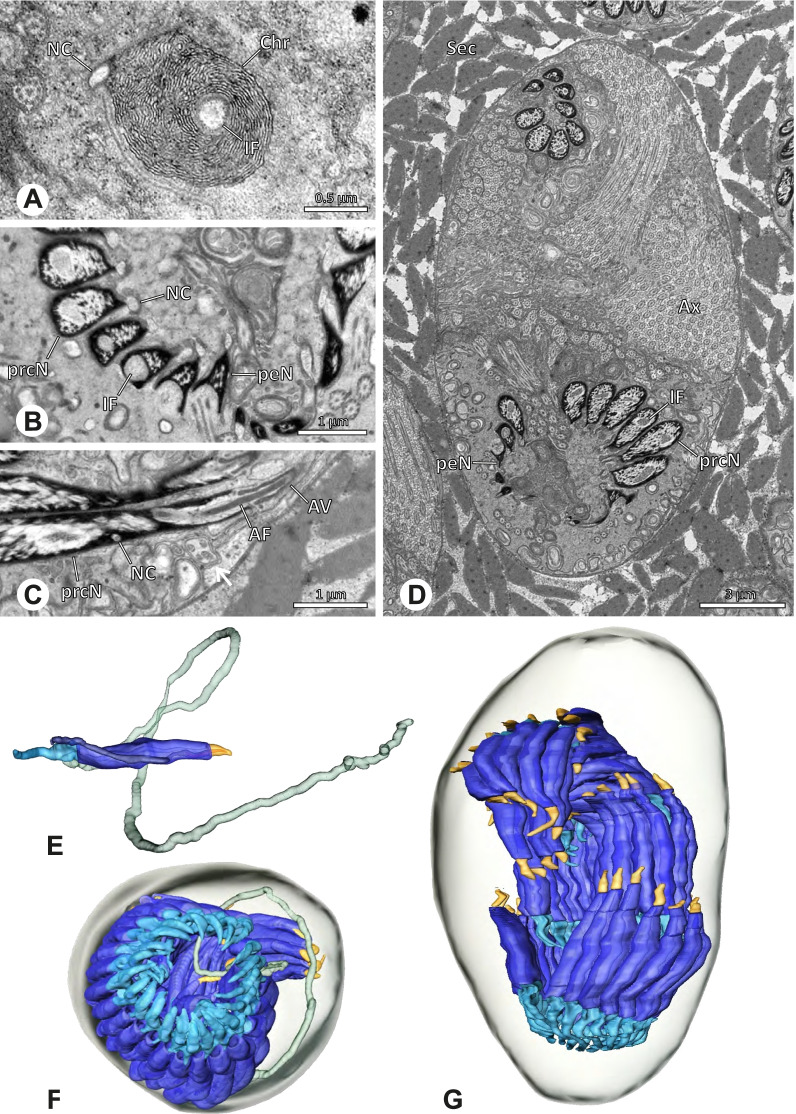
Spermiogenesis and synspermia of *Galapa bella*. TEM. **A** Early spermatid, cross-section. **B** Spermatozoa within synsperm. Note the shape of the peN and the position of the nuclear canal. **C** Anterior portion of a sperm cell within synsperm. Note the slightly conical shape of the AV and the membranous vesicles in the cytoplasm (arrow). **D** Synsperm within the deferent duct in cross-section. The thin secretion sheath becomes apparent as well as the tile-like seminal secretions. **E** 3D surface reconstruction of an individual sperm cell from the synsperm, illustrating further structural details as well as the course of the axoneme in a coiled state. **F**,**G** 3D surface reconstruction of the synsperm. For the purpose of clarity, only one axoneme is shown

**Spermatozoa.** Acrosomal complex. AV short, stout and conical, subacrosomal space extends throughout the entire AV (Fig. 12D). Acrosomal filament (AF) short, projecting into the nucleus through the nuclear canal (NC), ending in the anteriormost region of the precentriolar part of the nucleus (prcN) (Fig. 12D). Nucleus. Asymmetric, chromatin condensation heterogenous, centrally very lightly condensed and getting denser at the margin (Fig. 12B). prcN compact, posterior elongation of the nucleus (peN) slender and flat to triangular (Fig. 12E). Implantation Fossa (IF) narrow and deep, extends through the complete prcN, filled with granular material (Fig. 12B, C). NC wide, shifting into a lateral projection in the posterior most portion of the prcN as well as along the peN (Fig. 12B, C, D).

**Sperm transfer form.** Large, oval synspermia, surrounded by a secretion sheath; comprising 64 spermatozoa stacked on top of each other as groups of up to 23 densely and circular packed sperm cells in four levels (Fig. 12F). Axonemes tightly packed and coiling twice towards the center of the aggregate in a complex pattern (Fig. 12D). Cytoplasm heterogenous; mostly slightly electron dense, comprising various membranous vesicles (Fig. 12B, C).

**Notes on spermiogenesis.** Mid spermatids show a filamentous and streak-like chromatin condensation; the IF forms and begins to fill with electron dense granules; the NC forms and is shifted to the lateral portion of the nucleus (Fig. 12A).

**Seminal secretions.** One type of secretion, homogenously electron dense, long to tile-like (Fig. 5G).

### Ninetinae | *Gertschiola macrostyla* (Mello-Leitão, 1941) (Figs. 13 and 14)

**Fig. 13:**
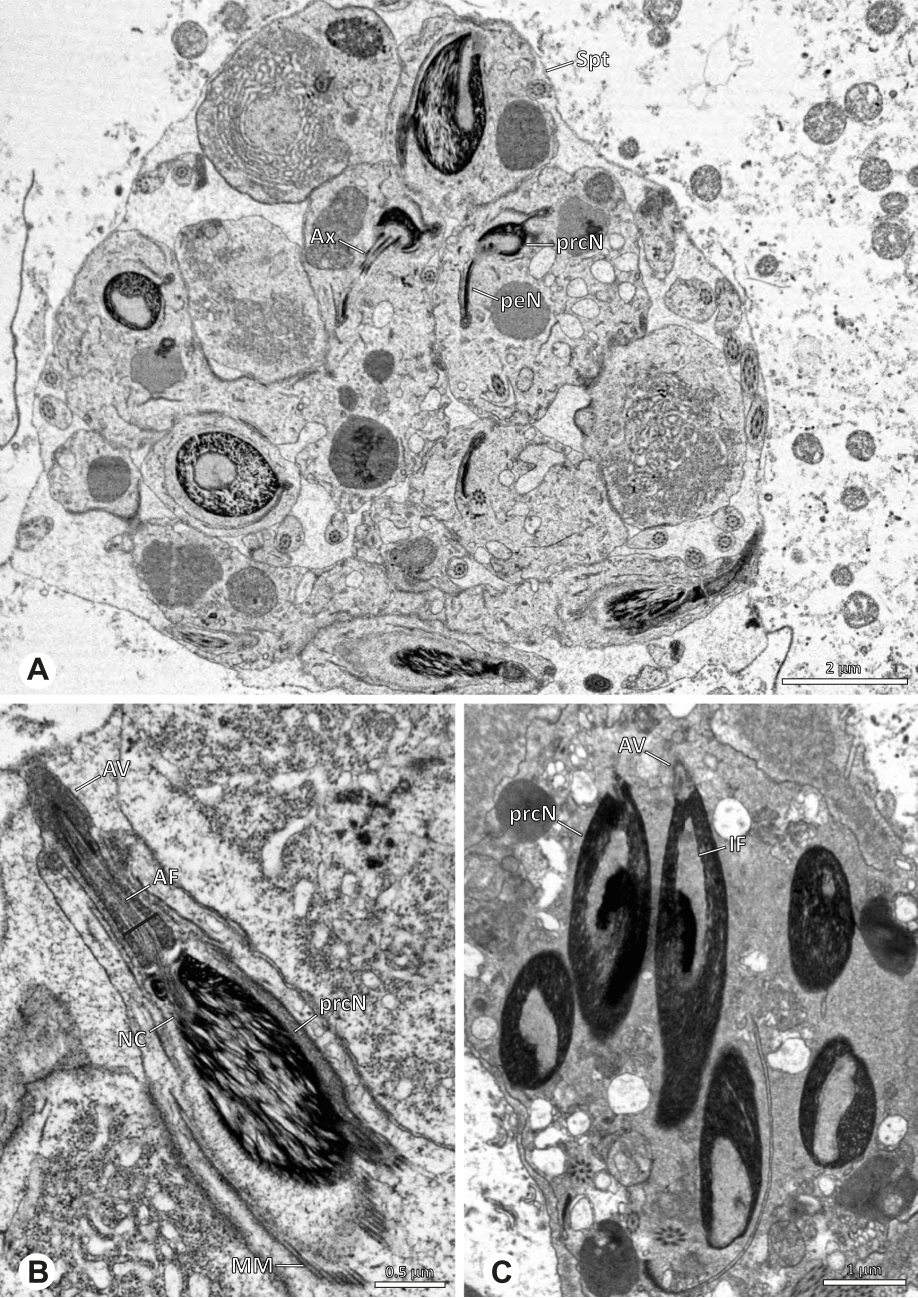
Spermiogenesis of *Gertschiola macrostyla*. TEM. **A** Mid spermatids in a cyst, individually surrounded by a membrane. **B** Mid spermatid, anterior portion with acrosomal complex. **C** Multiple late spermatids before the coiling process takes place

**Fig. 14:**
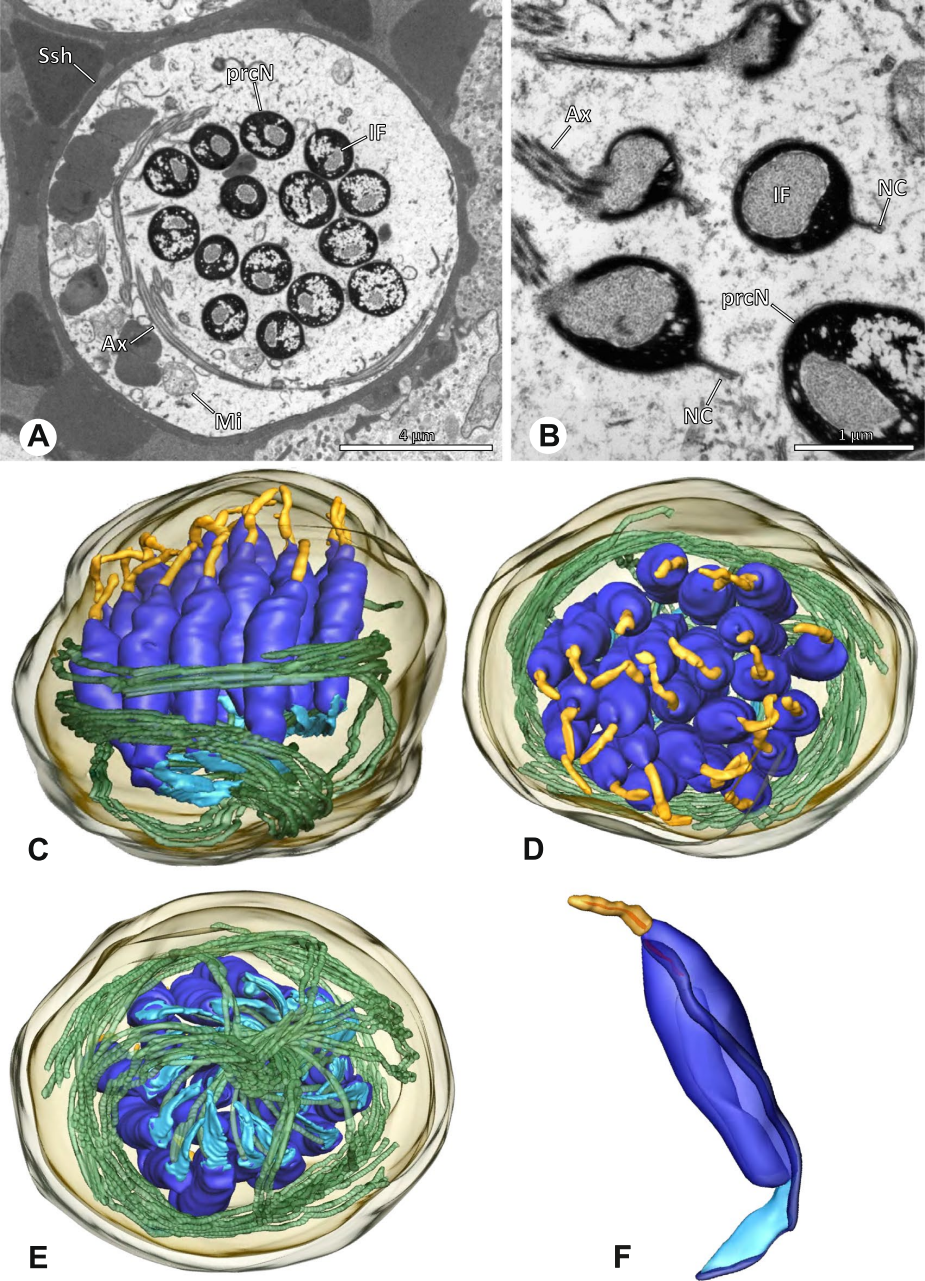
Ultrastructure and 3D surface reconstruction of synsperm of *Gertschiola macrostyla*. **A** Synsperm in the lumen of the deferent duct. **B** Sperm cells within the synsperm in cross-section. Note the position of the nuclear canal. **C**, **D**, **E** 3D surface reconstruction of synsperm. **F** 3D surface reconstruction of an individual sperm cell showing the depth of the implantation fossa and the course of the nuclear canal. Axoneme not shown for the purpose of clarity

**Spermatozoa.** Acrosomal complex. AV thin, subacrosomal space extends throughout the whole AV. The AF terminates in the anteriormost portion of the NC. Nucleus. Asymmetric, chromatin condensation heterogenous (Fig. 14A, B); prcN stout and tubular (Fig. 14C, E); peN approximately half the length of the prcN, flat and triangular (Fig. 14F). IF deep and narrow, extends nearly through the entire prcN, filled with granular material (Fig. 14B). NC situated in a thin lateral projection (Fig. 14B).

**Sperm transfer form.** Spherical to oval synspermia surrounded by a secretion sheath (Fig. 14A), comprising a group of 24 spermatozoa (Fig. 14C, D). AV rather straight within the aggregate (Figs. 13B, 14C, D), peN all bent towards the center of the nucleus cluster (Fig. 14E). The axonemes are projecting towards the center of this cluster in their anterior part, before forming multiple larger bundles that continue to coil once and form an interwoven pattern beside the nuclei (Fig. 14C, E), before coiling twice spirally around the cluster of sperm cells (Fig. 14D).

**Notes on spermiogenesis.** At late stages of spermiogenesis, the IF partially contains amorphous, electron-dense material (Fig. 13C).

**Seminal secretions.** One type of secretion, oval and homogenously electron dense (Fig. 5).

### Ninetinae | *Guaranita goloboffi* Huber, 2000 (Figs. 15 and 16)

**Fig. 15:**
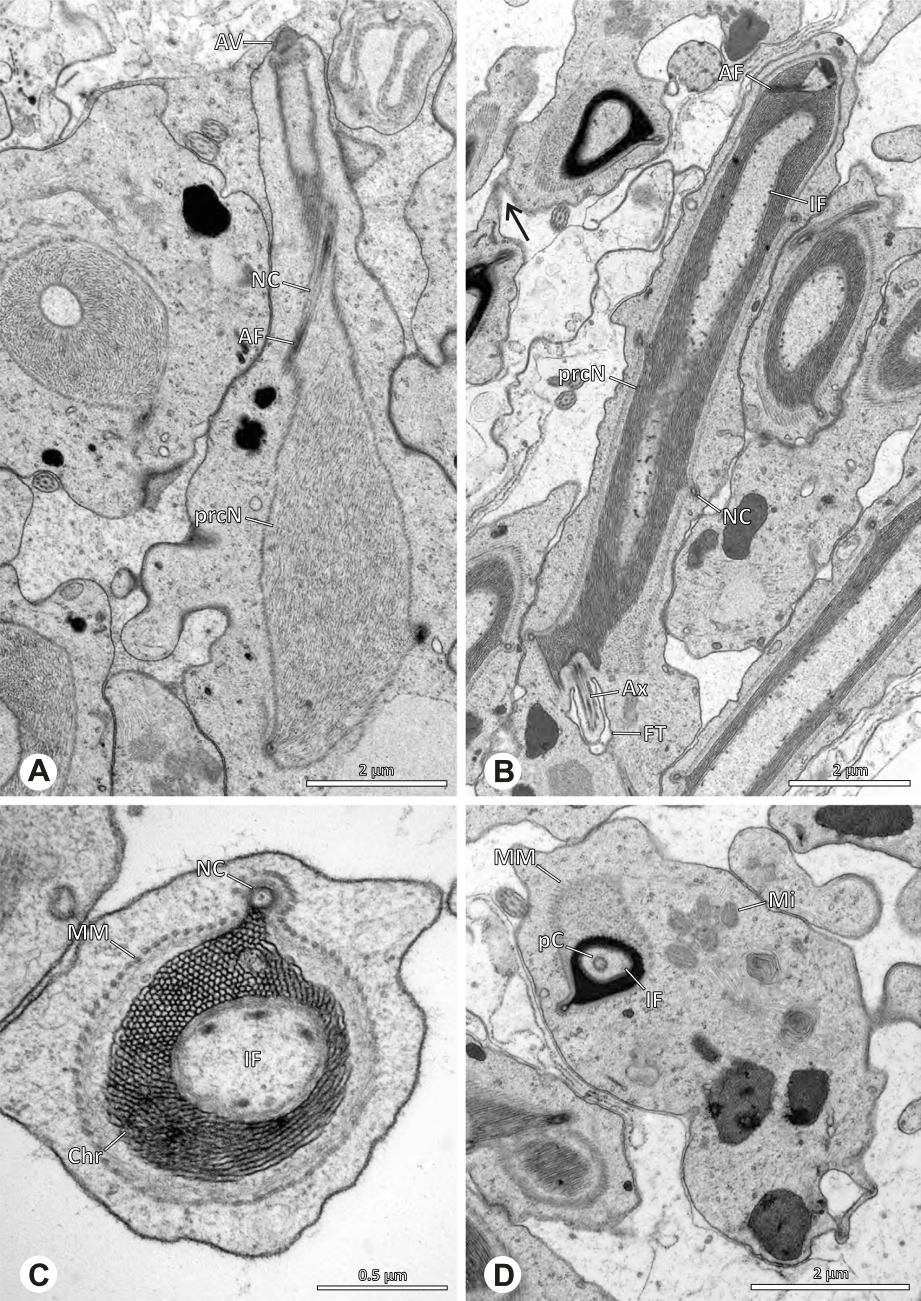
Spermiogenesis of *Guaranita goloboffi*. TEM. **A** Early spermatid, longitudinal section. **B** Mid spermatid, longitudinal section. The deep IF becomes apparent. **C** Mid spermatid, cross-section. Note the condensation pattern of the chromatin as well as the position of the nucleus, which begins to shift into a lateral projection. **D** Late spermatid, shortly before the coiling process

**Fig. 16:**
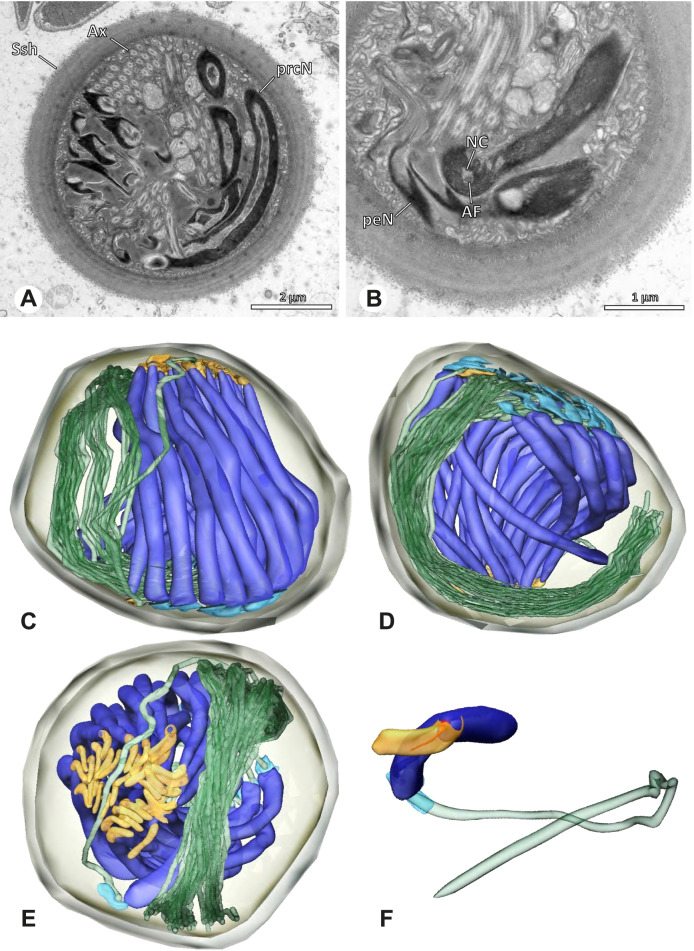
Ultrastructure and 3D surface reconstruction of the synsperm of *Guaranita goloboffi*. **A** Synsperm in the lumen of the deferent duct. Note the multilayered secretion sheath. **B** Sperm cells within the synsperm. Note the triangular shape of the peN. **C** , **D**, **E** 3D surface reconstruction of the synsperm. **F** Individual sperm cell

**Spermatozoa.** Acrosomal complex. AV short, cylindrical to flat (Fig. 16E, F), subacrosomal space extends throughout the whole AV (Fig. 16F). AF short extends only into the anteriormost portion of the NC (Fig. 15B) Nucleus. Asymmetric, prcN long, slender and cylindrical, peN short, flat and triangular to pointed (Fig. 16C, D). Chromatin condensation throughout the nucleus homogenous and dense. IF deep, extends throughout the whole prcN and filled with thin filamentous material (Figs. 15B and 16A). NC narrow, runs laterally through the prcN and shifting partly into a lateral projection helically winding along the nucleus (Fig. 15C, D).

**Sperm transfer form.** Spherical synspermia, surrounded by a secretion sheath and comprising 32 spermatozoa (Fig. 16). AV bent on top of the nuclei and peN bent inwards pointing towards each other (Fig. 16E). Axonemes tightly packed and coiled once beside the nuclei (Fig. 16D, E). Cytoplasm homogenous and electron-dense.

**Notes on spermiogenesis.** In mid to late spermatids, the condensation pattern is filamentous to streak-like, the nucleus is tube-like, long, and contains the prominent deep IF, which is filled with some filamentous material along its inner edge (Fig. 15B).

**Seminal secretions.** One type of secretion, long to triangular, electron dense center with an electron lucent margin (Fig. 5J).

### Ninetinae | *Kambiwa neotropica* (Kraus, 1957) (Figs. 17 and 18)

**Fig. 17:**
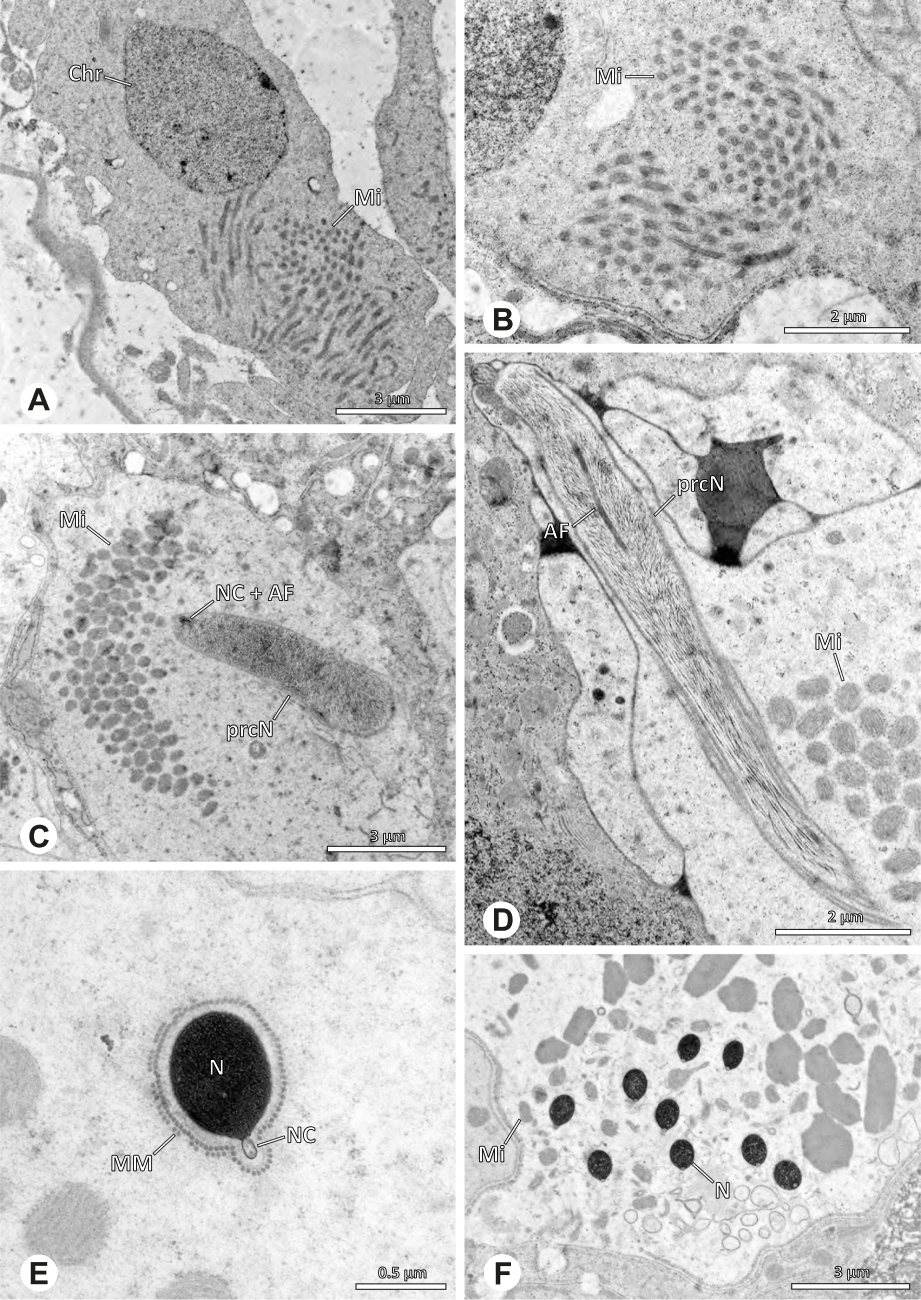
Spermiogenesis of *Kambiwa neotropica*. TEM. **A**, **B** Early spermatid. Note the localized accumulation of mitochondria. **C** Mid spermatid. The mitochondria concentrate at the anterior pole of the spermatid. **D** Mid to late spermatid. **E** Late spermatid, cross section. **F** Late spermatids before coiling. The mitochondria are less localized but distributed through the cytoplasm

**Fig. 18:**
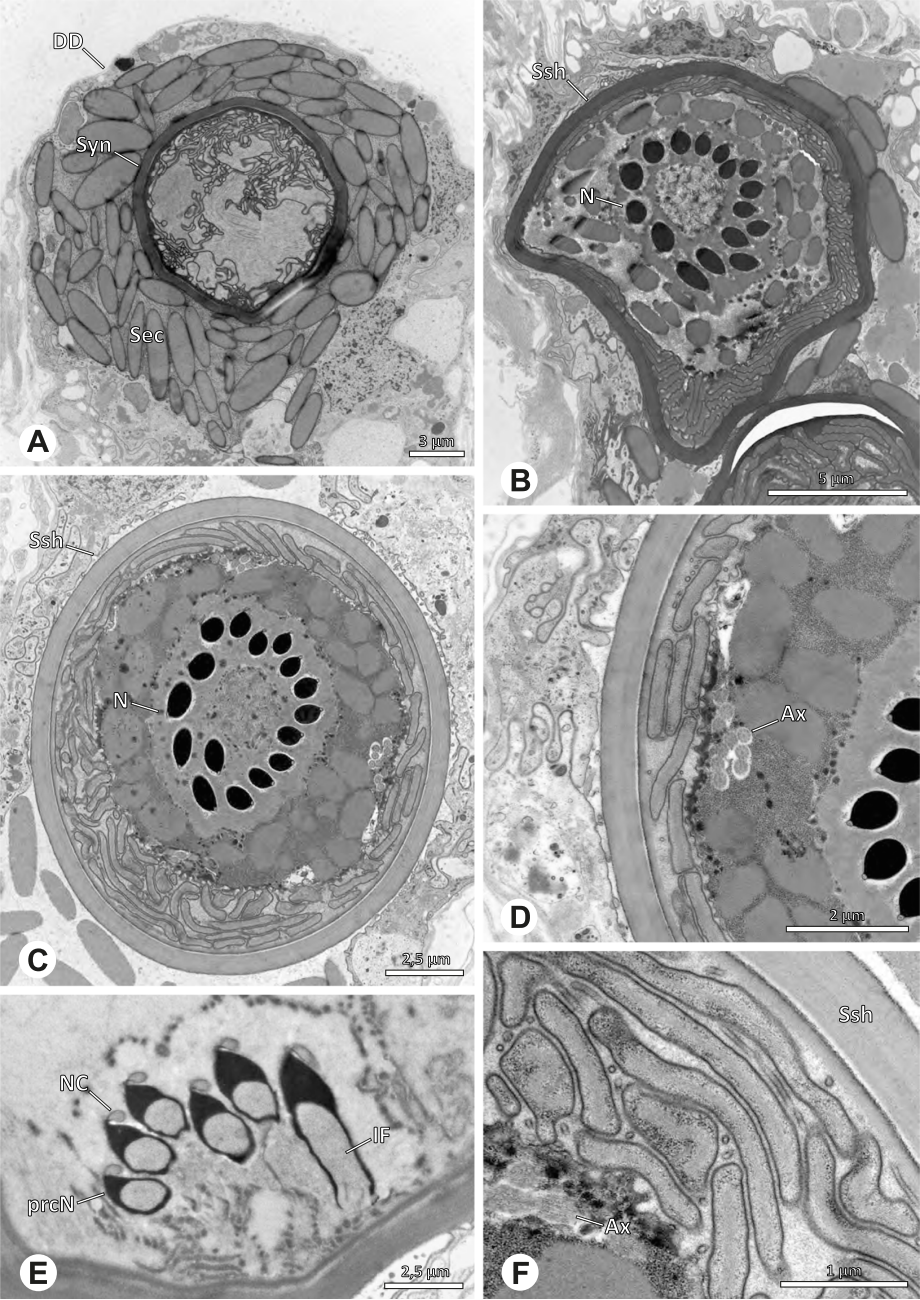
Synsperm of *Kambiwa neotropica*. TEM. **A** Cross-section through the anterior pole of the synsperm. Note the membranous inner structures as well as the dense secretion sheath and the tile-like seminal secretions. **B**, **C** Cross sections through synspermia showing the flexibility of the aggregate as well as of the various internal structures. **D** Detail of the layering of internal secretions and filamentous structures surrounding the sperm cells within the synsperm. **E** Sperm cells within the synsperm. Note the position of the nuclear canal within a fine projection. **F** Detail of the filamentous structures beneath the secretion sheath

**Spermatozoa.** Acrosomal complex. AF ends clearly before the axonemal basis. Nucleus. Long, slender, and asymmetric (Fig. 18B), with densely condensed homogenous chromatin. NC narrow, situated laterally in the anterior portion of the prcN and shifting into a short lateral projection while running beside the IF (Fig. 18E). IF extends through approximately half of the prcN.

**Sperm transfer form. **Spherical synspermia with a secretion sheath, comprising 16 spermatozoa, which are embedded in the cytoplasm in a heterogenous, electron dense, secretion-like matrix, that itself is surrounded by globular secretory droplets and distinct filamentous structures in the peripheral area, appearing to form an inner cover of “tiles” beneath the outer secretion sheath of the aggregate (Fig. 18A, C, D, F).

**Notes on spermiogenesis. **Chromatin condensation begins with densely condensed patches in the periphery of the forming nucleus (Fig. 17A). During further development, the nucleus becomes very elongated and slender, with a light, streak-like condensation pattern in mid spermatids (Fig. 17D). At this stage, the NC with the AF is situated at least partially in the center of the developing nucleus (Fig. 17D). The long and slender shape of the nucleus is also observable in late spermatids (Fig. 17E in cross-section). The NC is shifted to the lateral margin of the nucleus. Mid spermatids are further characterized by a prominent aggregation of numerous mitochondria which are situated in the posterior part of the spermatid (Fig. 17B, C).

**Seminal secretions.** One type of secretion, long and tile-like (Fig. 5G).

### Ninetinae | *Nerudia* spp. (Figs. 19 and 20)

**Fig. 19:**
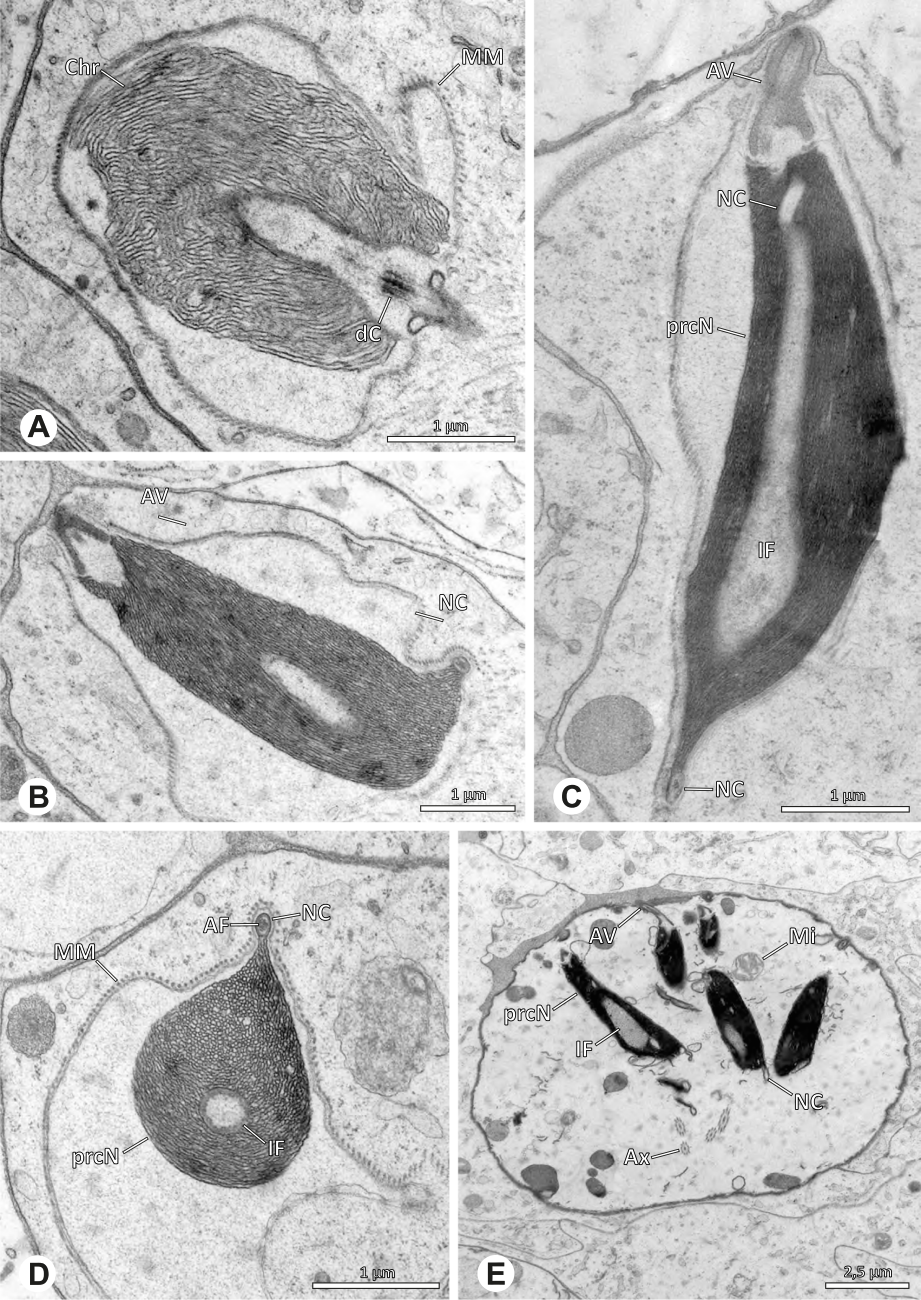
Spermiogenesis of *Nerudia* sp. n. ‘Mic20’. TEM. **A** Early spermatid. Note the condensation pattern. **B**-**D** Late spermatids. **E** Coiled and conjugated spermatids in the lumen of the testis

**Fig. 20:**
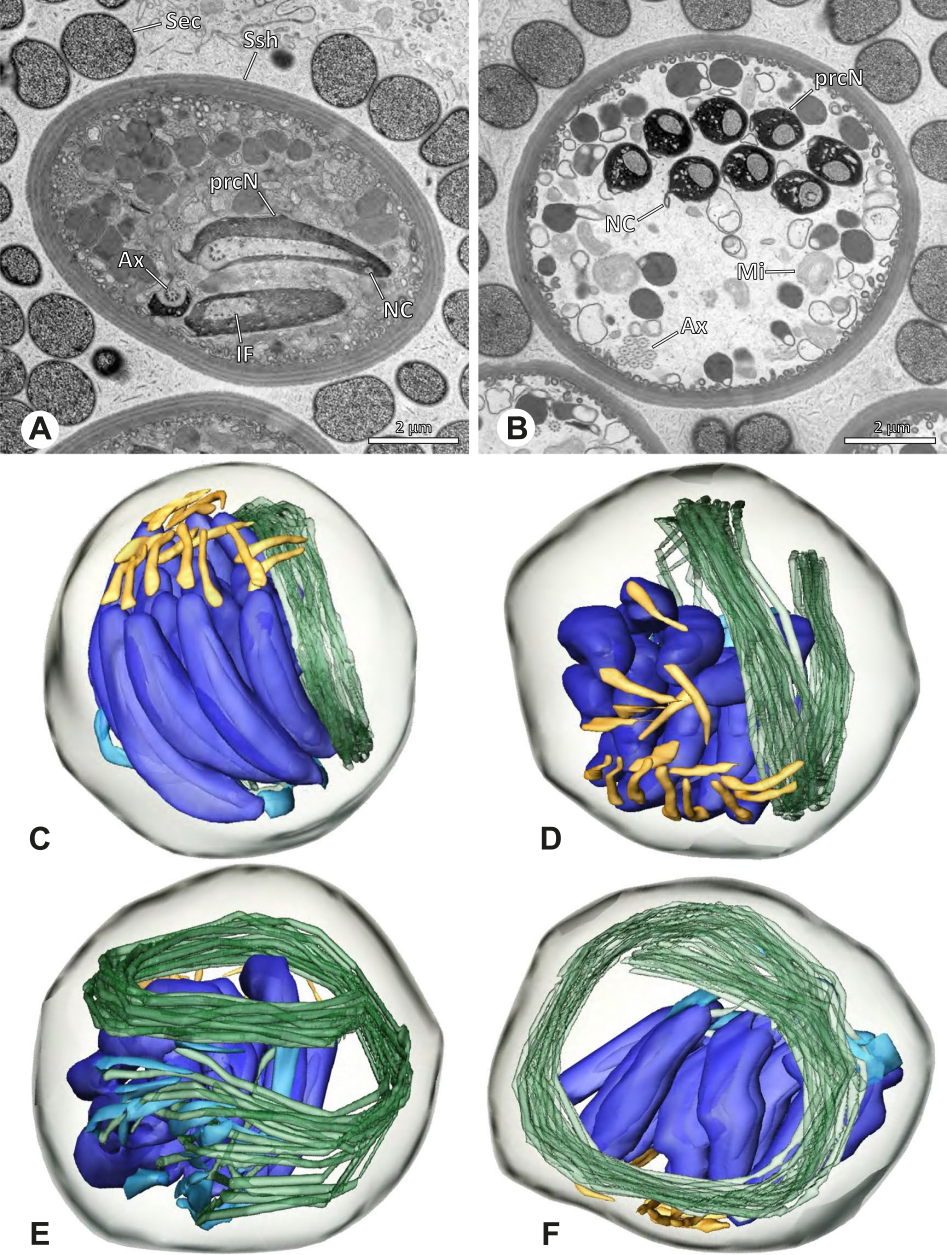
Ultrastructure and 3D surface reconstruction of the synsperm of *Nerudia* spp. **A** *Nerudia *sp. n. ‘Mic20’. Synsperm in the lumen of the deferent duct, longitudinal section. **B** *Nerudia* sp. n. ‘Mic20’. Synsperm in the lumen of the deferent duct, cross-section. The one-sided arrangement of sperm within the synsperm is well visible. **C**, **D**, **E**, **F** *Nerudia* sp. n. ‘Arg58’. 3D surface reconstruction of a synsperm

**Spermatozoa.** Acrosomal complex. AV slender and cylindrical, subacrosomal space extends throughout the whole AV (Fig. 19C). AF short, projecting only into the anteriormost portion of the NC. Nucleus. Asymmetric, prcN cylindrical and compact, with partially heterogeneously condensed chromatin (Fig. 20B). peN short, less than half the length of the prcN, flat to triangular in cross-section (Fig. 20E). IF deep and narrow, extends throughout nearly the entire prcN and filled with granular material (Figs. 19C and 20B). NC narrow, located laterally along the nucleus in a thin projection (Figs. 19C, E and 20B).

**Sperm transfer form. **Spherical synspermia with a secretion sheath (Fig. 20A, B), comprising 16 spermatozoa. The sperm cells are aligned nearly in parallel and packed laterally in the synsperm and remain nearly uncoiled to slightly bent at most (Fig. 20C). The axonemes of all sperm cells are compactly united and coil once spirally beside the nuclei (Fig. 20D, E, F). Cytoplasm heterogenous, containing electron dense secretions, electron lucent vesicles and mitochondria (Fig. 20A, B).

**Notes on spermiogenesis.** In mid- to late spermatids, the chromatin condenses in a fibrillar pattern, while it is only loosely associated with the manchette of microtubules (MM); the NC starts to form in a lateral position within the nucleus (Fig. 19A). In late spermatids, the IF begins to comprise fine electron dense granular material (Fig. 19C). During the coiling process, the NC shifts further laterally, into the abovementioned lateral projection (Fig. 19D).

**Seminal secretions**. One type of secretion, globular and heterogenous (Fig. 5G).

### Ninetinae | *Pholcophora* spp. (Figs. 21 and 22)

**Fig. 21:**
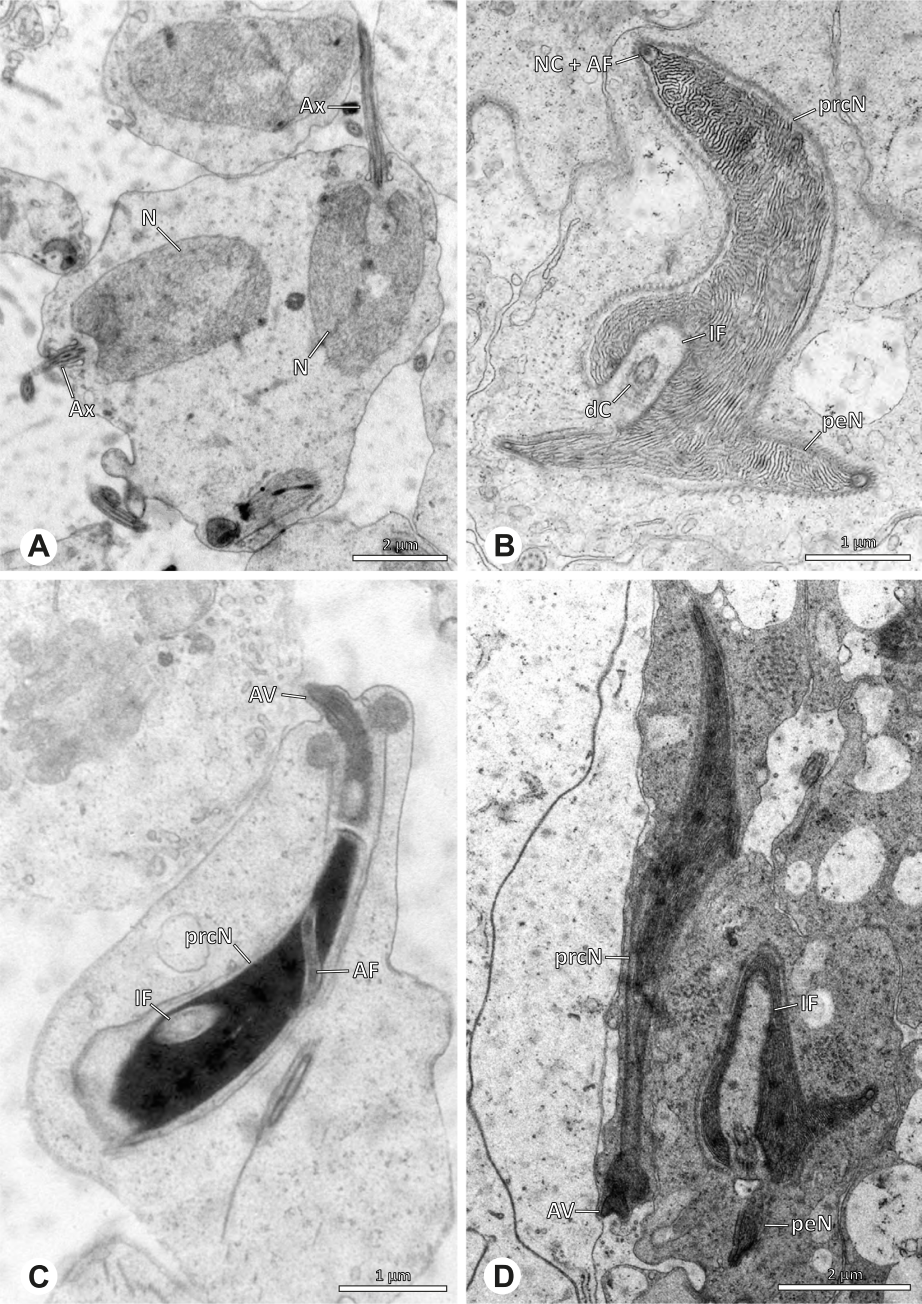
Spermiogenesis of *Pholcophora* spp. TEM. **A** *Pholcophora* sp. n. ‘Mex157’. Early spermatids are fused during spermiogenesis. **B** *Pholcophora* sp. n. ‘Mex22’. Mid spermatid. Note the pointy triangular appearance of the nucleus. **C** *Pholcophora* sp. n. ‘Mex157’. Mid to late spermatid. Note the position of the AF in the periphery of the nucleus and the cylindrical AV. **D** *Pholcophora* sp. n. ‘Mex22’. Late spermatids still remain fused and separate before the coiling process to ultimately form cleistospermia

**Fig. 22:**
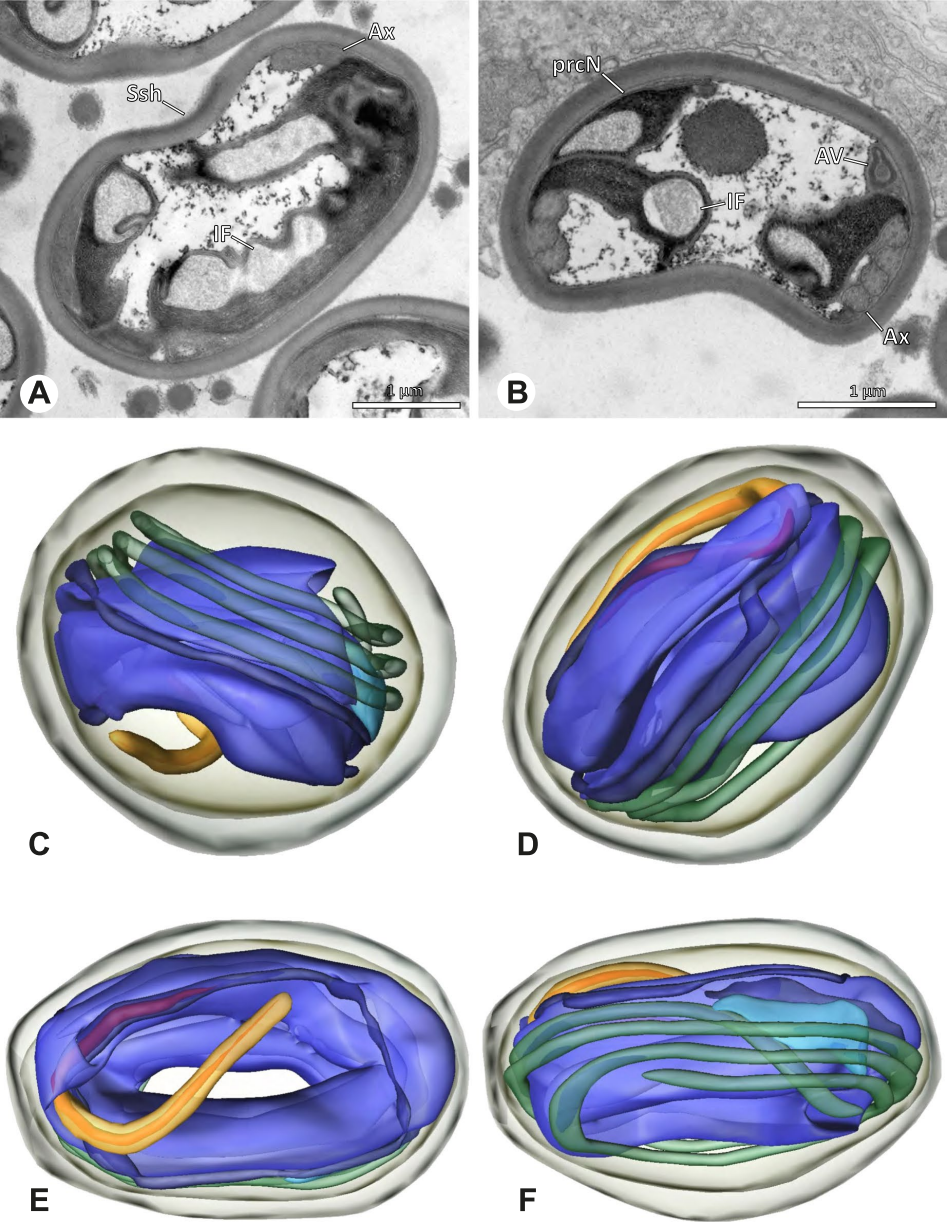
Ultrastructure and 3D surface reconstruction of cleistospermia of *Pholcophora* sp. n. ‘Mex22’. **A**, **B** Cleistospermia in the lumen of the deferent duct. Note the pointy shape of the prcN in (**B**). **C**, **D**, **E**, **F** 3D surface reconstruction of a cleistosperm

**Spermatozoa.** Acrosomal complex. AV long and very slender, with the subacrosomal space extends throughout the entire AV (Figs. 21C and 22E). AF widens towards posterior. AF ends within the anterior quarter of the prcN. Nucleus. Asymmetric, prcN long, triangular in cross-section (Fig. 22D). IF deep, extends nearly through the whole prcN, narrow in its anteriormost portion and filled with granular material (Fig. 22B). peN short, flat to triangular (Fig. 22F). NC projects laterally through the nucleus (Figs. 21C and 22B, F).

**Sperm transfer form.** Oval cleistospermia, surrounded by a secretion sheath (Fig. 22A, B). Nucleus coiled multiple times, with the AV situated alongside the first turn (Fig. 22D). Ax coiled three times around the posterior portion of the nucleus (Fig. 22C, F).

**Notes on spermiogenesis.** Spermatids appear to fuse completely during early spermiogenesis and to separate later (Fig. 21A). Mid spermatids become elongated and show a chromatin condensation in a spiral, nearly labyrinth-like manner (Fig. 21A, B). The prcN and peN develop a pointy, triangular shape at this state (Fig. 21B, D).

**Seminal secretions.** One type of secretion, small globular, homogenously electron dense (Fig. 5A).

### Ninetinae | *Tolteca* spp. (Figs. 23 and 24)

**Fig. 23:**
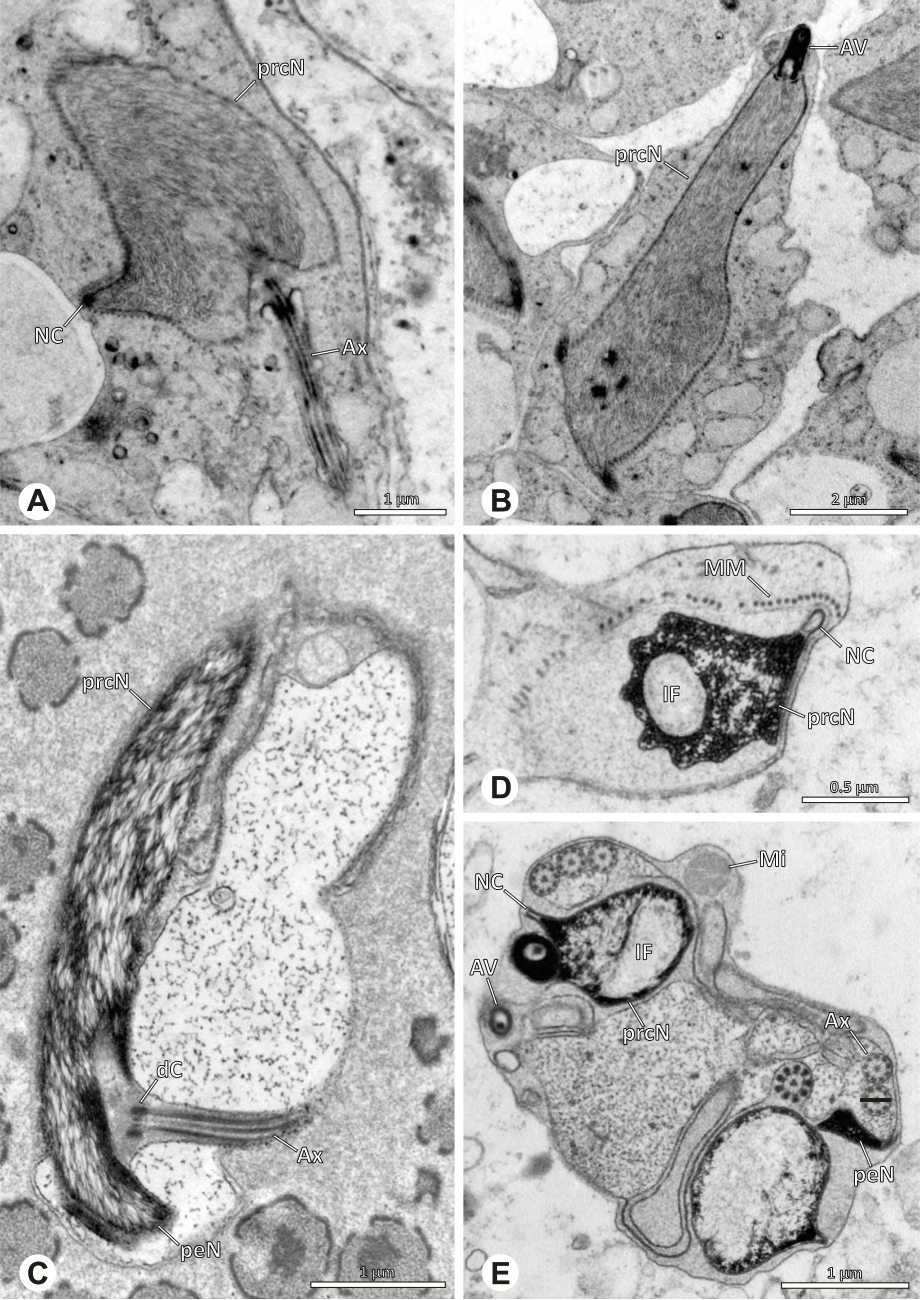
Spermiogenesis of *Tolteca *spp. TEM. **A** *Tolteca *sp. n. `Mex 169`. Early spermatid. Note the elevated NC. **B** *Tolteca *sp. n. `Mex 169`. Mid spermatid. **C** *Tolteca *sp. n. `Mex 169`. Late spermatid. Note the electron lucent cytoplasm. **D** *Tolteca hesperia*. Late spermatid, cross-section. Note the conspicuous longitudinal ridges along the prcN. **E** *Tolteca hesperia*. Coiled spermatid in the lumen of the testes. Note the heterogenous chromatin condensation pattern of the nucleus

**Fig. 24:**
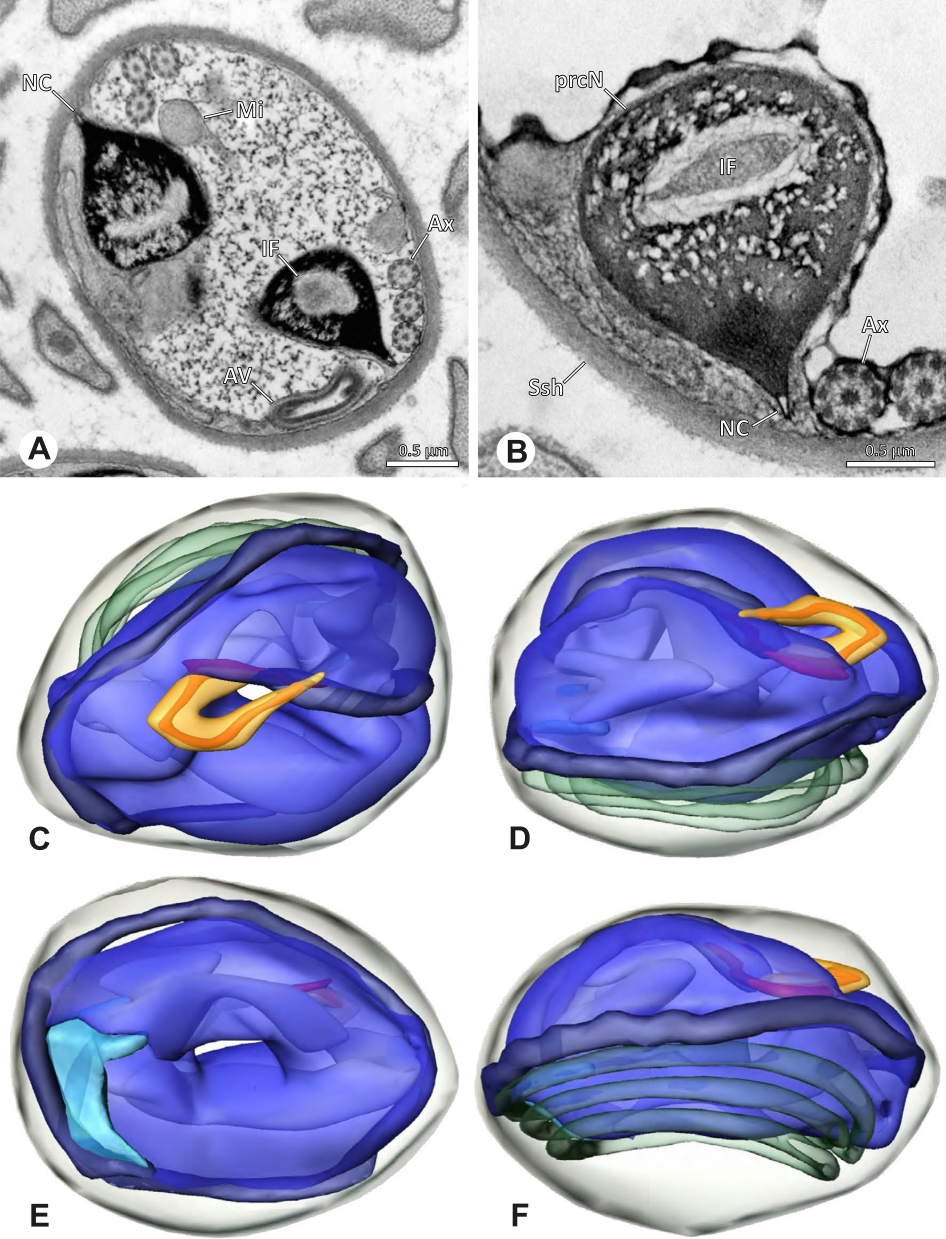
Ultrastructure and 3D surface reconstruction of cleistospermia of *Tolteca* spp. **A** *Tolteca* sp. n. ‘Mex169’. Cleistosperm in the lumen of the deferent duct. **B** *Tolteca* sp. n. ‘Mex169’. Cleistosperm, detail. Note the position of the nuclear canal and the fine granular content of the IF. **C**, **D**, **E**, **F** *Tolteca hesperia*. 3D surface reconstruction of a cleistosperm

**Spermatozoa.** Acrosomal complex. AV conical, subacrosomal space extends throughout the AV (Fig. 24C). AF terminates within the anterior third of the NC (Fig. 24F). Nucleus. Asymmetric, chromatin condensation heterogenous, with densely and only lightly condensed areas especially around the margin of the IF (Fig. 24B). prcN broad and drop-shaped in cross section (Fig. 24B). peN short, flat and triangular (Fig. 24E). IF deep, extends through the entire prcN (Fig. 24D) and filled with fine granular material (Fig. 24B). Thin NC projecting alongside the nucleus in a small lateral projection (Fig. 24A, B).

**Sperm transfer form.** Spherical cleistospermia, surrounded by a secretion sheath (Fig. 24A, B). prcN filling most of the transfer form. AV bent on top of the coiled nucleus (Fig. 24D). peN bent alongside the prcN (Fig. 24E). Ax coiled three times, partially around the nucleus (Fig. 24D, F). Cytoplasm heterogenous, mitochondria present (Fig. 24 A).

**Notes on spermiogenesis.** Late spermatids show longitudinal ridges in the condensed part of the nucleus, best observable in cross-section (Fig. 23D). The NC forms a thin lateral projection alongside the nucleus. During the coiling process the cytoplasm of the forming cleistosperm becomes notably electron lucent (Fig. 23C).

**Seminal secretions.** One type of secretion, slender to triangular, heterogenous with electron dense patches (Fig. 5L).

### Arteminae | *Artema bunkpurugu* Huber & Kwapong, 2013 (Figs. 25 and 26)

**Fig. 25:**
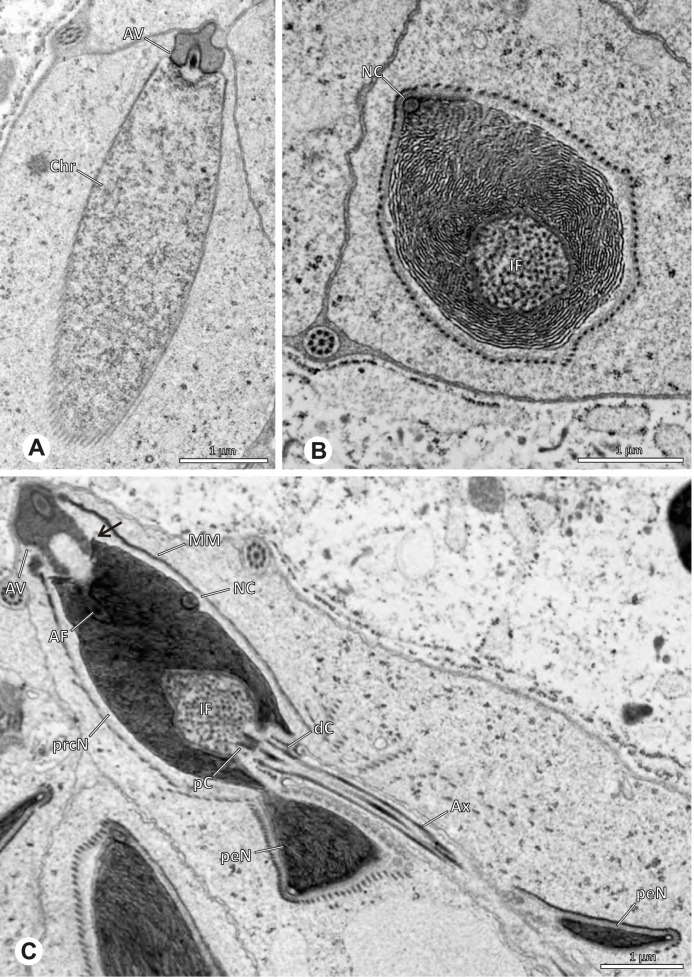
Spermiogenesis of *Artema bunkpurugu*. TEM. **A** Early spermatid. **B** Mid spermatid, cross section. Note the granular content of the IF and the condensation pattern of the nucleus. **C** Late spermatid, longitudinal section

**Fig. 26:**
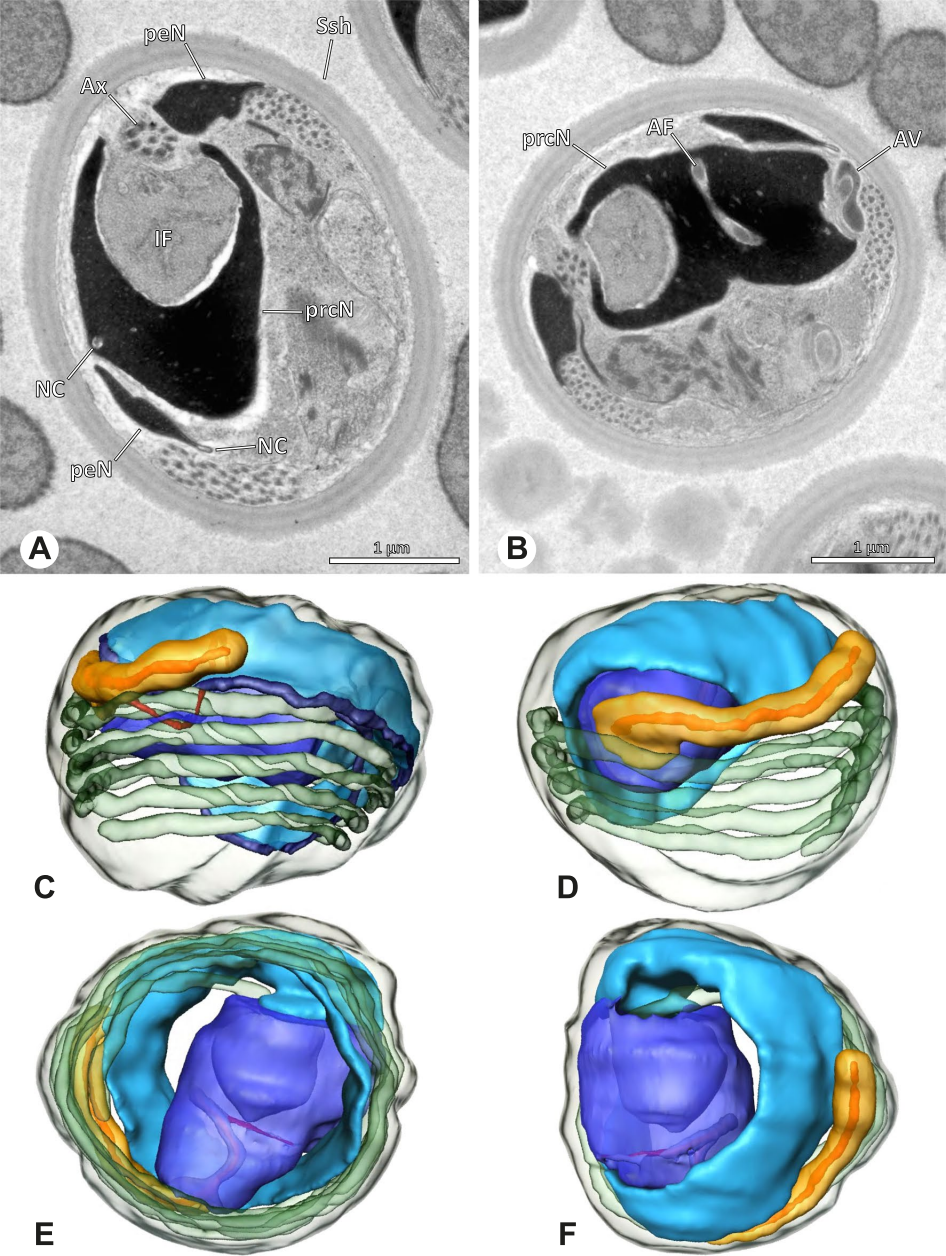
Ultrastructure and 3D surface reconstruction of cleistospermia of *Artema bunkpurugu*. **A**, **B** Cleistospermia in the lumen of the deferent duct. **C**, **D**, **E**, **F** 3D surface reconstruction of a cleistosperm

**Spermatozoa.** Acrosomal complex. AV long and cylindrical, posteriorly slightly extends into the prcN, subacrosomal space extends throughout the entire AV (Figs. 25B and 26B). Subacrosomal space notably enlarged towards posterior (Fig. 25B). AF extends into the NC, ending approximately at half of prcN (Figs. 25B and 26E). Nucleus. Asymmetric, prcN roundish to cone-shaped with an anterior indentation, IF almost spherical, occupying about half of the prcN (Fig. 26A). Chromatin condensation dense and homogenous (Fig. 26A, B). peN notably long, twice the length of the prcN, and flat in cross section (Figs. 25B and 26F). NC runs laterally inside the periphery of the prcN (Fig. 25B) before shifting into a lateral projection in the peN (Fig. 25C).

**Sperm transfer form.** Spherical cleistospermia surrounded by a secretion sheath (Fig. 26A, B), peN coiled once inside the cleistosperm, enclosing the prcN (Fig. 26E, F), axoneme coiled four times, mostly around the nucleus (Fig. 26C). Mitochondria and amorphous material present in the cytoplasm.

**Notes on spermiogenesis.** In mid spermatids, the subacrosomal space begins to enlarge towards posterior (Fig. 25A), ultimately expanding to the extent observable in e.g. Figs. 25B and 26B. Late spermatids exhibit a prominent granular electron dense content within the IF (Fig. 25B, C), which is reduced to a homogenous matrix in mature stages (Fig. 26).

**Seminal secretions.** One type of secretion, round to oval, heterogenous with scattered electron dense granules (Fig. 4A).

### Arteminae | *Physocyclus globosus* (Taczanowski, 1874) (Figs. 27 and 28)

**Fig. 27:**
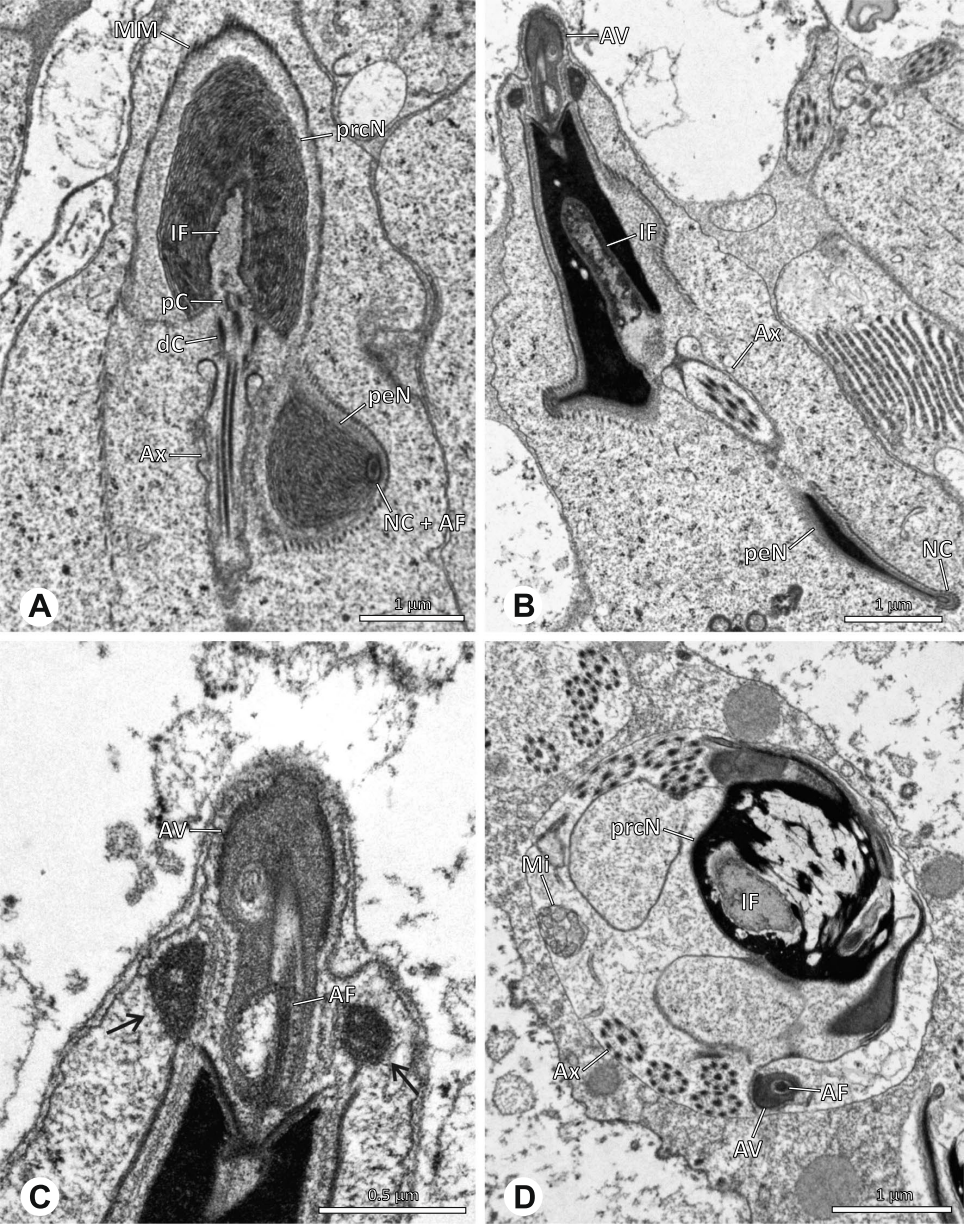
Spermiogenesis of *Physocyclus globosus*. TEM. **A** Early to mid spermatid. **B** Late spermatid. Note the dense chromatin condensation and the elongated peN. **C** Late spermatid, detail. The AV is slightly extending into the anterior pole of the nucleus and is flanked by electron dense “organizational centers” (arrows). **D** Coiled spermatid. Note that the chromatin condensation in this state is notably more heterogenous than in earlier stages of spermiogenesis

**Fig. 28:**
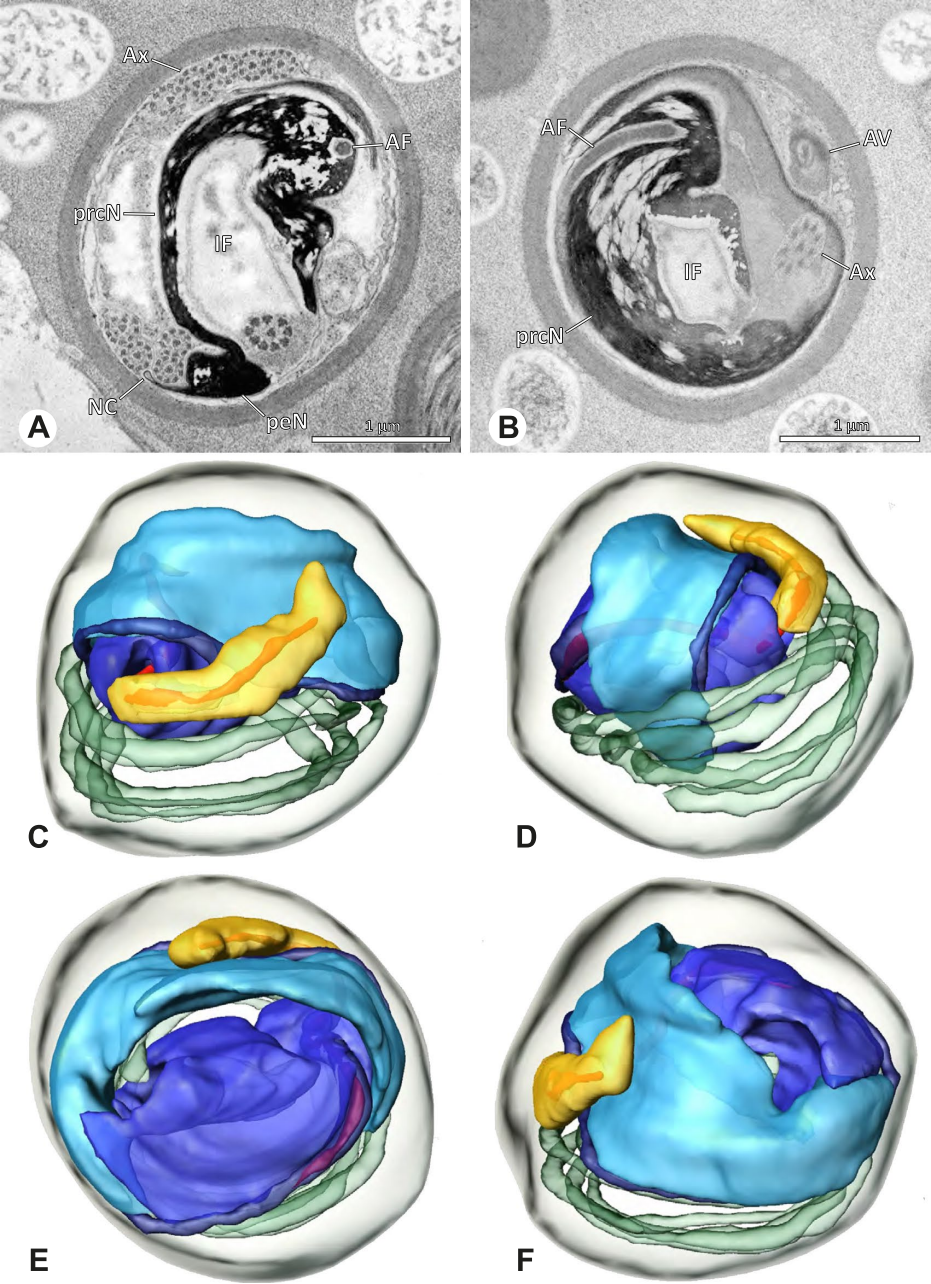
Ultrastructure and 3D surface reconstruction of cleistospermia of *Physocyclus globosus*. **A**, **B** Cleistospermia in the lumen of the deferent duct. Note the heterogenous chromatin condensation, the large IF, the stout AF and the position of the nuclear canal along the peN. **C**, **D**, **E**, **F** 3D surface reconstruction of a cleistosperm

**Spermatozoa. **Acrosomal complex. AV cylindrical, posteriorly slightly extends into the prcN with the subacrosomal space extends throughout the entire AV and widening towards posterior (Figs. 27C and 28C). AF extends into the NC, ending in the anterior half of the prcN (Fig. 28C, E). Nucleus. Asymmetric, prcN wide, with a deep IF (Fig. 28A). peN about twice as long as the prcN, flat and notably wide (Fig. 28F). Chromatin condensation heterogenous (Fig. 28B). NC runs laterally in the periphery of the prcN and shifts to a lateral projection in the peN (Figs. 27B and 28B).

**Sperm transfer form.** Spherical cleistospermia surrounded by a secretion sheath (Fig. 28A, B); peN coils once around the prcN (Fig. 28E); axoneme coiled four times laterally beside the nucleus (Fig. 28D). Acrosomal complex in major parts bent alongside the prcN and peN (Fig. 28 D). Cytoplasm heterogenous (Fig. 28B).

**Notes on spermiogenesis.** Late spermatids show a dense and homogenous chromatin condensation (Fig. 27B): During the coiling process, the condensation pattern becomes notably more heterogenous (Fig. 27D).

**Seminal secretions.** One type of secretion, round, electron lucent with heterogeneously scattered electron dense granules (Fig. 4B).

### Modisiminae | *Canaima?* sp. n. ‘Dup118’ (Fig. 29)

**Fig. 29:**
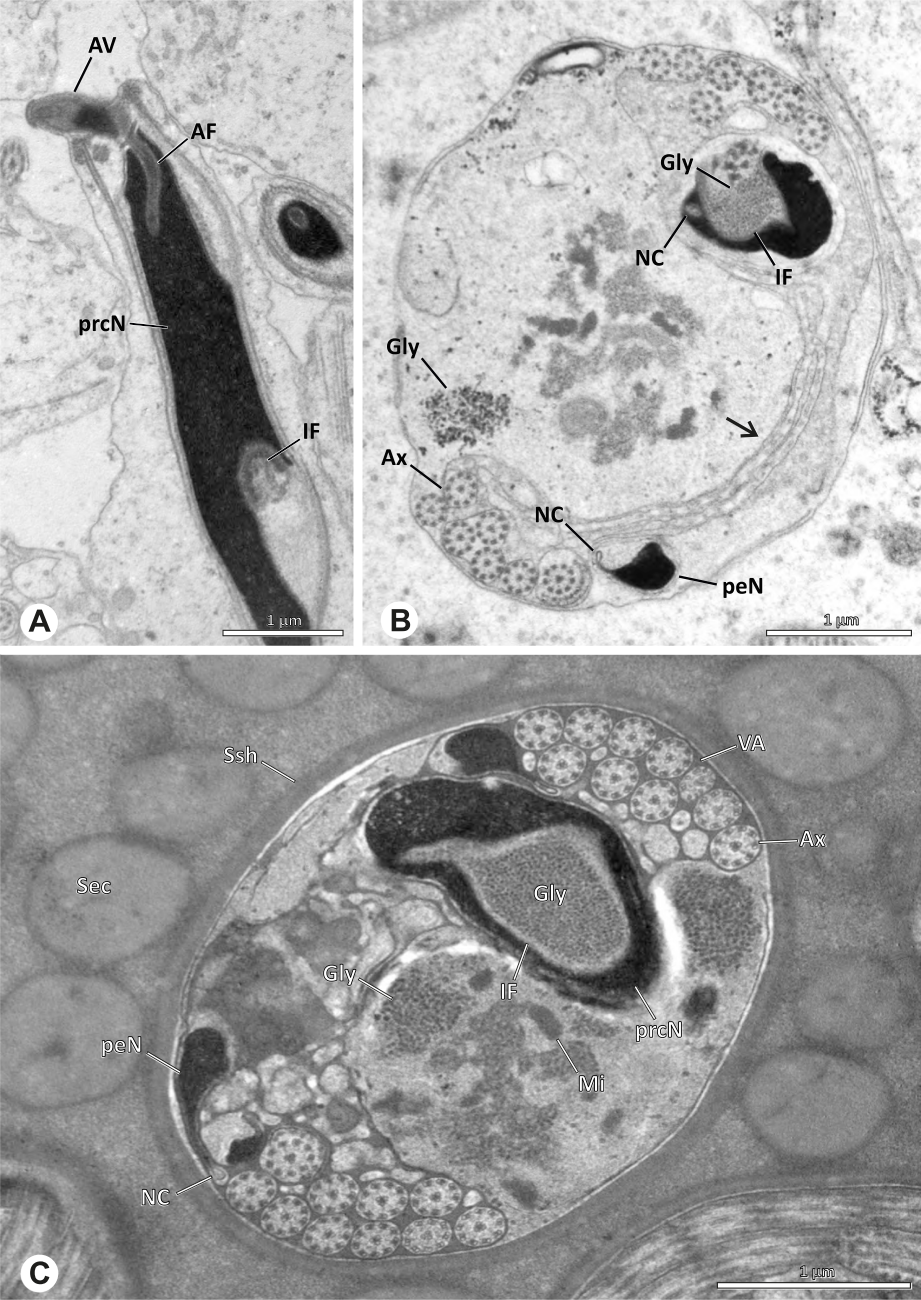
Spermiogenesis and cleistosperm of *Canaima?* sp*.* n. *‘*Dup118’ TEM. **A** Mid to late spermatid, longitudinal section. Note the angled shape of the AV. **B** Coiled spermatid. Note the stacks of membranes (arrow) and the free glycogen in the cytoplasm. **C** Cleistosperm. Note the vesicular area around the axoneme and the position of the nuclear canal along the peN

**Spermatozoa. **Acrosomal complex. AV cylindrical, subacrosomal space narrow, extends throughout the entire AV (Fig. 29A). AF stout, extends into the NC and ending before the axonemal basis. Nucleus. Asymmetric, prcN short. IF small, filled with glycogen (Fig. 29C), peN long and flat (Fig. 29C). NC runs in the periphery of the prcN and shifts to a thin lateral projection along the peN (Fig. 29C, D).

**Sperm transfer form.** Oval cleistospermia with a secretion sheath. Axoneme coiled ten times around the nucleus in two layers, cytoplasm heterogenous with free glycogen, membranous areas and mitochondria, prominent vesicular area present (Fig. 29C).

**Notes on spermiogenesis.** Late spermatids show a notably angled AV in relation to the prcN (Fig. 29A). In the initial phase of the coiling process within the testes, stacks of membranes are present in the cytoplasm of spermatozoa (Fig. 29B arrow) and ultimately form the vesicular area during the late phase of the coiling process.

**Seminal secretions.** One type of secretions; drop-shaped, heterogeneous with an electron dense margin and less dense center (Fig. 5M).

### Modisiminae | *Carapoia* spp. (Fig. 30)

**Fig. 30:**
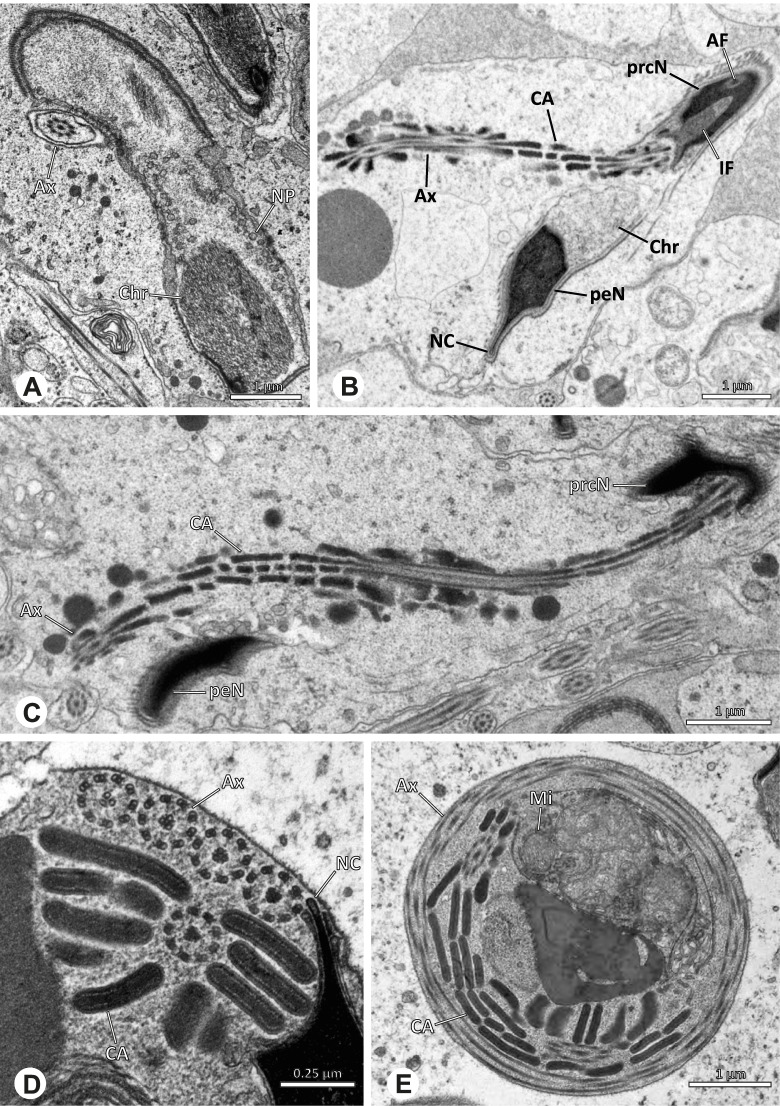
Spermiogenesis and cleistosperm of *Carapoia* spp. TEM. **A** *C. nairae*. Early spermatid, longitudinal section. Note the elongated peN. **B** *C. lutea.* Mid- to late spermatid. The heterogenous chromatin condensation as well as the forming CA are visible. **C** *C. nairae*. Late spermatid, longitudinal section. The peN is strongly elongated and the posterior centriolar adjunct material begins to develop. **D** *C. nairae*. Cross section through the axoneme of a coiled spermatid, showing the organization of the lamellae of the centriolar adjunct material. **E** *C. nairae*. Early cleistosperm in the intersection between testes and deferent duct. The secretion sheath is not developed yet

**Spermatozoa.** Acrosomal complex. AF ends clearly after the axonemal basis. Nucleus. Asymmetric, prcN short, IF short, filled with granular material in *Carapoia lutea* (Fig. 30B), peN long and broad, posterior centriolar adjunct material shaped as a short collar of layered lamellae (Fig. 30C, D, E), NC in prcN positioned laterally in the periphery of the nucleus, shifting to a projection along the peN. Chromatin condensation homogenous (Fig. 30C).

**Sperm transfer form.** Spherical cleistospermia surrounded by a secretion sheath. Axoneme coiled five times around the nucleus (Fig. 30D). Cytoplasm heterogenous with electron dense material, mitochondria present (Fig. 30E).

**Notes on spermiogenesis.** In early spermatids, the chromatin condensation starts in a globular pattern (Fig. 7B). During this stage, an initial fusion of at least two spermatids can be observed in *Carapoia nairae* (Fig. 9A). In mid to late spermatids, the chromatin is partly highly and homogenously condensed, while some peripheral areas of the nucleus still remain uncondensed (Fig. 30A, B). The lamellae-shaped centriolar adjunct material starts to form at late developmental stages (Fig. 30B, C). Spermatids ultimately separate, forming cleistospermia.

**Seminal secretions. ***Carapoia nairae*: One type of secretions, globular, electron lucent with scattered electron dense granules (Fig. 4F).

### Modisiminae | *Chibchea salta* Huber, 2000 (Figs. 31 and 32)

**Fig. 31:**
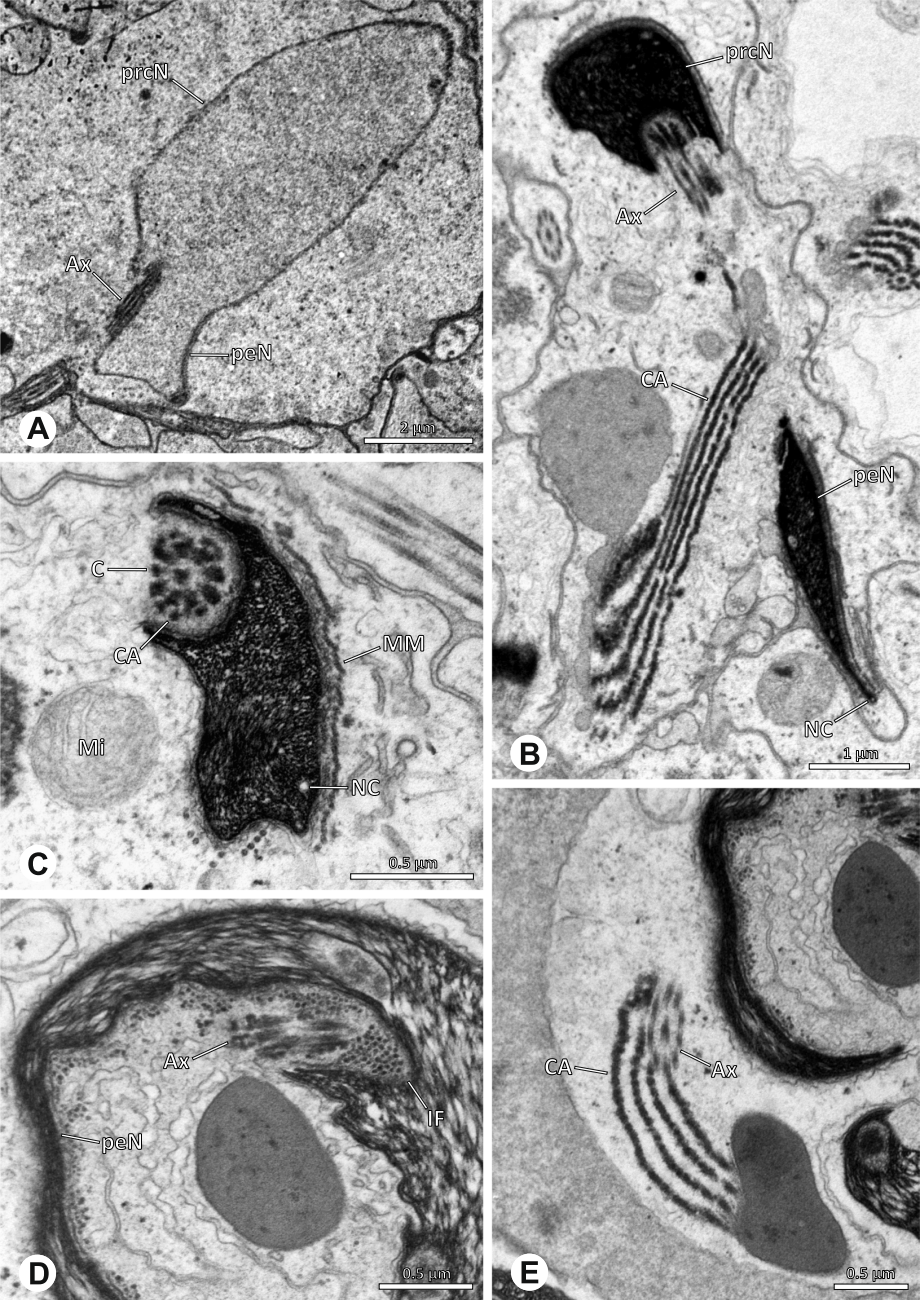
Spermiogenesis of *Chibchea salta*. TEM. **A** Early spermatid. **B**, **C** Mid to late spermatid. The centriolar adjunct material begins to develop. **D** Coiled spermatid. The IF is filled with glycogen in this stage. **E** Early cleistosperm. Note the beaded organization of the lamellae of the centriolar adjunct material

**Fig. 32:**
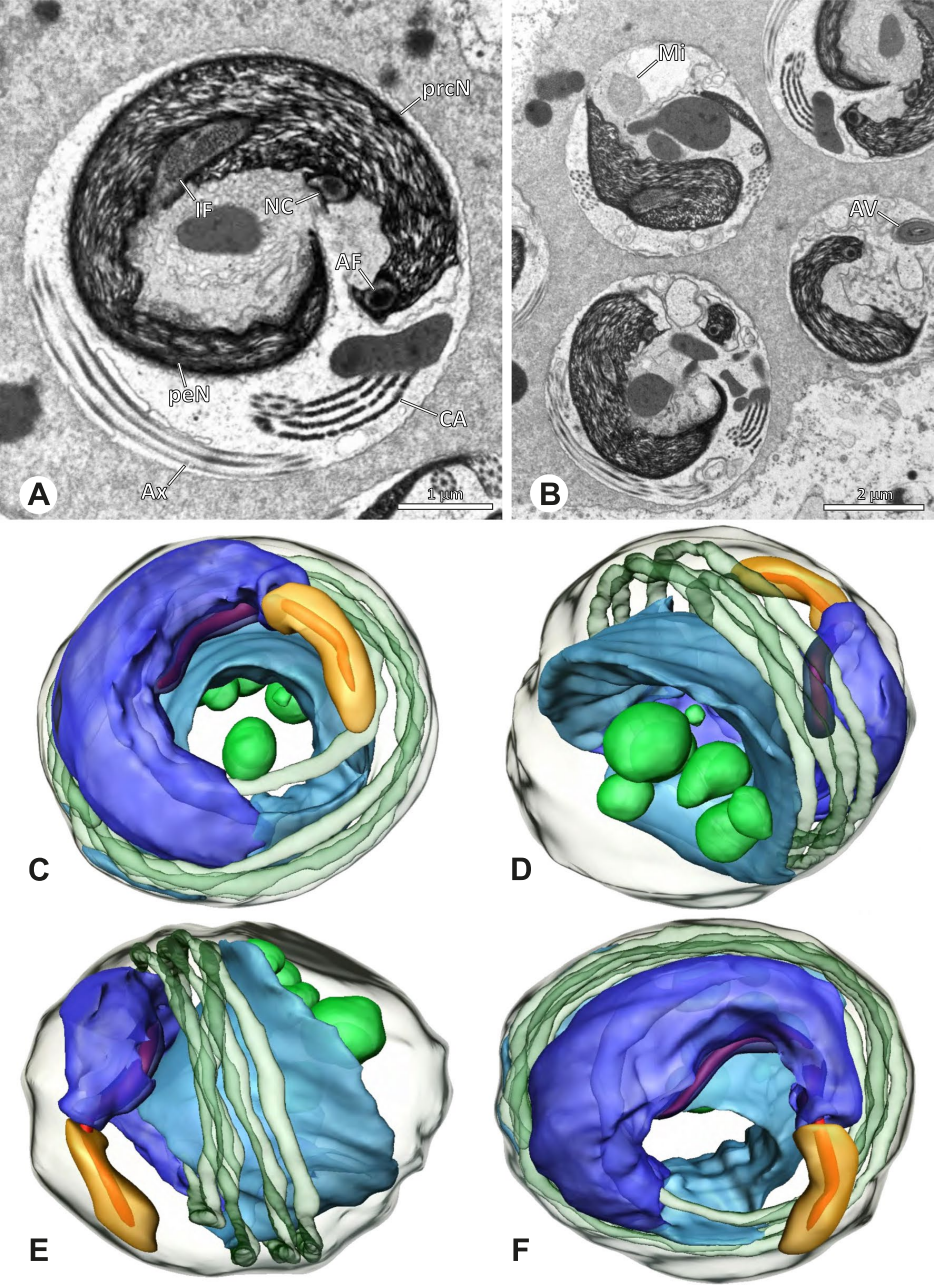
Ultrastructure and 3D surface reconstruction of the cleistosperm of *Chibchea salta*. Note that (**A**) and (**B**) depict early cleistospermia that do not possess a fully developed secretion sheath

**Spermatozoa.** Acrosomal complex. Short, cylindrical (Fig. 32C, F), subacrosomal space extends throughout the whole AV, giving rise to the stout AF (Fig. 32A). Nucleus. Asymmetric, prcN stout, NC wide and in the periphery of the nucleus (Fig. 32A, C), IF deep (Fig. 32F), filled with glycogen (Fig. 32A), peN longer than the prcN, flat and notably wide (Fig. 32E), posterior centriolar adjunct consisting of a collar of four beaded lamellae, projecting along the anterior part of the axoneme (Fig. 32A). Chromatin condensation homogenous.

**Sperm transfer form.** Spherical cleistospermia with a secretion sheath; AV and nucleus spirally coiled, axoneme centrally coiled three times around the nucleus (Fig. 32C, E). Cytoplasm electron lucent (Fig. 32A, B), mitochondria present.

**Notes on spermiogenesis. **Chromatin condensation in early spermatids starts in a heterogeneous pattern with scattered electron dense patches (Fig. 7C). At this stage, an initial fusion of at least two spermatids can be observed (Fig. 9B), which later separate. In early to mid-spermatids, the peN starts to transform to a characteristic elongated to broad shape (Fig. 31A). In late spermatids, the centriolar adjunct material begins to form as small lamellae (Fig. 31C), with the bead-like configuration being fully observable in late stages (Fig. 31B, E); IF filled with glycogen (Fig. 31D).

**Seminal secretions.** Two types of secretions: one small globular, homogenously electron dense; the other large globular, with an electron dense margin and an electron lucent center (Fig. 4G).

### Modisiminae | *Ciboneya antraia* Huber & Pérez, 2001 (Figs. 33 and 34)

**Fig. 33:**
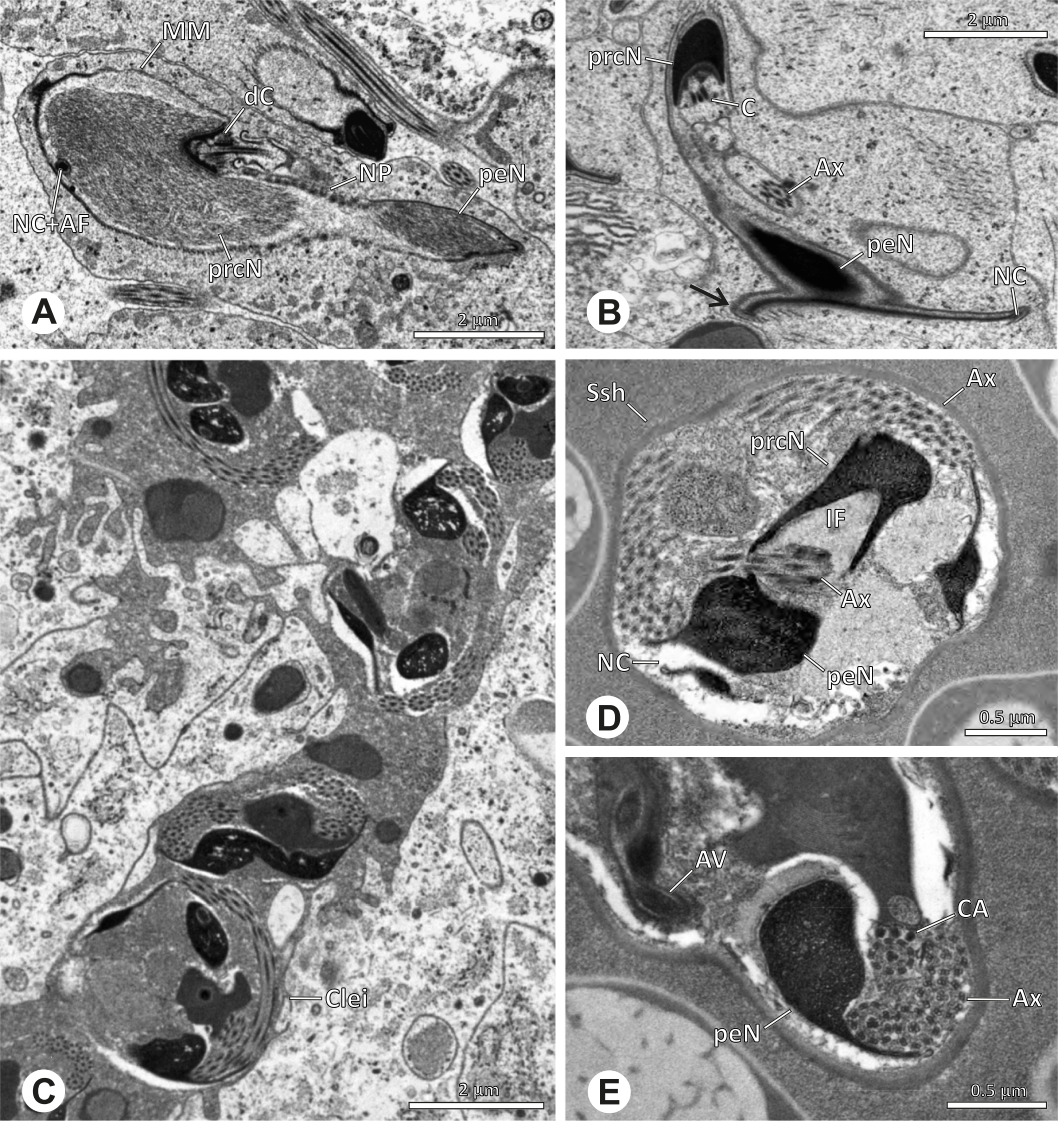
Spermiogenesis and cleistospermia of *Ciboneya antraia*. TEM. **A** Early to mid spermatid. Note the nuclear pores. **B** Late spermatid. Note the hook-like extension of the peN (arrow). **C** Coiled spermatids and early cleistospermia in the lumen of the distal part of the testis. **D** Cleistosperm with fully developed secretion sheath. **E** Cleistosperm, cross-section. The small streaks of electron dense centriolar adjunct material are visible around the anterior part of the axoneme

**Fig. 34:**
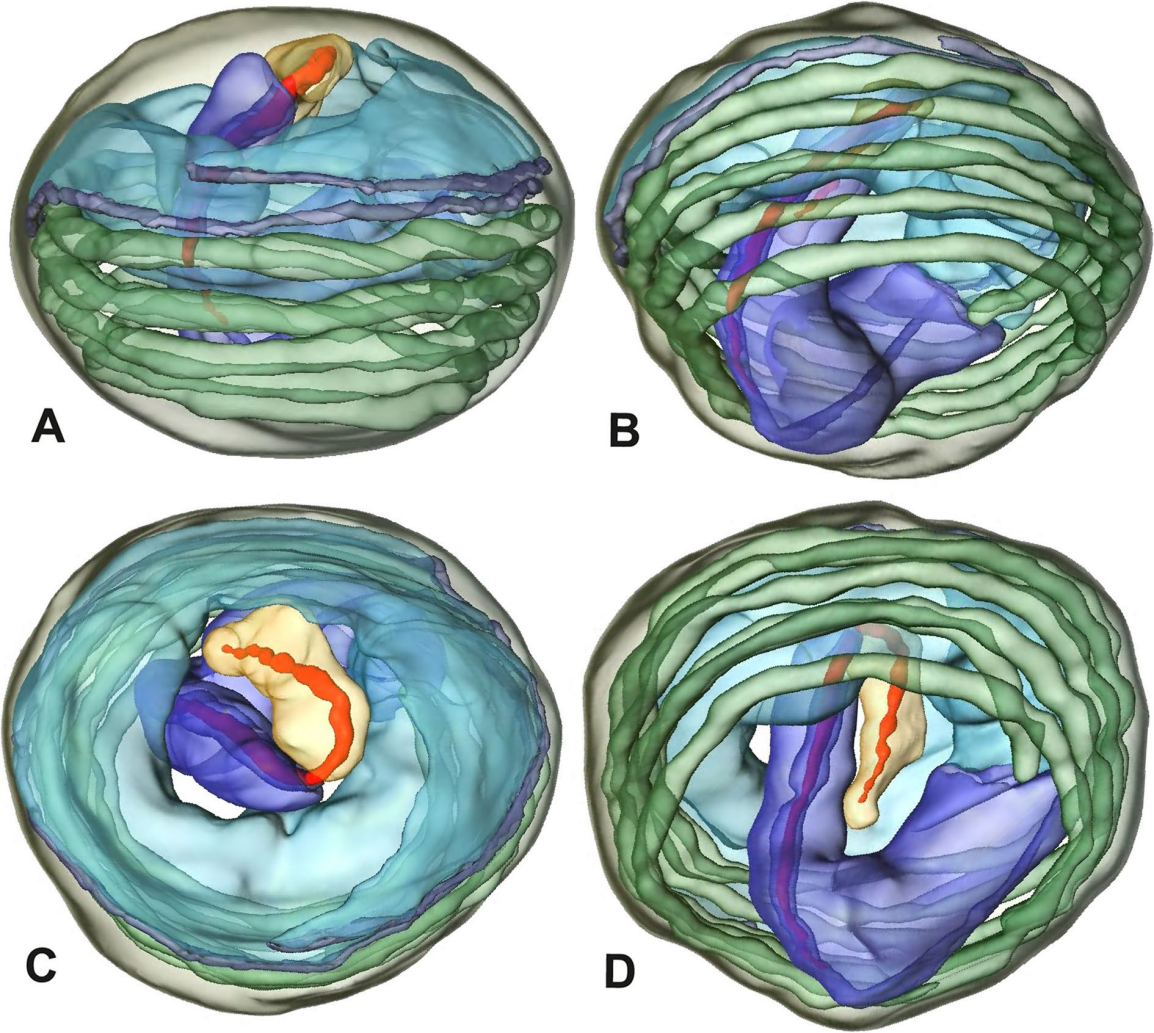
3D surface reconstruction of a cleistosperm of *Ciboneya antraia*

**Spermatozoa.** Acrosomal complex. AV stout and cylindrical, subacrosomal space extends throughout the entire AV. AF stout, extends through the anterior half of the prcN (Fig. 34). Nucleus. Asymmetric, prcN short, with a laterally situated NC (Figs. 33B and 34H). IF short (Fig. 33D). Beaded centriolar adjunct material inconspicuous, consisting of three electron dense streaks present alongside the most anterior part of the axoneme (Fig. 33E). peN voluminous (Fig. 34G). Chromatin condensation homogenous.

**Sperm transfer form. **Spherical cleistospermia with a secretion sheath (Fig. 33E), peN coiled around the prcN and the AV (Fig. 33E, G), axoneme long and coiled around the nucleus six times (Fig. 34F, H). Cytoplasm heterogenous with notably electron dense secretions (Fig. 33E); mitochondria present.

**Notes on spermiogenesis.** Early spermatids with a homogenous chromatin condensation, the peN starts to form as a small extension beside the stout prcN; the AV starts to form in a more roundish shape, the MM is tightly associated with the forming nucleus; IF short, comprising the centrioles. In mid to late spermatids, the peN starts to prolong extensively (Fig. 33A) and later shows a prominent hook-like extension (Fig. 33B arrow). In late spermatids, before the coiling process takes place, the centriolar adjunct material starts to form (Fig. 33B).

**Seminal secretions.** One type of secretion, spherical, heterogeneously electron lucent with electron dense granules, enclosed by membrane-like structures (Fig. 4H).

### Modisiminae | *Mecolaesthus* sp. n. ‘Ecu60’ (Fig. 35)

**Fig. 35:**
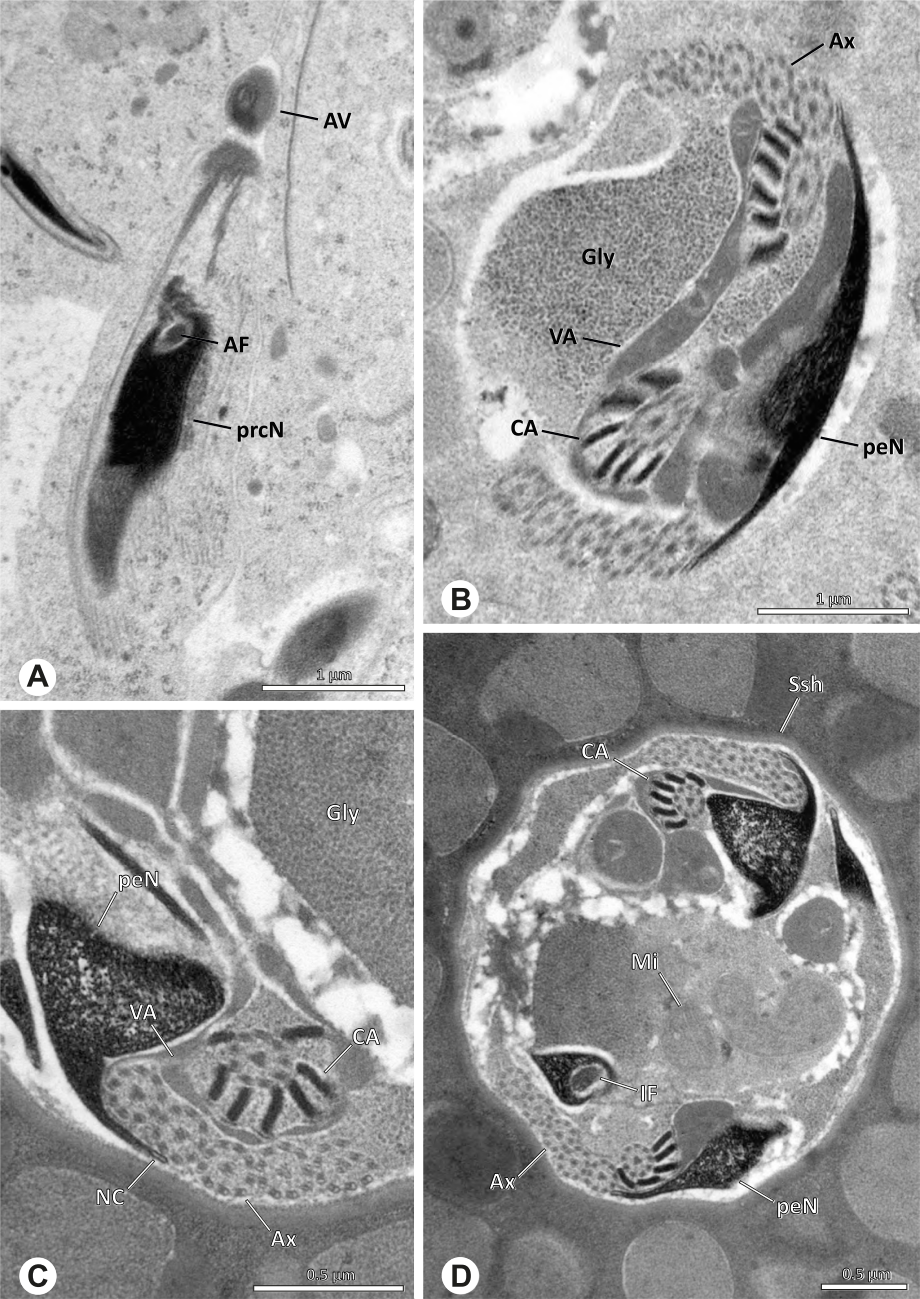
Spermiogenesis and cleistospermia of *Mecolaesthus* sp*.* n. *‘*Ecu60’ TEM. **A** Mid to late spermatid. Note the stout acrosomal filament. **B** Coiled late spermatid. Note the large accumulation of glycogen and the vesicular area around the anterior part of the axoneme as well as the lamellar centriolar adjunct material. **C** Detail of a cleistosperm, showing the peN with the nuclear canal in an extension, the vesicular area, the lamellar centriolar adjunct material and the free glycogen within the cytoplasm. **D** Cleistosperm in the lumen of the deferent duct

**Spermatozoa.** Acrosomal complex. AV cylindrical, subacrosomal space narrow. AF stout, extends into the nuclear canal and ends before the axonemal basis (Fig. 35A). Nucleus. prcN cylindrical, IF extends into about half of the prcN and is filled with electron dense material (Fig. 35D). peN long and flattened in cross-section (Fig. 35B). NC runs in the periphery of the prcN and shifts into a crest-like projection along the peN (Fig. 35B, C). Posterior centriolar adjunct material present, shaped as a collar of six layered lamellae around the anteriormost portion of the axoneme (Fig. 35B, C).

**Sperm transfer form.** Roundish cleistospermia surrounded by a secretion sheath, axoneme coiled six times beside the nucleus, cytoplasm heterogenous with accumulations of free glycogen and mitochondria, vesicular area around parts of the axoneme and nucleus (Fig. 35C).

**Notes on spermiogenesis.** The centriolar adjunct material as well as the vesicular area only begin to form during the latest stages of spermatid development or at the very beginning of the coiling process (Fig. 35B). During coiling, large amounts of glycogen begin to accumulate in the cytoplasm (Fig. 35B).

**Seminal secretions.** One type of seminal secretions, roundish and homogenous (Fig. 5N).

### Modisiminae | *Mesabolivar* spp. (Figs. 36 and 37)

**Fig. 36:**
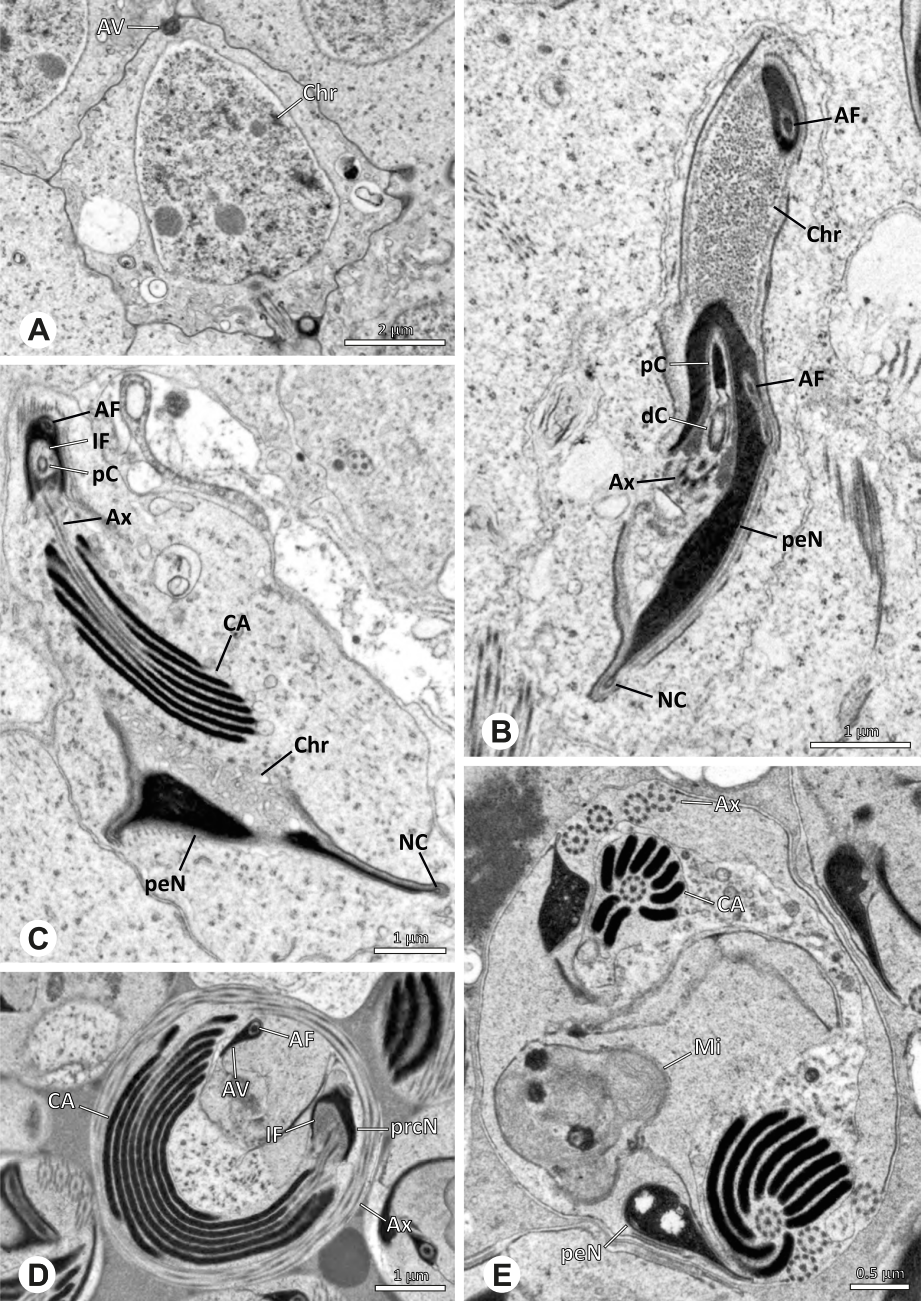
Spermiogenesis and cleistospermia of *Mesabolivar* spp. TEM. **A** Early spermatid of *M. iguazu*, with a globular chromatin condensation pattern. **B** Mid to late spermatid of *M. cyaneotaeniatus*. Note the heterogenous chromatin condensation within the nucleus as well as the electron dense material in the direct surroundings of the proximal centriole. **C** Late spermatid of *M. iguazu*. The centriolar adjunct material consists of short lamellae, which begin to develop at this stage. **D** Early cleistosperm of *M. iguazu*, showing the long, layered lamellae of the centriolar adjunct material along the anterior part of the axoneme. **E** Coiled spermatid of *M. iguazu*. Note that the number of lamellae of the centriolar adjunct material correspond with the amount of microtubule doublets of the axoneme

**Fig. 37:**
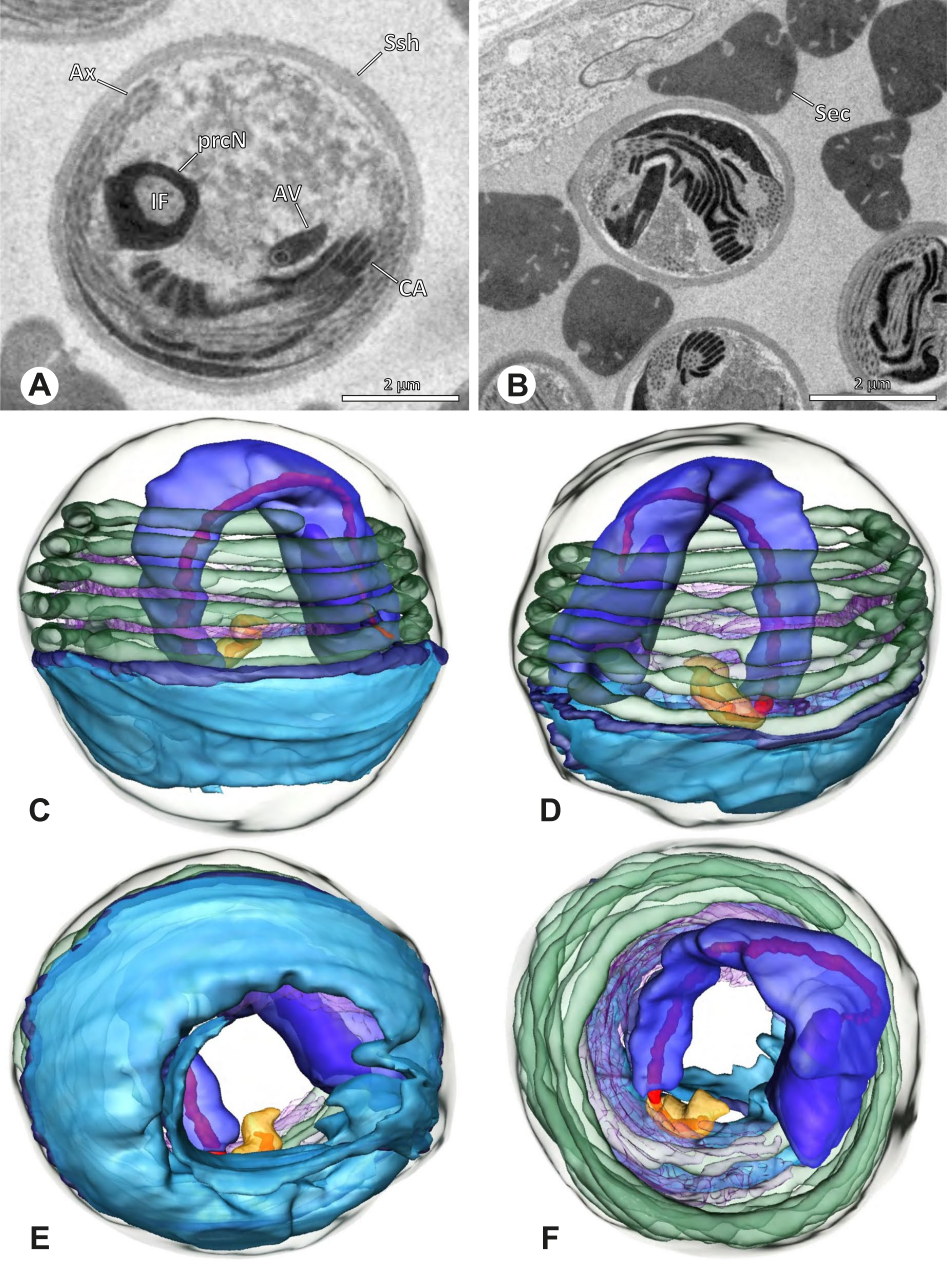
Ultrastructure and 3D surface reconstruction a cleistosperm of *Mesabolivar cyaneotaeniatus*, representing the general configuration of cleistospermia for the studied representatives of the genus

**Spermatozoa.** Acrosomal complex. AV cylindrical, subacrosomal space extends throughout the whole AV. AF stout, extends into the NC as far as the region of the axonemal basis (Fig. 36B). Nucleus. Asymmetric, prcN cylindrical, with the NC runs in the periphery (Figs. 37C and 36B, C). Within the peN, the NC is situated in a thin lateral projection (Fig. 36C, E). IF short, filled with granular material (Fig. 36C, D). Lamellar centriolar adjunct material present, forming a layered collar around the axoneme and projecting along approximately one third to half of it (Figs. 36C, D and 37A, B). The number of lamellae correlates with the nine outer doublets of the Ax (Fig. 36E). peN flat to triangular in cross section (Figs. 36E and 37C, E).

**Sperm transfer form.** Spherical cleistospermia with a secretion sheath (Fig. 37A, B). prcN bent and surrounded by the coiled peN (Fig. 37D). Ax coiled five times around the nucleus (Fig. 37C, D). Cytoplasm heterogenous with electron dense granula (Fig. 37A). Mitochondria present.

**Notes on spermiogenesis.** Chromatin condensation in early spermatids globular (Fig. 36A). Mid to late spermatids show a partly dense and homogenous chromatin condensation, while in parts of the nucleus the chromatin is nearly uncondensed and appears granular (Fig. 36B). The characteristic lamellae of the centriolar adjunct material are only developed in late spermatids before the coiling process (Fig. 36C, D). Already in early developmental stages, an initial fusion of several spermatids is observable (Fig. 9C, D), which prevails until late stages before ultimately forming cleistospermia.

**Seminal secretions.** *Mesabolivar cyaneotaeniatus:* one type of secretion, irregularly shaped, electron dense with small electron lucent inclusions (Fig. 4). No data available for other studied species.

### Modisiminae | *Modisimus elongatus* Bryant, 1940 (Figs. 38 and 39)

**Fig. 38:**
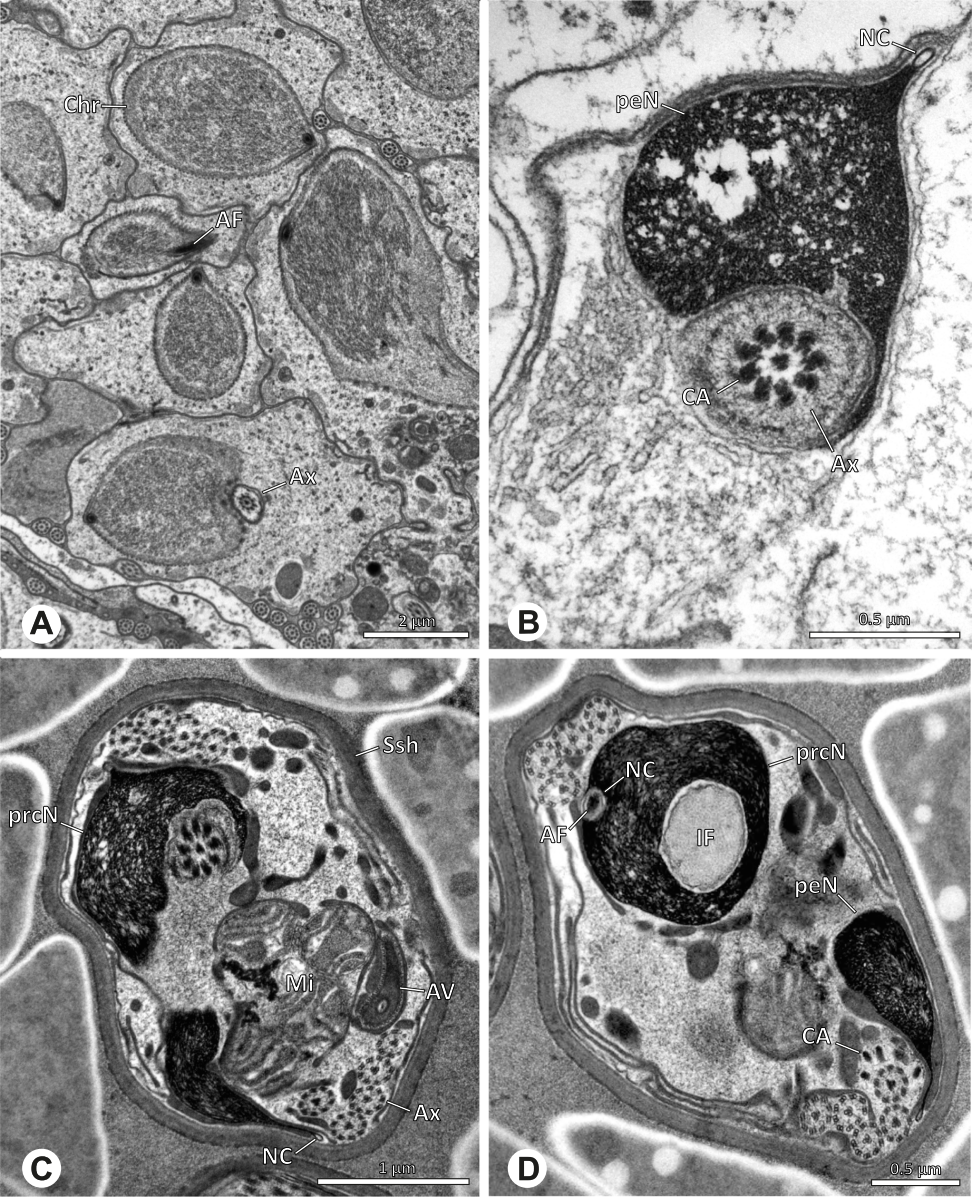
Spermiogenesis and cleistospermia of *Modisimus elongatus*. TEM. **A** Early spermatids, showing a heterogenous condensation pattern. **B** Late spermatid, cross-section. The centriolar adjunct material begins to develop at this stage, as visible by the peculiar electron-dense material surrounding the axoneme. **C** Cleistosperm. Note the heterogenous cytoplasm with the prominent aggregation of mitochondria. **D** Cleistosperm. The centriolar adjunct material is visible as well as the position of the nuclear canal in a projection along the peN

**Fig. 39:**
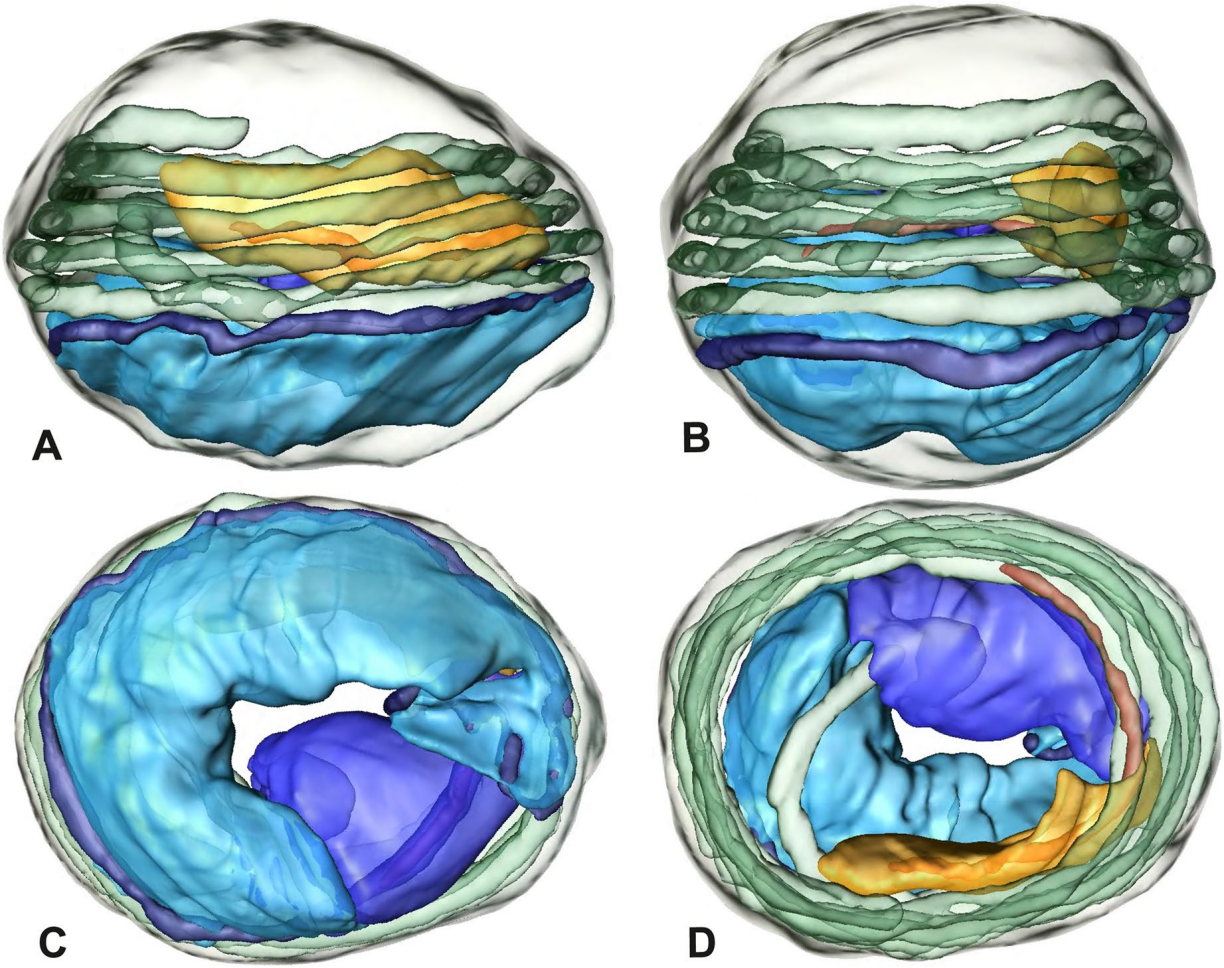
3D surface reconstruction of a cleistosperm of *Modisimus elongatus*

**Spermatozoa.** Acrosomal complex. AV wide, subacrosomal space extends through the entire AV, AF stout. (Fig. 39A). Nucleus: asymmetric, prcN compact to cone-shaped, NC in lateral position along the prcN, shifting to a projection along the peN (Fig. 38C, D). IF short (Fig. 39D) and containing homogenous material (Fig. 38D). Collar of beaded filamentous centriolar adjunct in the most anterior part of the axoneme (Fig. 38D). peN nearly twice as long as the prcN and notably voluminous, round to oval in cross-section (Figs. 38D and 39C).

**Sperm transfer form.** Spherical cleistospermia surrounded by a secretion sheath (Fig. 38C, D). prcN and peN compactly coiled (Fig. 39C), AV resting on top of the peN (Fig. 39A, D). Ax coiled five times beside the nucleus (Fig. 39A, B). Cytoplasm heterogenous with electron dense granules and secretion; mitochondria present (Fig. 38C). Vesicular area present around restricted parts of the Ax (Fig. 38D).

**Notes on spermiogenesis.** The development of the centriolar adjunct material could only be observed in late spermatids (Fig. 38B).

**Seminal secretions.** One type of secretion, shape irregular to triangular, electron dense center with electron lucent margin and scattered electron lucent spots (Fig. 4J).

### Modisiminae | *Otavaloa* cf. *piro *(Fig. 40)

**Fig. 40:**
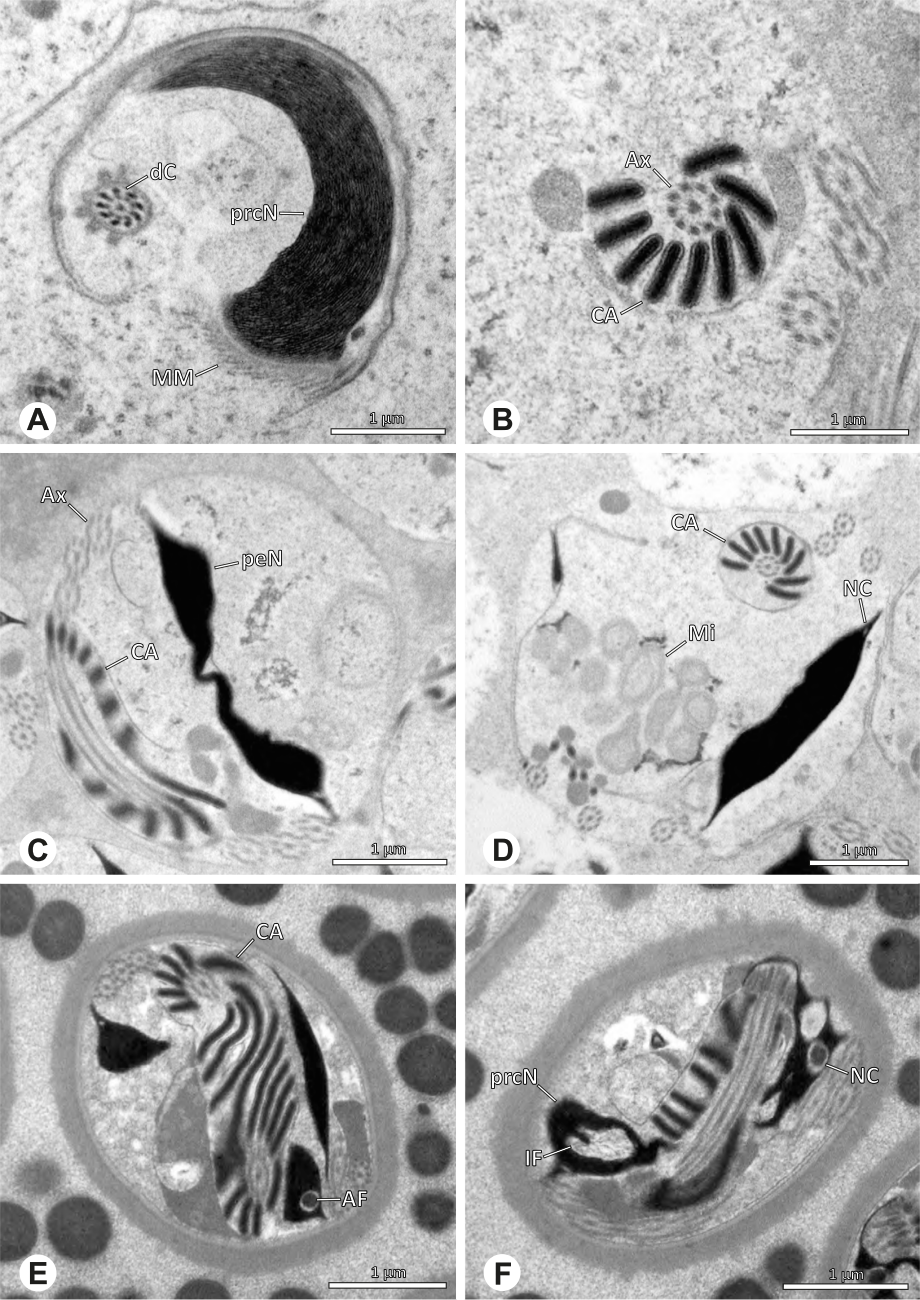
Spermiogenesis and cleistosperm of *Otavaloa* cf. *piro*. TEM. **A** Late spermatid. **B** Fully developed centriolar adjunct material. The number of lamellae corresponds to the number of microtubule doublets. **C**, **D** Early cleistospermia. Note the shape of the peN and the position of the nuclear canal. **E**, **F** Cleistospermia in the lumen of the deferent ducts. The secretion sheath is fully developed and the organization of the centriolar adjunct material becomes apparent. Note the stout acrosomal filament

**Spermatozoa.** Acrosomal complex. AF stout (Fig. 40E, F). Nucleus. Asymmetric, prcN slender to conical (Fig. 40A, F). NC wide in its anterior portion, becoming very narrow in its further course and situated in a pointed, triangular portion of the nucleus (Fig. 40D). peN flattened (Fig. 40C). IF filled with granular material. Centriolar adjunct material shaped as a collar of nine large electron-dense lamellae around the anterior part of the axoneme (Fig. 40E).

**Sperm transfer form.** Spherical to oval cleistospermia, surrounded by a secretion sheath (Fig. 40E, F). peN coiled around the prcN, axoneme coiled four times beside the nucleus (Fig. 40E, F). Cytoplasm heterogenous, with fine granula; mitochondria present (Fig. 40D, E, F).

**Notes on spermiogenesis.** The lamellated centriolar adjunct starts to form only in late spermatids (Fig. 40A).

**Seminal secretions.** One type of secretion, round, homogenously electron-dense.

### Modisiminae | *Priscula* sp. n. ‘Ecu93’ (Fig. 41)

**Fig. 41:**
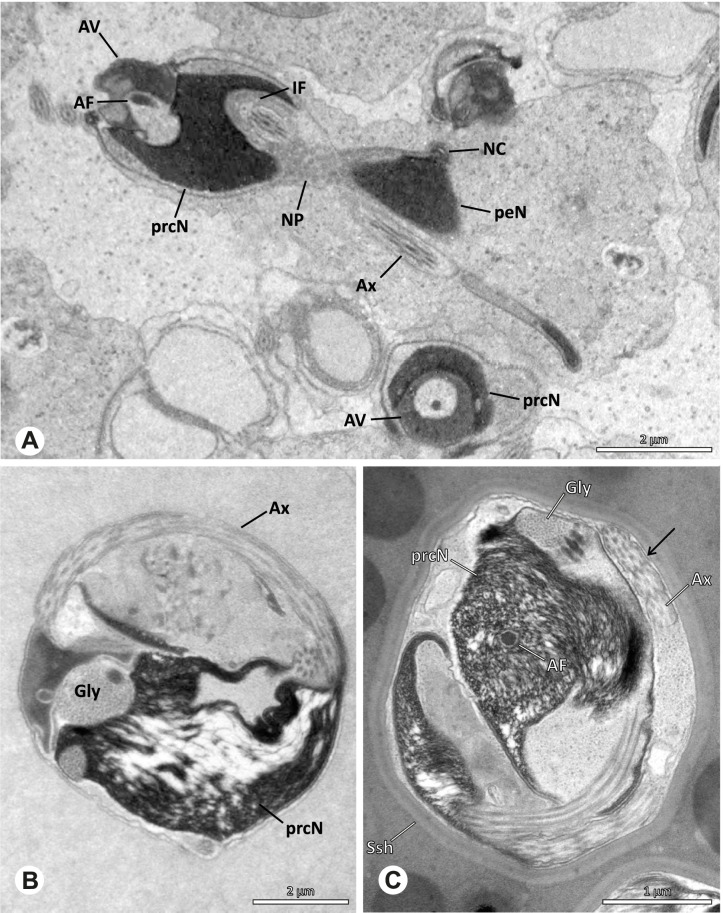
Spermiogenesis and cleistospermia of *Priscula* sp. n. ‘Ecu93’*.* TEM. **A** Late spermatid, longitudinal section. Note the irregular shape of the AV and the wide subacrosomal space as well as the elongated peN. **B** Early Cleistosperm in the distal lumen of the testis. Free glycogen is visible in the cytoplasm. **C** Cleistosperm in the lumen of the deferent duct. Note the stout AF and the membranous area around parts of the axoneme (arrow)

**Spermatozoa.** Acrosomal complex. AV cylindrical to irregularly shaped, subacrosomal space wide (Figs. 7A and 41A), extends through the entire AV. AF stout, extends as far as to the region of the axonemal basis (Fig. 41C). Nucleus. Asymmetric, prcN short. IF deep and filled with glycogen, peN long and flat (Fig. 41A). The NC runs in the periphery of the prcN and peN (Fig. 41A). Chromatin condensation homogenous. Axoneme. Axonemal basis close to the AV (Fig. 41A).

**Sperm transfer form.** Spherical to oval cleistospermia with a secretion sheath. Ax coiled four times beside the nucleus. Cytoplasm heterogenous with free glycogen, mitochondria and membranous areas. Membranous area present around parts of the axoneme (Fig. 41C arrow).

**Notes on spermiogenesis.** During the coiling process, large accumulations of glycogen as well as developing membranous areas can be observed in the cytoplasm of the spermatozoa (Fig. 41B).

**Seminal secretion.** One type of secretion, round and drop-shaped, homogenously electron dense (Fig. 5O).

### Modisiminae | *Tupigea teresopolis* Huber, 2000 (Figs. 42 and 43)

**Fig. 42:**
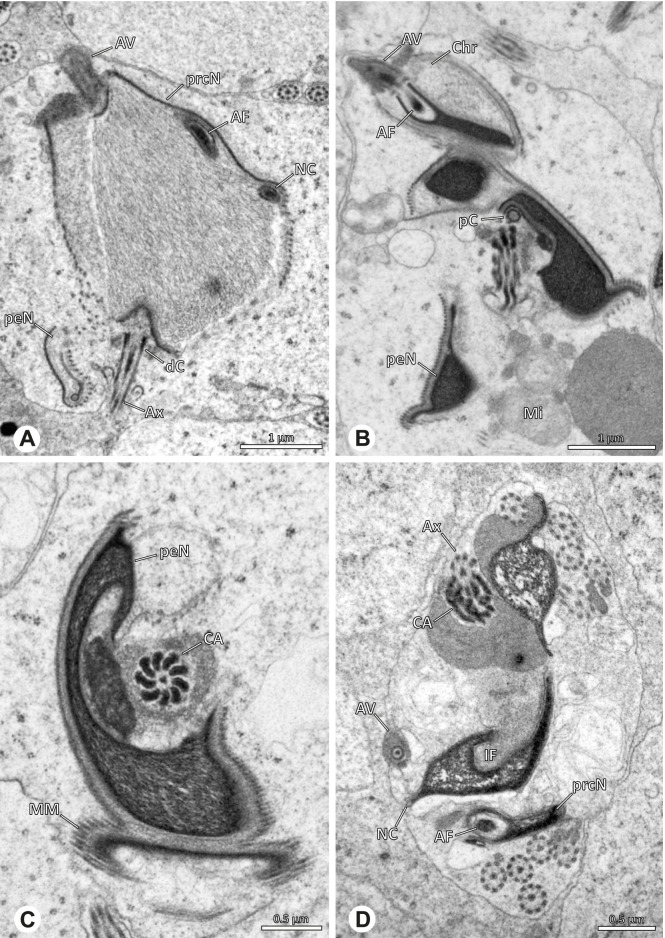
Spermiogenesis of *Tupigea teresopolis*. TEM. **A** Early to mid-spermatid. Note the peripheral position of the NC and the heterogenous condensation of chromatin towards the margin of the nucleus. **B** Late spermatid. Note the heterogenous chromatin condensation as well as the pC which is positioned perpendicular to the dC. **C** Late spermatid. The centriolar adjunct material begins to form. **D** Coiled spermatid. The centriolar adjunct material is developed as beaded lamellae around the anterior part of the axoneme

**Fig. 43:**
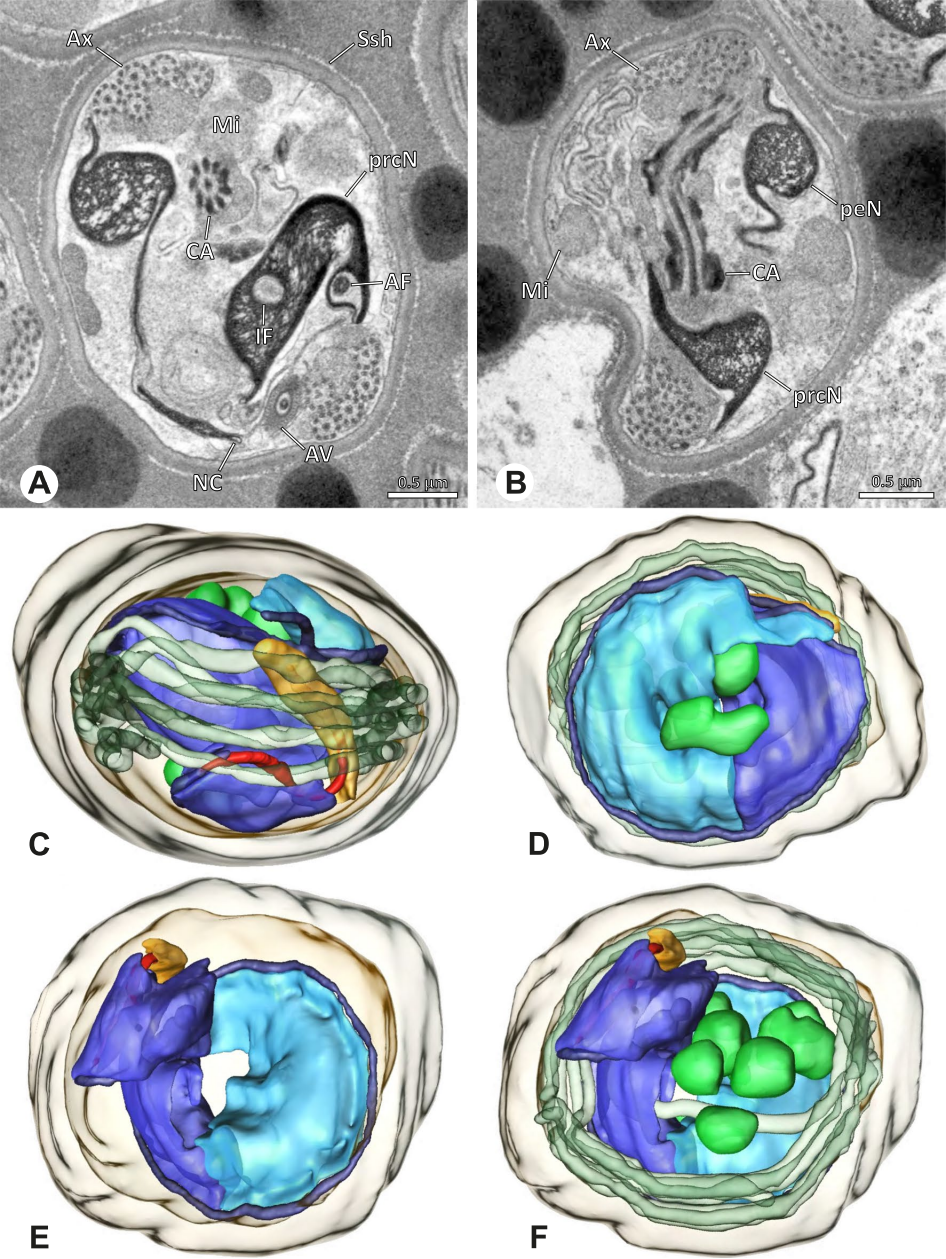
Ultrastructure and 3D surface reconstruction of the cleistosperm of *Tupigea teresopolis*. **A**, **B** Cleistospermia in the lumen of the deferent duct. Note the stout AF and the position of the NC in the prominent extension of the peN. **C**, **D**, **E**, **F** 3D surface reconstruction of a cleistosperm

**Spermatozoa.** Acrosomal complex. AV cylindrical, subacrosomal space extends throughout the whole AV, giving rise to the stout AF (Fig. 43C). Nucleus. Asymmetric, prcN stout and bending nearly perpendicular against itself (Fig. 43A, E), NC in lateral position throughout the prcN, situated inside a prominent lateral crest along the peN (Fig. 43A, B, E). IF deep. Centriolar adjunct consists of small beaded lamellae projecting along the most anterior part of the axoneme (Fig. 43B). peN round to oval and stout (Fig. 43B). Chromatin condensation heterogenous throughout the whole nucleus with less densely condensed areas (Fig. 43A).

**Sperm transfer form.** Spherical cleistospermia surrounded by a secretion sheath (Fig. 43A, B). prcN and peN bent alongside each other (Fig. 43E). AV bent beside the prcN (Fig. 43C). Ax coiled five times around the nucleus (Fig. 43C, F). Cytoplasm heterogenous, mitochondria present (Fig. 43A, B).

**Notes on spermiogenesis.** In mid and late spermatids, a pattern of irregularly condensed chromatin in the marginal areas of the nucleus is present (Fig. 42A, B). The proximal centriole is arranged perpendicular towards the distal centriole (Fig. 42B), giving the prcN a twisted to angled appearance. The centriolar adjunct material starts to form in late spermatid stages (Fig. 42B, C, D) before the coiling process; the centriolar adjunct material appears like beaded short lamellae (Fig. 42C).

**Seminal secretions.** One type of secretion, roundish, homogenously electron dense (Fig. 4K).

### Smeringopinae | *Smeringopina bineti* (Millot, 1941) (Figs. 44 and 45)

**Fig. 44:**
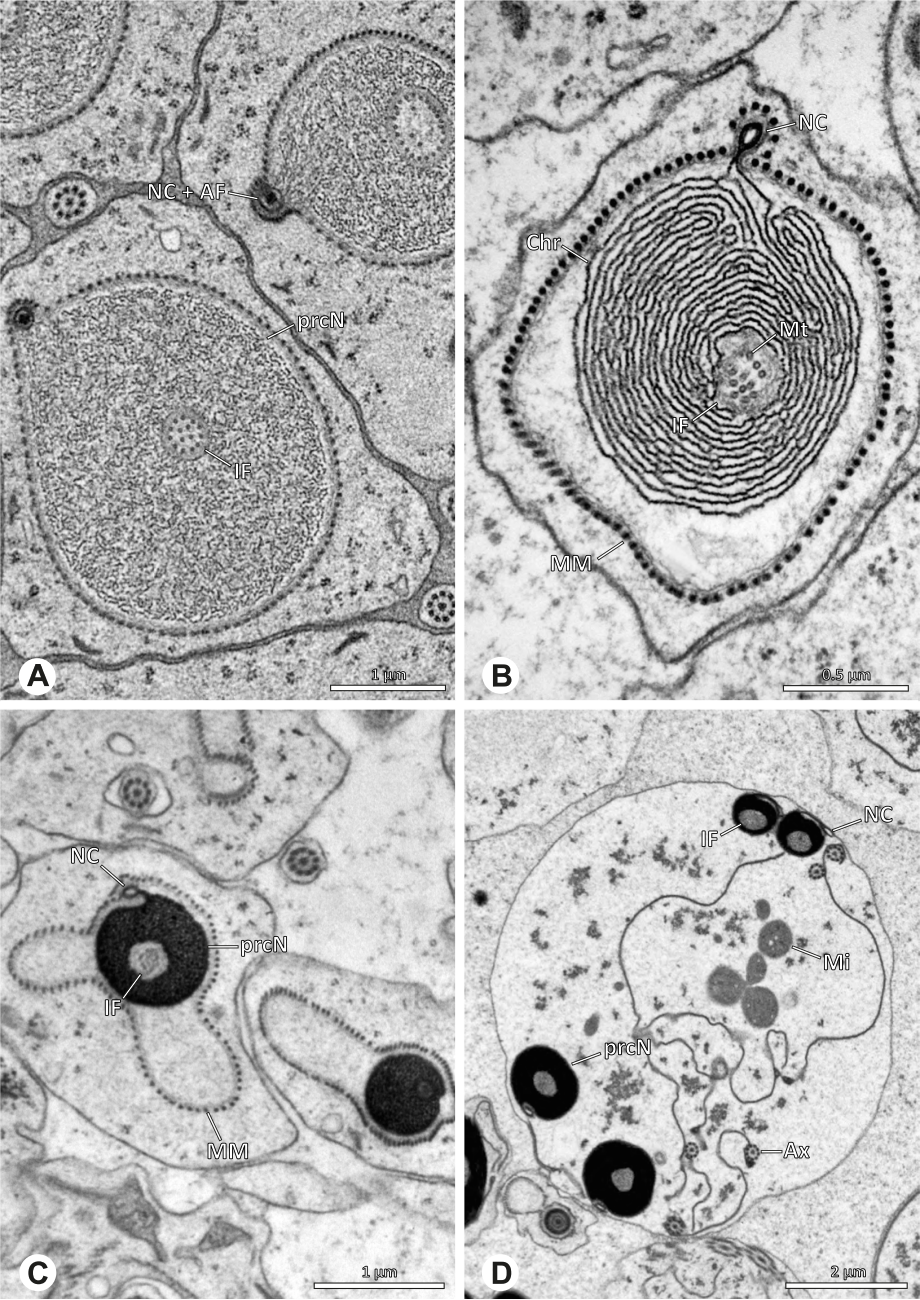
Spermiogenesis of *Smeringopina bineti*. TEM. **A** Early to mid-spermatid, cross-section. The chromatin condensation becomes filiform. **B** Mid spermatid, cross-section. Note the fibrillar chromatin condensation and the presence of microtubuli in the IF. **C** Late spermatid, cross-section. The microtubules in the IF begin to disintegrate. The NC is situated in a fine extension along the prcN. **D** Coiled spermatid in the lumen of the testis. The IF begins to fill with glycogen. The NC in its extension is folded towards the nucleus

**Fig. 45:**
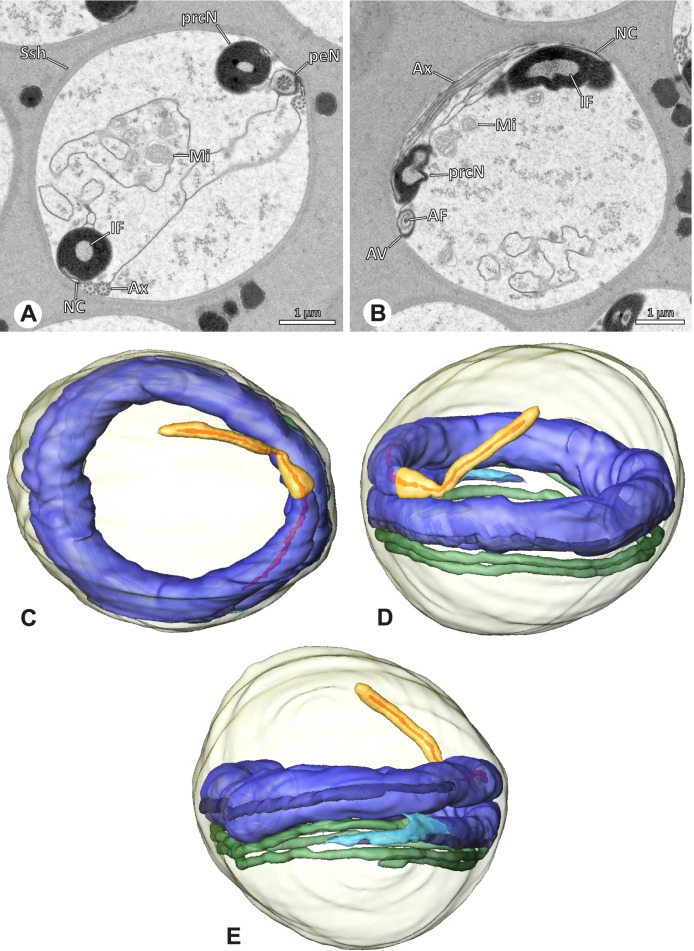
Ultrastructure and 3D surface reconstruction of cleistospermia of *Smeringopina bineti*. **A**, **B** Cleistospermia in the lumen of the deferent duct. The IF is filled with glycogen. Note the position of the NC folded towards the prcN as well as the short peN. **C**, **D**, **E** 3D surface reconstruction of a cleistosperm

**Spermatozoa.** Acrosomal complex. Av slender and cylindrical (Fig. 45C, D), subacrosomal space extends through the entire AV. AF thin, extends into the NC only to its anteriormost part (Fig. 45C). Nucleus. Asymmetric, prcN tubular, the NC projects centrally through its anterior part before shifting to a small lateral projection, which is bent alongside the prcN in the coiled state (Fig. 45A). IF deep, partly filled with glycogen (Fig. 45A, B). peN short and flat (Fig. 45E). Chromatin condensation homogenous.

**Sperm transfer form.** Spherical cleistospermia surrounded by a secretion sheath (Fig. 45A, B). Nucleus and axoneme spirally coiled, axoneme coils three times (Fig. 45E). Acrosomal complex bent on top of the prcN (Fig. 45D). Cytoplasm homogenous, mitochondria present (Fig. 45B).

**Notes on spermiogenesis.** Chromatin condensation starts in a homogenous pattern and becomes fibrillar in mid spermatids (Fig. 44A, B). IF filled with microtubules, which disappear in late spermatids before the coiling process takes place (Fig. 44B).

**Seminal secretions.** One type of secretion, irregularly granular, homogenously electron dense (Fig. 4D).

### Smeringopinae | *Smeringopus cylindrogaster* (Simon, 1907) (Figs. 46 and 47)

**Fig. 46:**
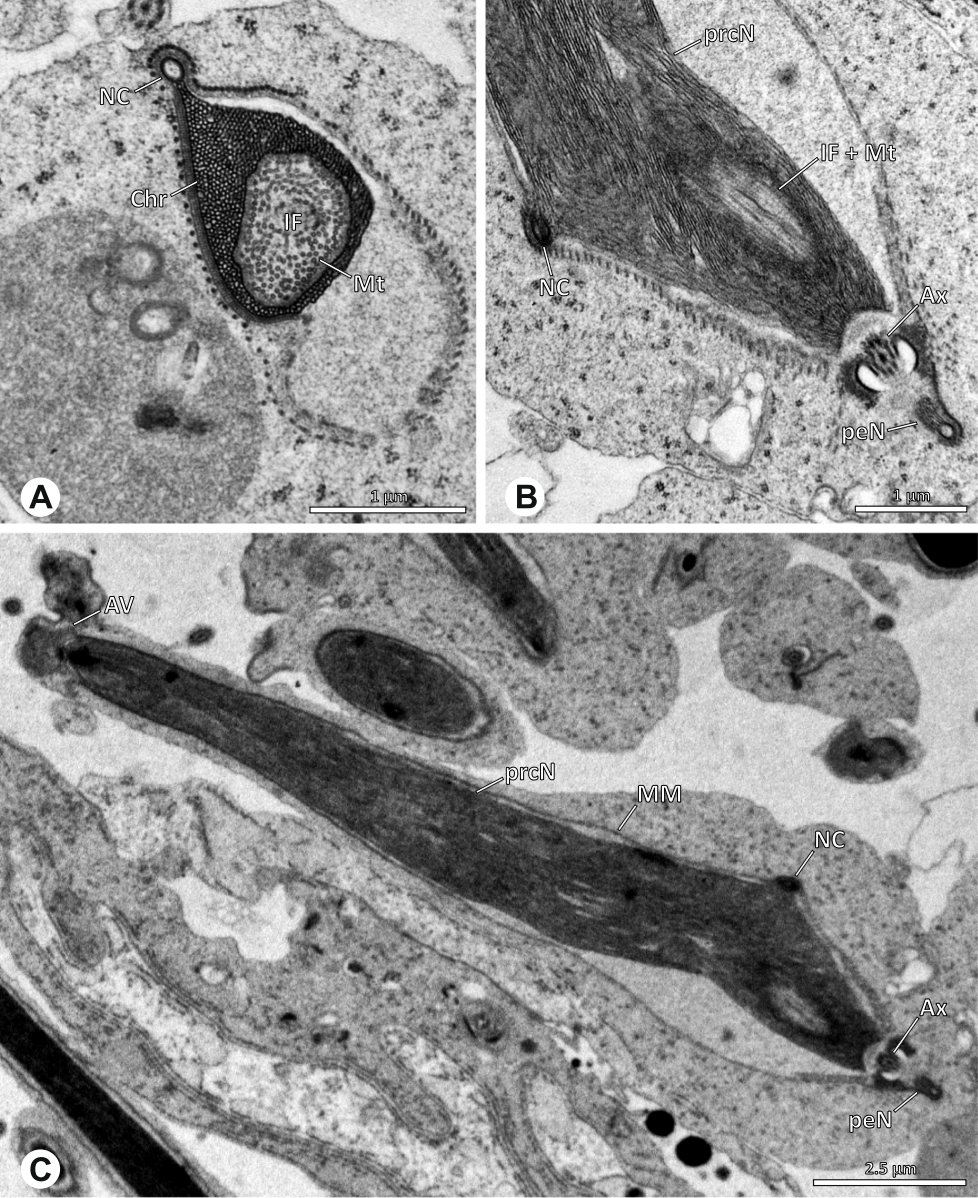
Spermiogenesis of *Smeringopus cylindrogaster*. TEM. **A** Mid to late spermatid, cross-section. The IF is filled with microtubules. **B** Late spermatid. Note that microtubules are still present inside the IF. **C** Late spermatid, longitudinal section. Note the roundish appearance of the AV

**Fig. 47:**
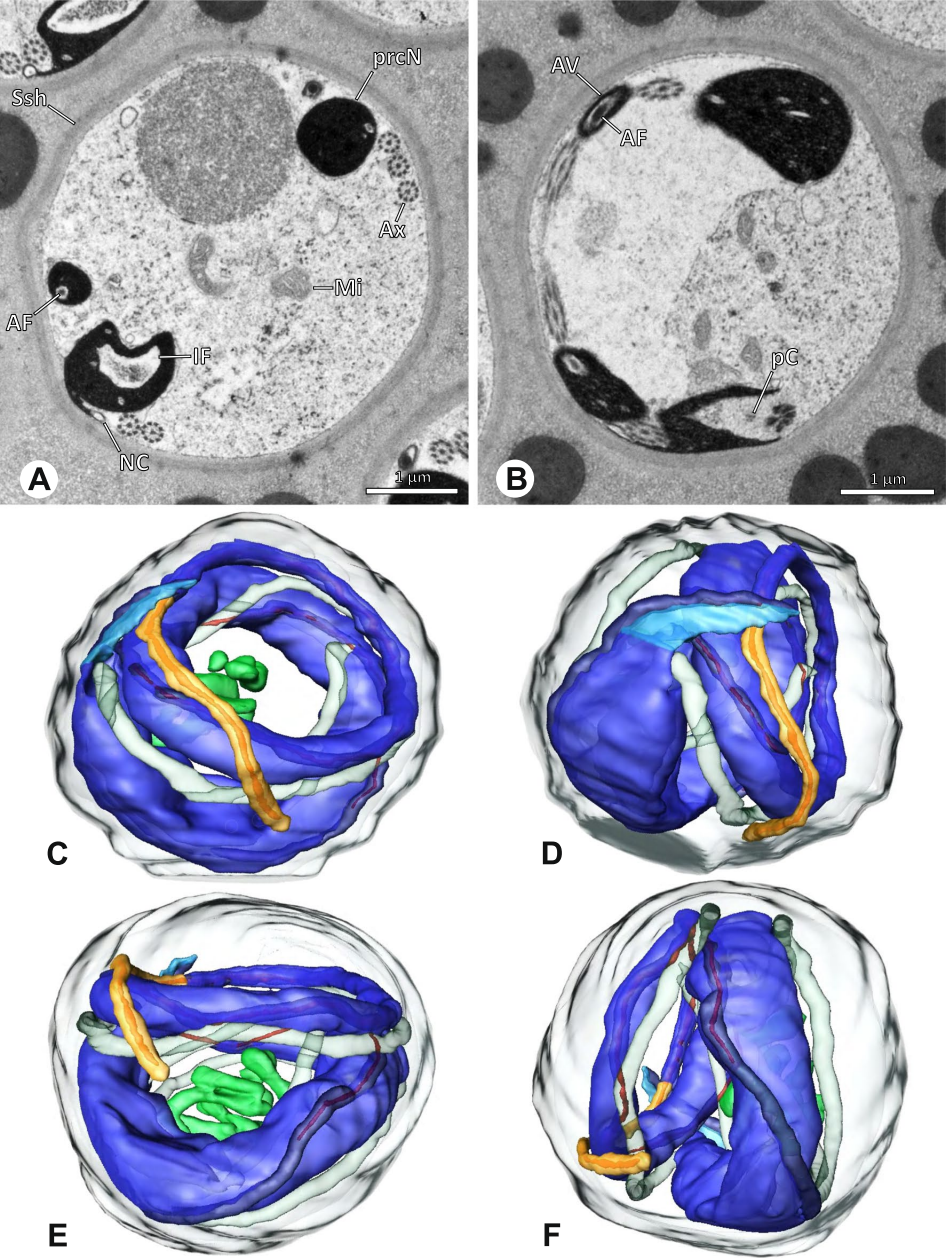
Ultrastructure and 3D surface reconstruction of cleistospermia of *Smeringopus cylindrogaster*. **A**, **B** Cleistospermia in the lumen of the deferent duct. Note the small amount of slightly electron dense material in the IF as well as the aggregation of electron dense material in the cytoplasm (**A**). **C**, **D**, **E**, **F** 3D surface reconstruction of a cleistosperm

**Spermatozoa.** Acrosomal complex. Slender and cylindrical (Fig. 47C), subacrosomal space extends throughout the entire AV. AF thin, projects into the NC and leads through nearly two-thirds of the length of the prcN (Fig. 47E). Nucleus. Asymmetric, prcN tubular, getting slightly wider in posterior direction (Fig. 2B, D); NC shifting to a lateral position in the posterior two thirds of the prcN (Fig. 47B, F). IF short, containing only little glycogen (Fig. 47A). peN flat, triangular to cone-shaped (Fig. 47D). Chromatin condensation dense and homogenous.

**Sperm transfer form.** Spherical cleistospermia with a secretion sheath (Fig. 47A, B). Nucleus spirally coiled (Fig. 47C), AV resting on top of the prcN (Fig. 47D). Ax relatively short and coiled once, interlaced with the coiled nucleus (Fig. 47D, E). Cytoplasm mostly homogenous, with some distinct aggregation of electron dense material; mitochondria present in the cytoplasm (Fig. 47A).

**Notes on spermiogenesis.** Chromatin condensation in early spermatids starts in a scattered manner (Fig. 8E). AV in mid spermatids roundish (Fig. 46C). In mid to late spermatids, chromatin condenses in an interwoven to streak-like manner, the IF contains microtubules until late stages of spermiogenesis (Fig. 46A, B).

**Seminal secretions.** One type of secretion, globular and homogenously electron dense (Fig. 4E).

### Smeringopinae | *Smeringopus* cf. *roeweri* (Fig. 48)

**Fig. 48:**
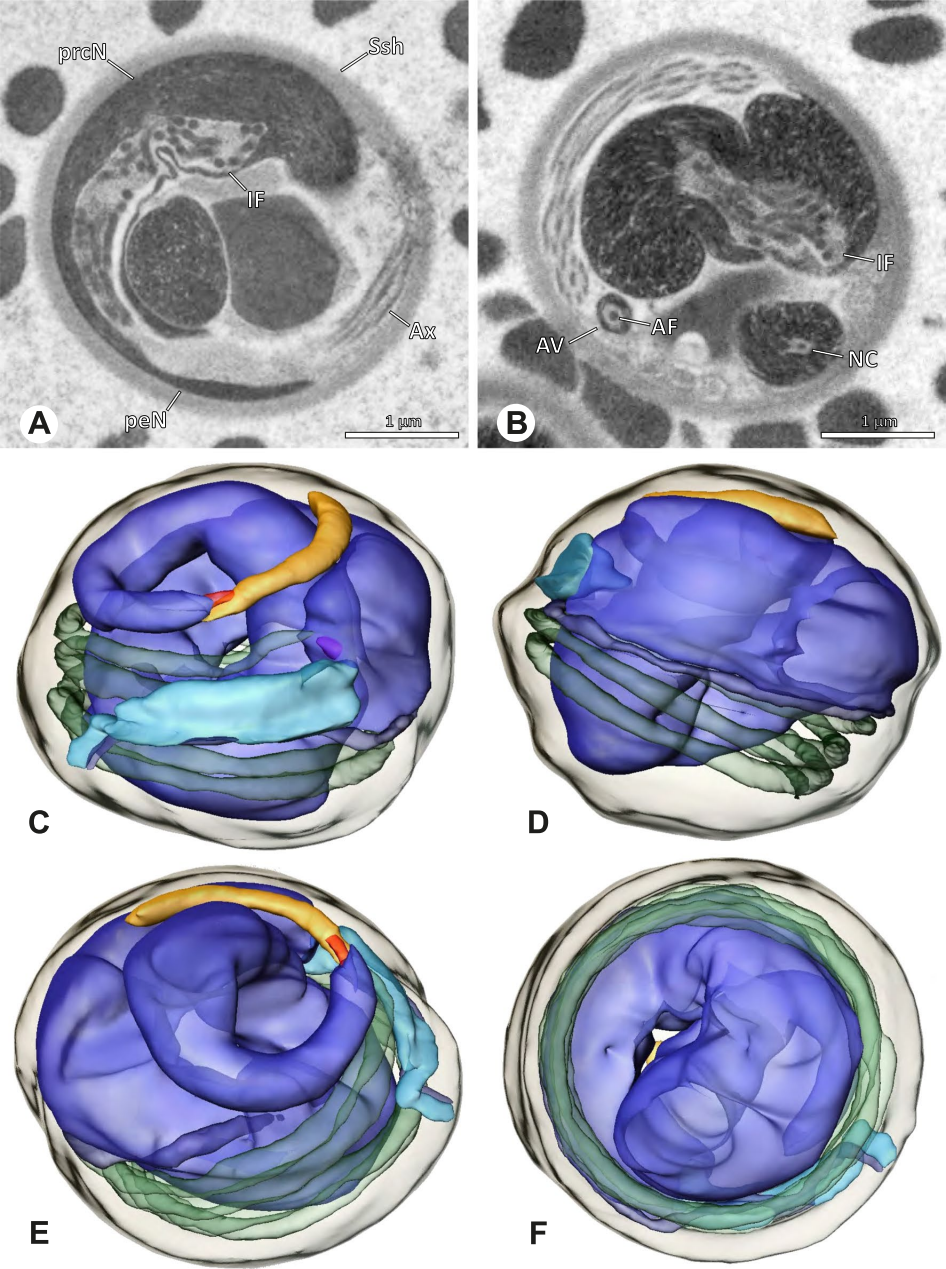
Ultrastructure and 3D surface reconstruction of cleistospermia of *Smeringopus* cf*. roeweri* (**A**, **B**) Cleistospermia in the lumen of the deferent duct. Note the electron dense filamentous material in the IF. **C**, **D**, **E**, **F** 3D surface reconstruction of a cleistosperm

**Spermatozoa. **Acrosomal complex. AV slender, cylindrical, subacrosomal space extends through the whole AV. AF stout and projecting into the NC as far as the first third of the prcN (Fig. 48E). Nucleus. asymmetric, prcN long and tubular, NC projects centrally through the anteriormost third of the prcN (Fig. 48B) before shifting to a peripheral position; IF deep, filled with thick filamentous electron dense material (Fig. 48A, B). peN flat (Fig. 48C). Chromatin condensation homogenous (Fig. 48A, B).

**Sperm transfer form.** Spherical cleistospermia surrounded by a secretion sheath (Fig. 48A, B); prcN coiled multiple times in a helical manner (Fig. 48C,E), acrosomal complex bent alongside the most anterior part of the prcN (Fig. 48C), Ax coiled four times around the nucleus (Fig. 48D).

**Notes on spermiogenesis. **In early stages, a fusion of several spermatids can be observed (Fig. 9E), which will later separate. In mid to late spermatids, microtubules are present in the IF (Fig. 5G in Michalik & Ramírez, 2014), which are reduced during the coiling process.

**Seminal secretions.** One type of secretion, small globular, homogenously electron dense (Fig. 4C).

### Smeringopinae | *Stygopholcus skotophilus* Kratochvíl, 1914 (Fig. 49)

**Fig. 49:**
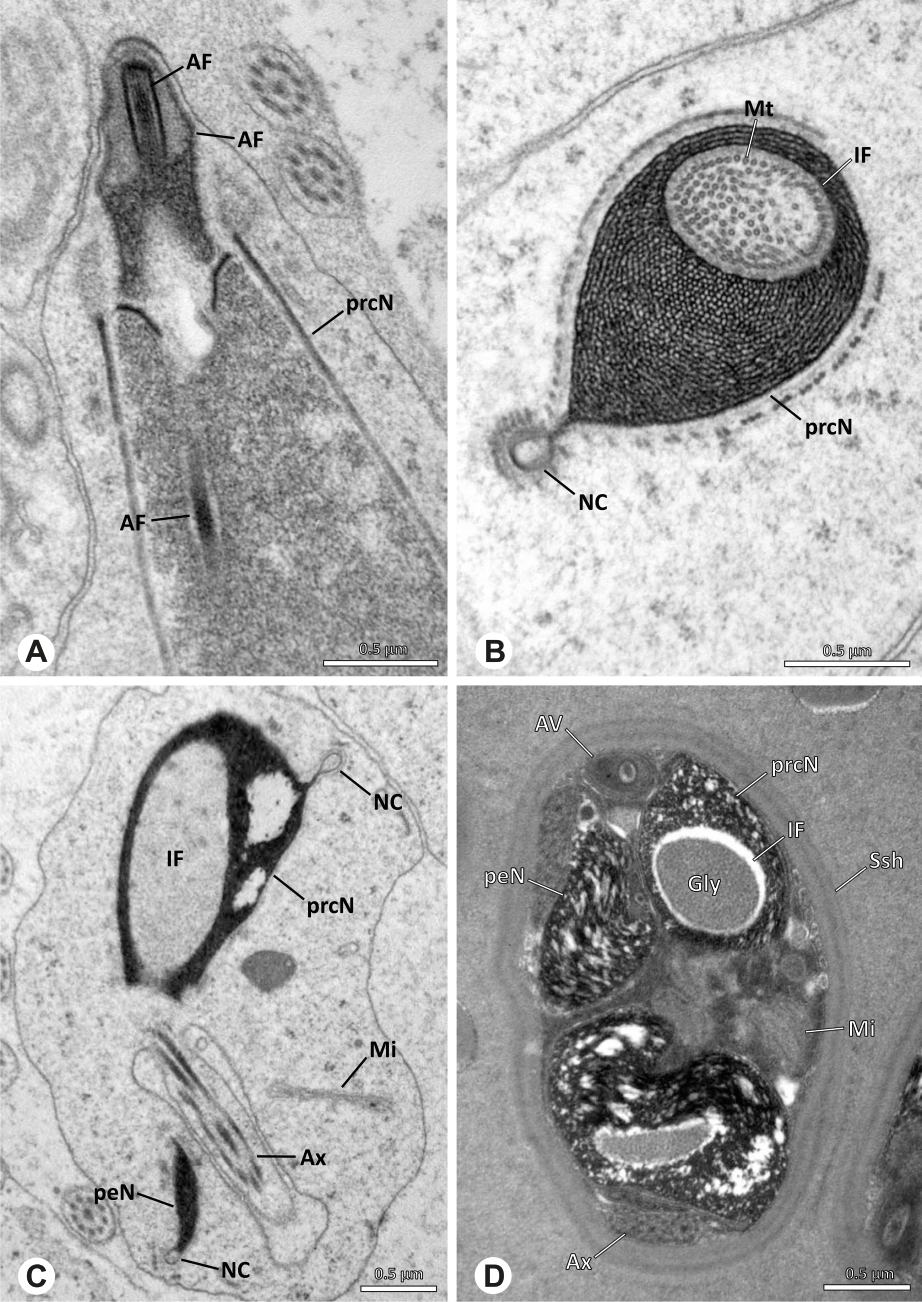
Spermiogenesis and cleistosperm of *Stygopholcus skotophilus*. TEM. **A** Early spermatid. **B** Mid spermatid, cross-section. At this stage, microtubuli are present in the IF. **C** Late spermatid. Note the heterogenous chromatin condensation pattern. **D** Cleistosperm in the lumen of the deferent duct. At the end of spermiogenesis, the IF becomes filled with glycogen

**Spermatozoa.** Acrosomal Complex**.** AV cylindrical, subacrosomal space extends through the entire AV, AF stout (Fig. 49A). Nucleus. Asymmetric, prcN cylindrical, IF extends into about half of the prcN and filled with glycogen (Fig. 49D). The NC runs within a projection along the prcN and is situated in the periphery within the peN (Fig. 49C). peN short. Chromatin condensation notably heterogenous (Fig. 49C, D).

**Sperm transfer form.** Spherical cleistospermia with a secretion sheath, Ax coiled four times around the nucleus. Cytoplasm heterogenous, mitochondria present (Fig. 49D).

**Notes on Spermiogenesis.** In early to mid spermatids, the IF is filled with microtubuli (Fig. 49B), which disintegrate in late stages and leave the IF filled with glycogen (Fig. 49C, D). The MM disintegrates at least partially before the coiling process (Fig. 49C).

**Seminal secretions.** Two types of secretions are present, both oval to elongated and homogenously electron dense but remarkably different in their size (Fig. 6A).

### Pholcinae | *Aetana* spp. (Figs. 50 and 51)

**Fig. 50:**
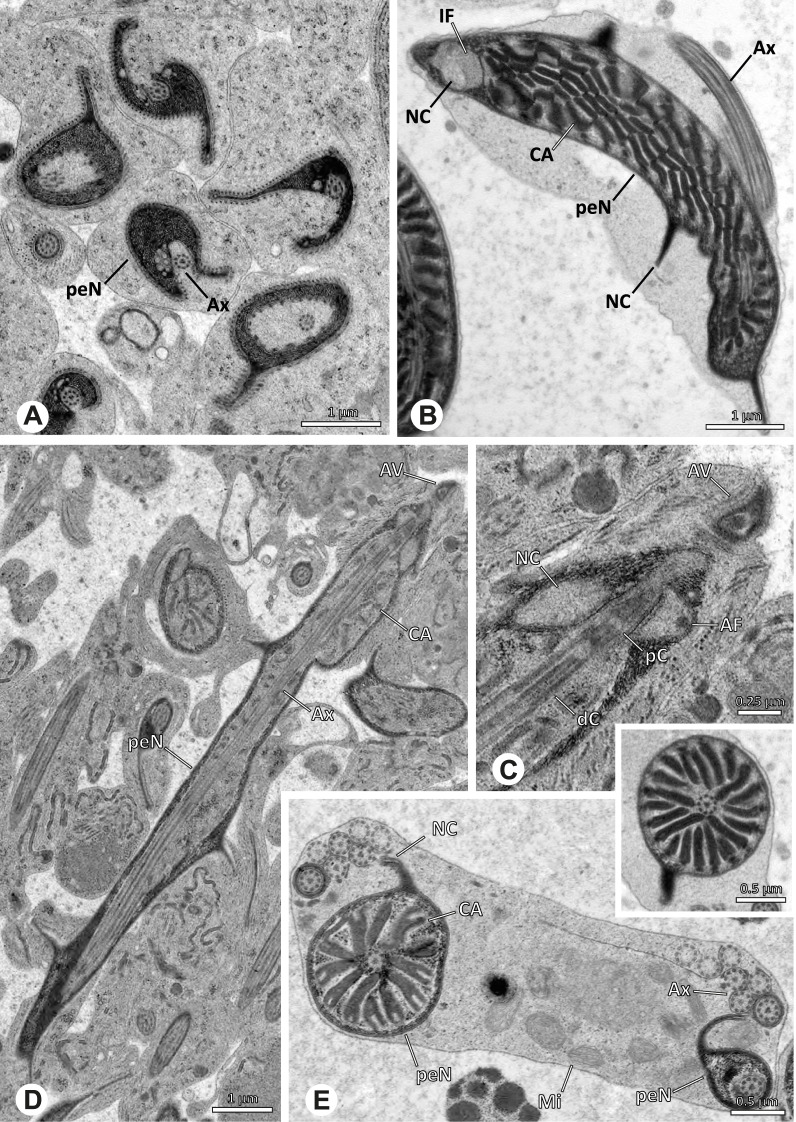
Spermiogenesis of *Aetana* spp. TEM. **A** *Aetana poring*. Late spermatids, the peN begins twisting around the axoneme. **B** *Aetana loboc*. Late spermatid, longitudinal section. Note the centriolar adjunct material surrounded by the peN. **C** *Aetana poring*. Mid to late spermatid, part of the acrosomal complex and the short prcN with both centrioles. **D** *Aetana poring*. Mid to late spermatid, longitudinal section. Note the peN surrounding the axoneme and the centriolar adjunct material. **E** Coiled spermatid of *Aetana poring*, cross section. Inset: cross section through the peN of *Aetana loboc* at the same stage of spermiogenesis

**Fig. 51:**
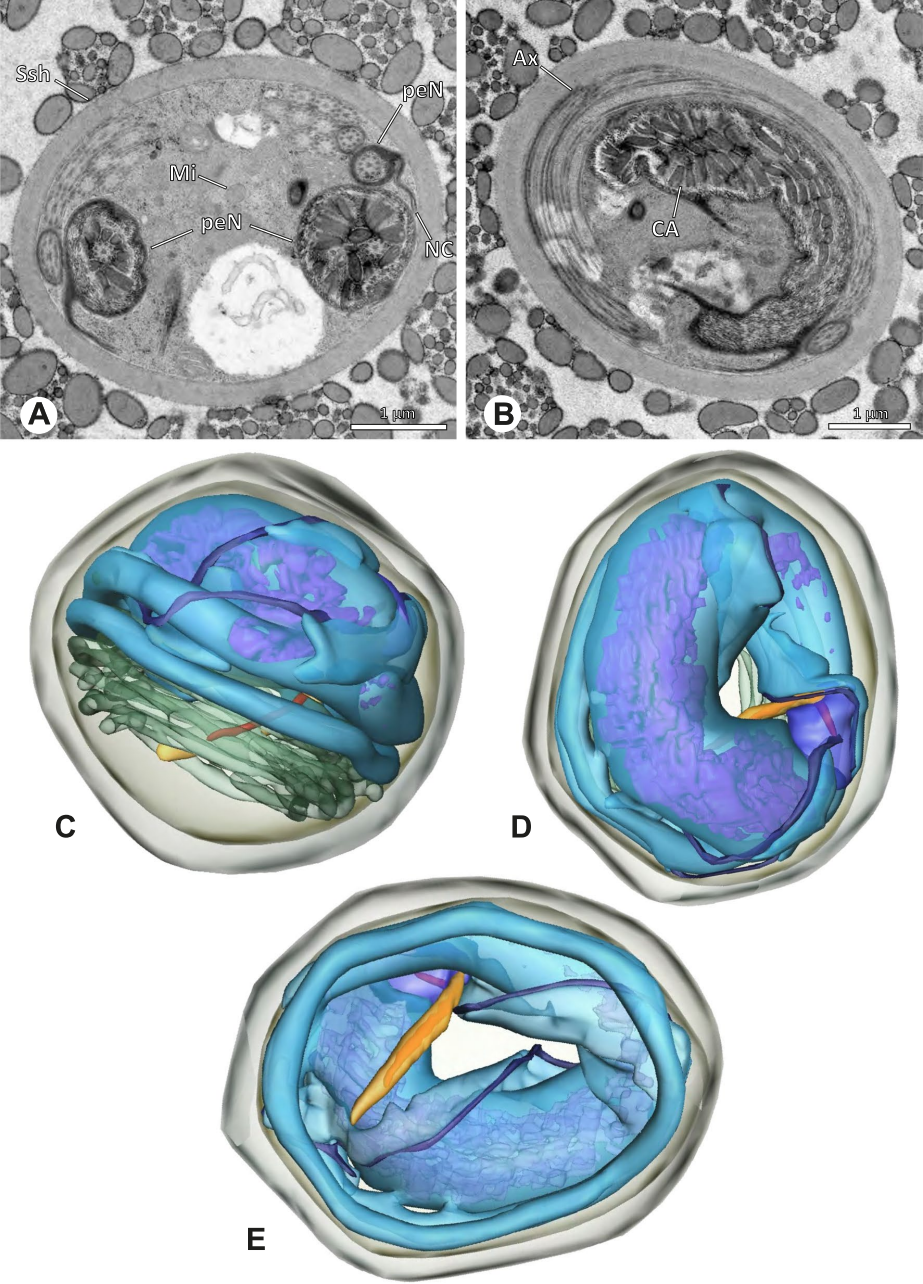
Ultrastructure and 3D surface reconstruction of cleistospermia of *Aetana poring*

**Spermatozoa.** Acrosomal complex. AV cylindrical, subacrosomal space extends throughout the entire AV, AF stout and short (Fig. 51E). Nucleus. Asymmetric, prcN very short (Fig. 50C and 51D). IF deep, projecting throughout the whole prcN, containing the centrioles (Fig. 50C). The wide NC runs laterally through the prcN and then shifts into a lateral extension alongside the peN (Fig. 50C); peN very long, wide in its anterior part and becoming very narrow towards posterior, closely surrounding the Ax (Figs. 50E and 51C). The peN is hollow and therefore tube-like, containing the Ax and centriolar adjunct material shaped as a collar of spoked lamellae surrounding the axoneme (Fig. 51A). The lamellae of the centriolar adjunct material differ slightly between the two studied species, as they can be either irregularly spoked in *A. poring* (Fig. 50D) or helically spoked and electron dense in *A. loboc* (Fig. 50B). Chromatin condensation dense and homogenous.

**Sperm transfer form.** Spherical to oval cleistospermia surrounded by a secretion sheath (Fig. 51A, B). AV and prcN bent compactly in the center, with the peN coiled around them, filling more than half of the cleistosperm and transforming into the coiling of the axoneme in its posterior portion (Fig. 50C, E). Ax coiled four times beside the nucleus, (Fig. 51C). Cytoplasm mostly homogenous, mitochondria present (Fig. 51A, B).

**Notes on spermiogenesis. **Early to mid spermatids show a fibrillar chromatin condensation. Most conspicuous is the organization of the peN, which is very elongated and contains the axoneme. At later stages, the peN elongates, widens, and starts winding around the axoneme (Fig. 50B). During spermiogenesis, the peN fuses, forming a continuous and hollow tube containing the axoneme (Fig. 50D). The inner surface of the peN shows particular pin-like projections in a regular pattern (Fig. 50B), which might be the “organizational centers” of the later forming centriolar adjunct material (Fig. 50E). The NC is situated in thin lateral extensions of the peN (Fig. 50B). In late spermatids, the centriolar adjunct material is fully developed as spoked lamellae around the axoneme (Fig. 50D, E). An indentation on each lamella corresponds with the “organizational centers”, which are reduced at the end of spermiogenesis in *A. poring* (Fig. 50E), while such indentations are absent in *A. loboc* (Fig. 50E inset). During the coiling process, the NC is bent slightly towards the nucleus and appears curved (Fig. 50E).

**Seminal secretions.** One type of secretion, consisting of spherical, homogenous structures of different sizes aggregated to spherical patches, with smaller particles in the center surrounded by larger ones (Fig. 4L).

### Pholcinae | *Belisana* cf. *kinabalu* (Figs. 52 and 53)

**Fig. 52:**
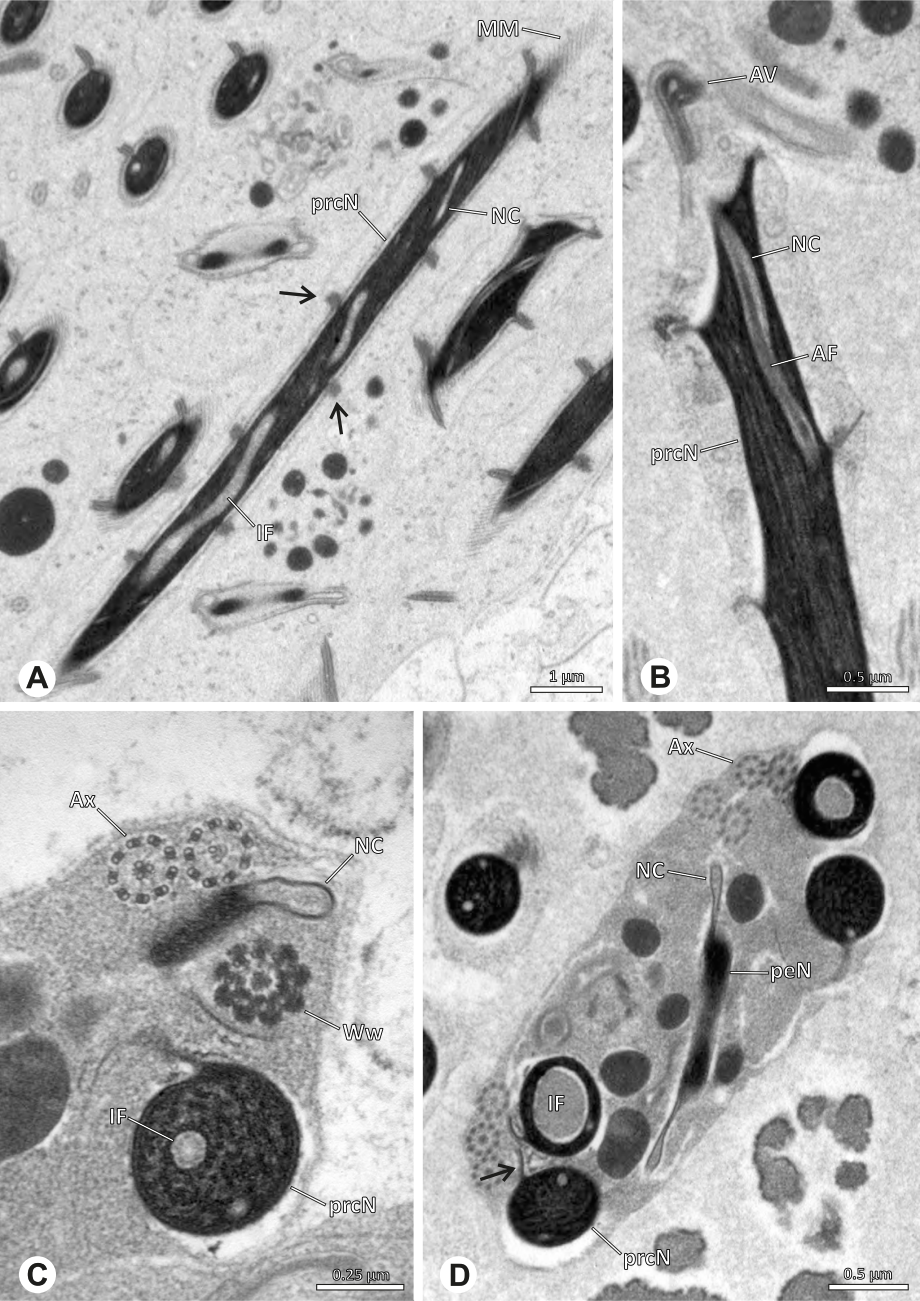
Spermiogenesis in *Belisana* cf. *kinabalu*. TEM. **A**, **B** Late spermatids, longitudinal sections. The prominent helical band (**A**, arrows) is well visible. **C** Coiled spermatid, note the electron dense material in “water wheel” configuration. **D** Coiled spermatid, cross section. Note the helical band (arrow)

**Fig. 53:**
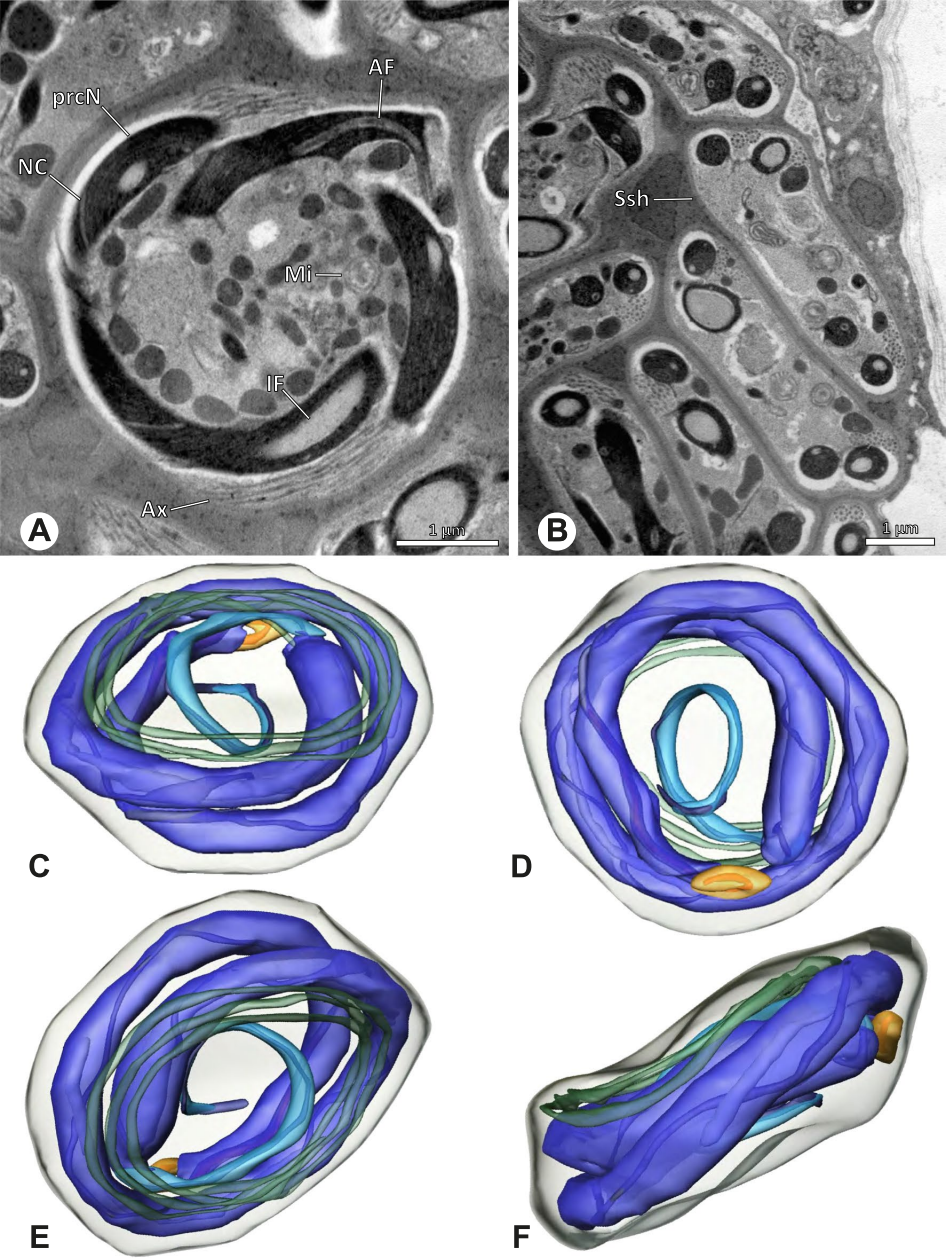
Ultrastructure and 3D surface reconstruction of cleistospermia of *Belisana* cf. *kinabalu*. Note the flat and disc-like appearance of the cleistospermia in (**B**)

**Spermatozoa.** Acrosomal complex. AV cylindrical, subacrosomal space extends throughout the entire AV, giving rise to the thin AF (Fig. 53D). Nucleus. prcN long and slender, nearly radially symmetric (Fig. 53C), with a helically contorted surface (Fig. 53A). IF occupies about half of the prcN (Fig. 53E, F). Centrioles in contact with electron dese material in “water wheel” configuration [sensu 16] (Fig. 52C). NC runs helically through the periphery of the nucleus (Figs. 52D and 53A,D). peN slender (Fig. 53D).

**Sperm transfer form.** Flat, disc-like cleistospermia, surrounded by a secretion sheath (Fig. 53B). prcN spirally coiled (Fig. 53C, E). The peN is slightly winding centrally within the coiling prcN (Fig. 53D, E). Axoneme coiled three times beside the nucleus (Fig. 53F). Cytoplasm heterogenous, with numerous electron-dense vesicles; mitochondria present (Fig. 53A).

**Notes on spermiogenesis.** Beginning in late stages, spermatids show a helical band of thin extensions of nuclear material (Fig. 52A, B), which remains present also after the coiling process (Fig. 52C, D).

### Pholcinae | *Cantikus sabah* (Huber, 2011) (Fig. 54)

**Fig. 54:**
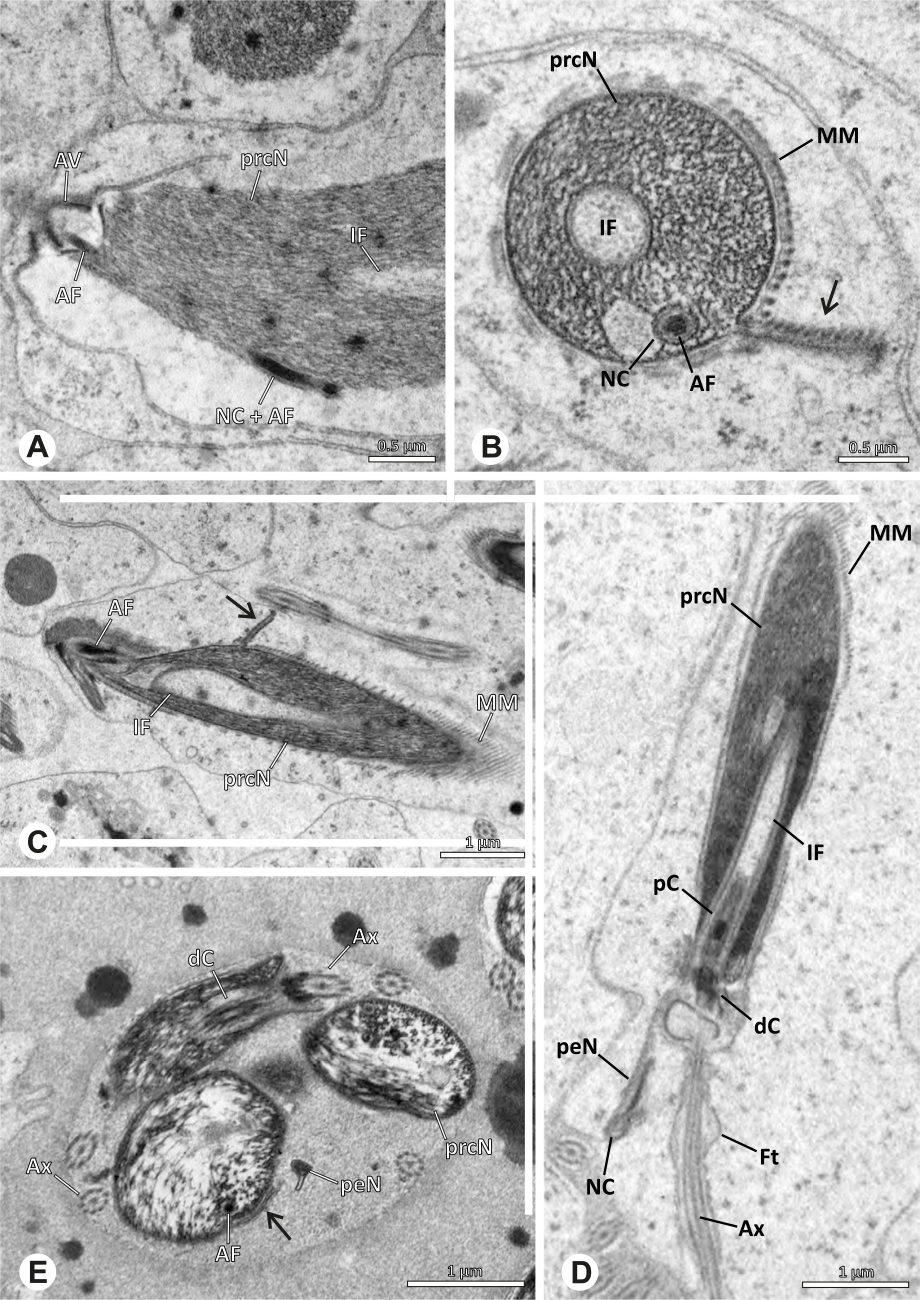
Spermiogenesis and cleistosperm in *Cantikus sabah*. TEM. **A** Early spermatid. The nuclear canal is running in the periphery of the nucleus. **B** Mid spermatid, cross-section. Note the developing helical band of nuclear material (arrow). **C** Mid to late spermatid, longitudinal section. Note the deep implantation fossa. **D** Mid to late spermatid, longitudinal section. The proximal centriole is notably elongated. **E** Cleistosperm in the lumen of the deferent duct. The helical band of nuclear material is also present after coiling (arrow)

**Spermatozoa.** Acrosomal complex. AV cylindrical, subacrosomal space extends throughout the entire AF. AF stout (Fig. 54B). Nucleus. prcN tubular, nearly radially symmetric (Fig. 54B), helical band of nuclear material present (Fig. 54B arrow, C arrow). IF deep (Fig. 54A, C). Proximal centriole (pC) elongated (Fig. 54D). NC runs in the periphery of the nucleus (Fig. 54A, D). peN short (Fig. 54D). Chromatin condensation homogenous.

**Sperm transfer form.** Spherical to oval cleistospermia, surrounded by a secretion sheath; Ax coiled three times around the nucleus, cytoplasm homogenous (Fig. 54E).

**Notes on spermiogenesis.** In mid spermatids, the helical band of nuclear material develops (Fig. 54B, C) and remains present in mature sperm (Fig. 54E arrow).

**Seminal secretions.** One type of secretion, roundish and electron dense (Fig. 6D).

### Pholcinae | *Leptopholcus guineensis* Millot, 1941 (Figs. 55 and 56)

**Fig. 55:**
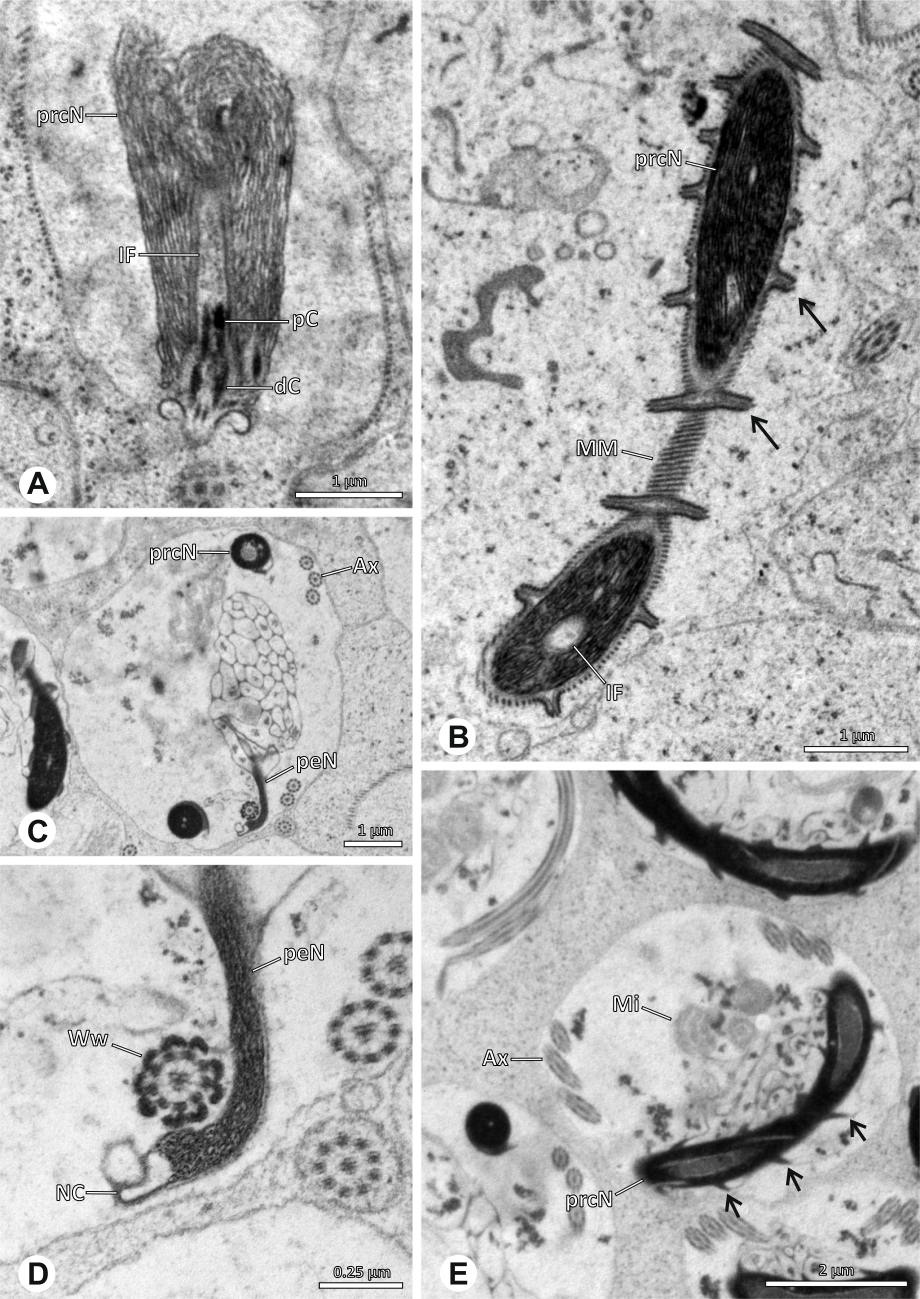
Spermiogenesis of *Leptopholcus guineensis*. TEM. **A** Early spermatid. The adjacent position of the two centrioles is well visible. **B** Mid to late spermatid. Note the helical band of nuclear material on the nuclear surface (arrows). **C** Coiled spermatid, cross-section. Note the thin peN. **D** Detail of the anterior part of the axoneme. The electron dense material in “water wheel” configuration, surrounding the centrioles and the most anterior part of the axoneme, is well visible. **E** Early Cleistosperm in the distal testis lumen. Note the presence of the helical band (arrows)

**Fig. 56:**
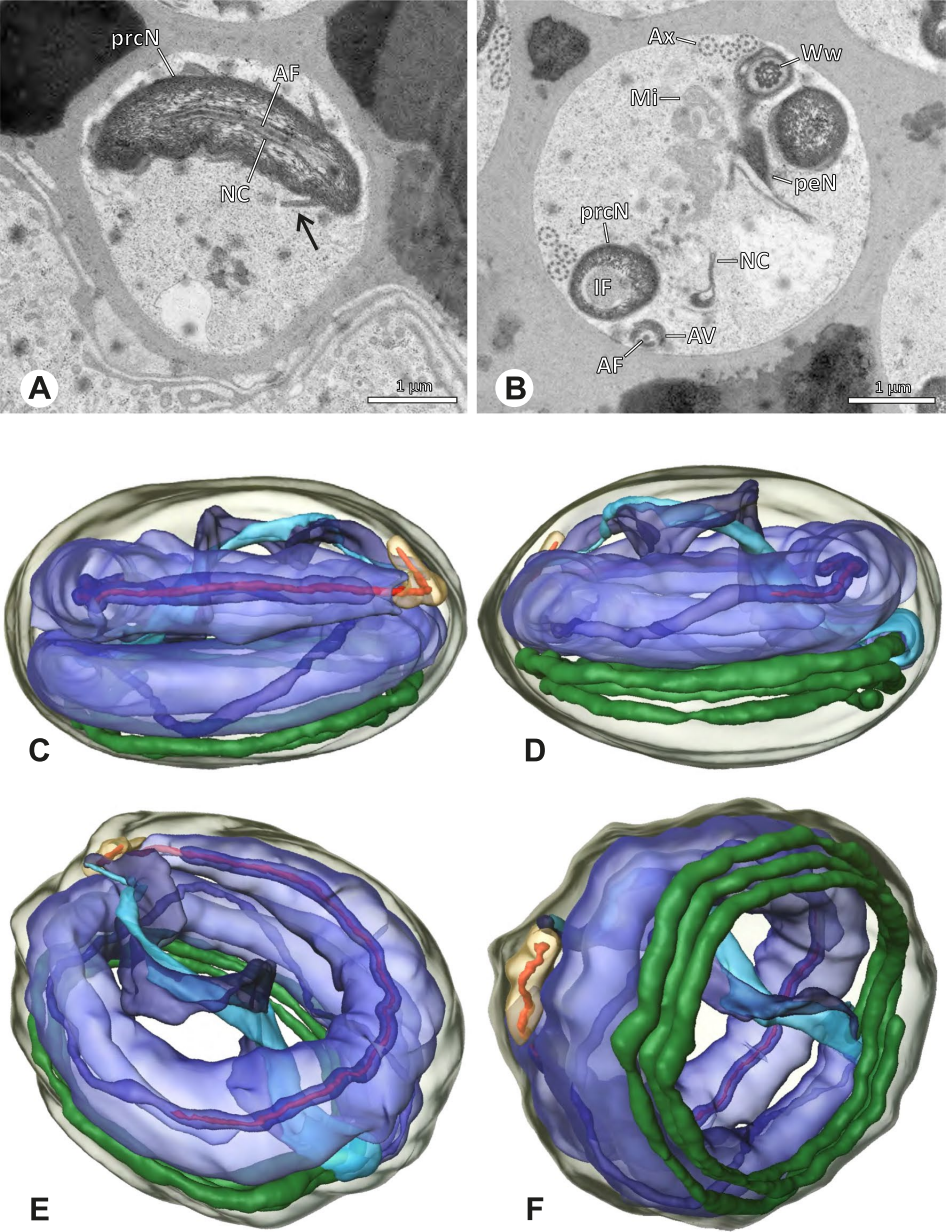
Ultrastructure and 3D surface reconstruction of the cleistospermia of *Leptopholcus guineensis*. **A** Cleistosperm in the lumen of the deferent duct. Note the helical band (arrow) and the centrally situated nuclear canal. **B** Cleistosperm in the lumen of the deferent duct. Note the electron dense “water wheel” and the thin peN. **C**, **D**, **E**, **F** 3D surface reconstruction of a cleistosperm

**Spermatozoa.** Acrosomal complex. AV cylindrical, subacrosomal space extends throughout the entire AV, giving rise to the thin AF (Fig. 56C, F). AF projecting through approximately one third of the prcN (Fig. 56E). Nucleus. prcN cylindrical, nearly radially symmetric (Fig. 56), with a helical band of nuclear material on its surface (Fig. 56A). NC runs centrally through the anterior part of prcN (Fig. 56A, E). IF deep, extends into more than half of the prcN (Fig. 56E). Centrioles in contact with electron dense material in “water wheel” configuration (Figs. 55D and 56B). peN flat and about half the length of the prcN (Fig. 56D, E). Chromatin condensation homogenous.

**Sperm transfer form.** Spherical cleistospermia surrounded by a secretion sheath (Fig. 56A, B). prcN spirally coiled, with the peN bent centrally inside the spiral (Fig. 56F). Ax coiled three times beside the nucleus (Fig. 56D, F). Cytoplasm homogenous, mitochondria present (Fig. 56A, B).

**Notes on spermiogenesis.** In late spermatids, the helical band is developing (Fig. 55B). The electron dense material in “water wheel” configuration also forms at this stage (Fig. 55D).

**Seminal secretions.** One type of secretion, irregular roundish, homogenously electron dense (Fig. 4N).

### Pholcinae | *Metagonia* cf. *petropolis* (Figs. 57 and 58)

**Fig. 57:**
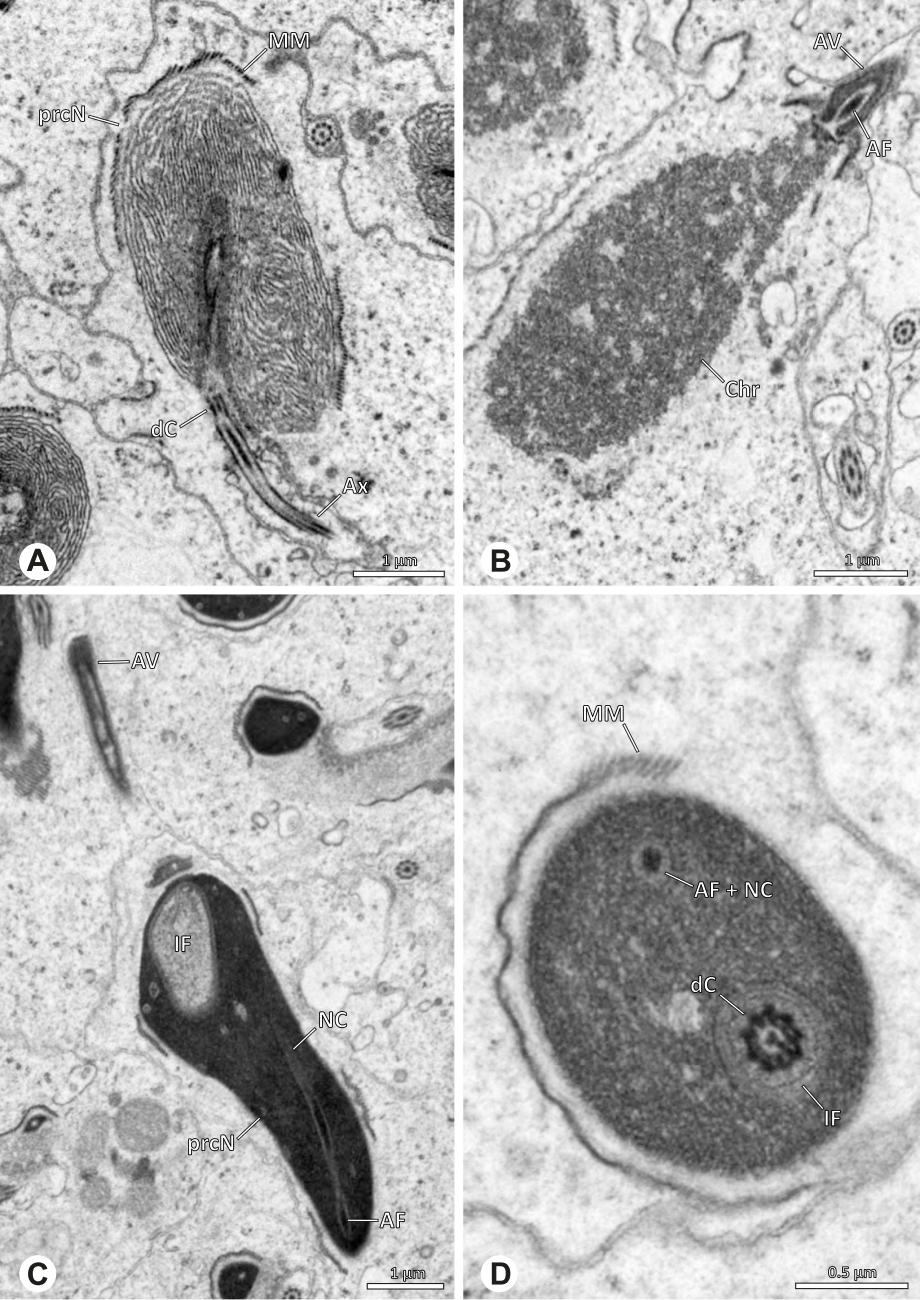
Spermiogenesis of *Metagonia* cf. *petropolis*. TEM. **A**, **B** Early spermatid. Note the condensation pattern. **C** Mid to late spermatid. Note the elongated acrosomal vacuole, the peripheral nuclear canal and the gaps in the manchette of microtubules. **D** Late spermatid. The electron dense material around the centrioles begins to form

**Fig. 58:**
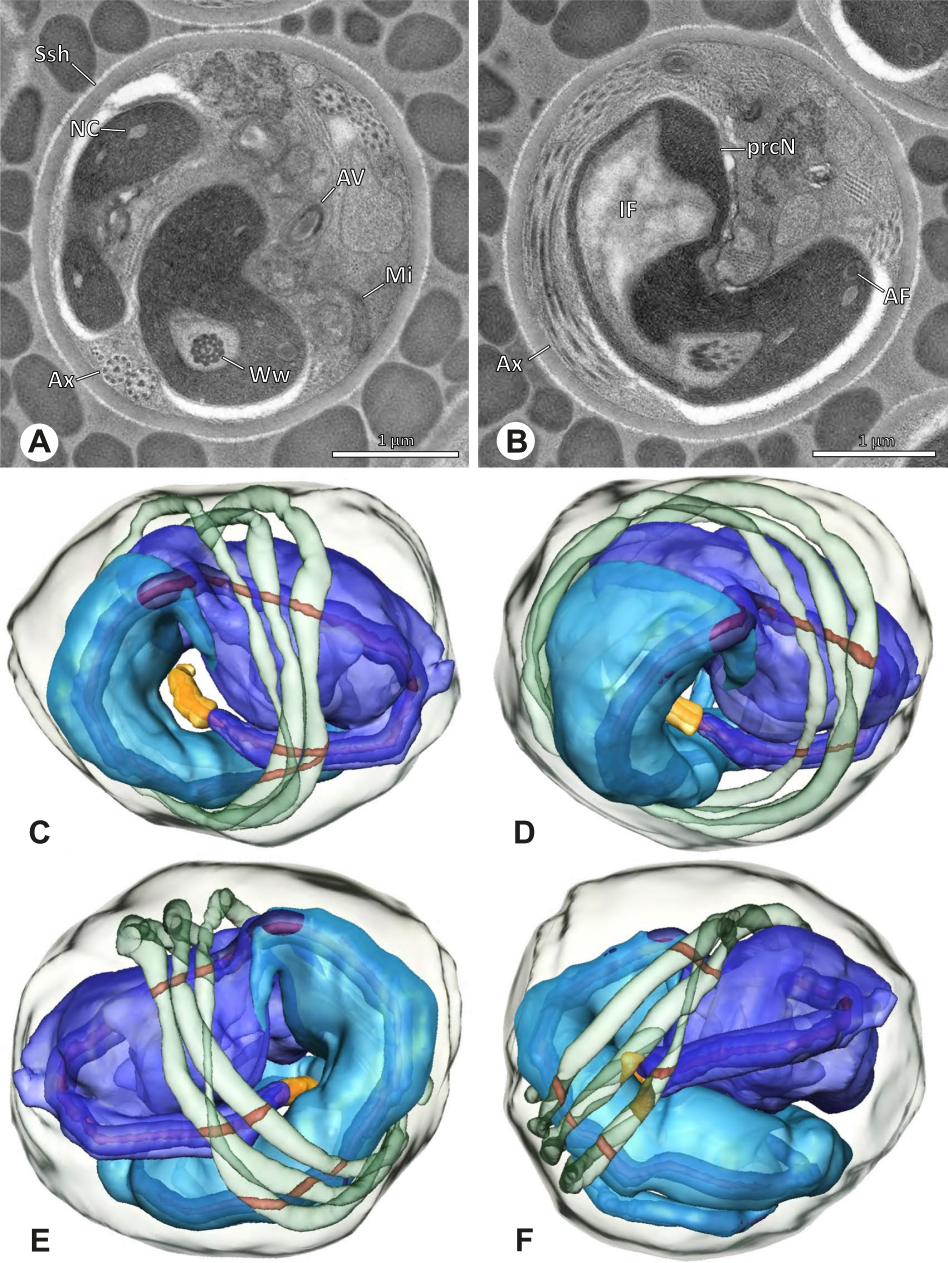
Ultrastructure and 3D surface reconstruction of the cleistospermia of *Metagonia* cf. *petropolis*. **A**, **B** Cleistospermia in the lumen of the deferent duct, cross-section. Note the electron dense material in “water wheel” configuration and the central nuclear canal within the prcN. **C**, **D**, **E**, **F** 3D surface reconstruction of a cleistosperm

**Spermatozoa.** Acrosomal complex. AV cylindrical, subacrosomal space extends throughout the entire AV. AF stout, extends into the nuclear canal where it ends approximately halfway inside the peN (Fig. 58C, E). Nucleus. Asymmetric, prcN cone-shaped, becoming tubular in its anterior part (Fig. 58C, E). NC runs centrally through the prcN and is shifted to the periphery within the peN (Fig. 58B, E). IF extends up to half of the prcN (Fig. 58B, E). Centrioles in contact with electron dense material in “water wheel” configuration (sensu [16]). (Figs. 57D and 58A). Chromatin condensation homogenous.

**Sperm transfer form.** Spherical cleistospermia, surrounded by a secretion sheath (Fig. 58A, B). prcN and peN bent once along the longitudinal axis centrally in the transfer form (Fig. 58E), axoneme coiled three times, partly around the nucleus (Fig. 58C, F). Cytoplasm heterogenous, mitochondria present (Fig. 58A, B). Cluster of microtubules present in the cytoplasm (Fig. 58A, B).

**Notes on Sspermiogenesis.** Mid spermatids show a filamentous and spirally wound, streak-like condensation pattern (Fig. 57A). In late spermatids, chromatin condensation is homogenous and dense, the AV is elongated and the electron dense material surrounding the centrioles begins to form (Fig. 57C, D). The manchette of microtubules only partially surrounds the nucleus during spermiogenesis.

**Seminal secretions.** One type of secretion, roundish, homogenously electron dense (Fig. 4O).

### Pholcinae | *Micropholcus fauroti *(Simon, 1887) (Fig. 59)

**Fig. 59:**
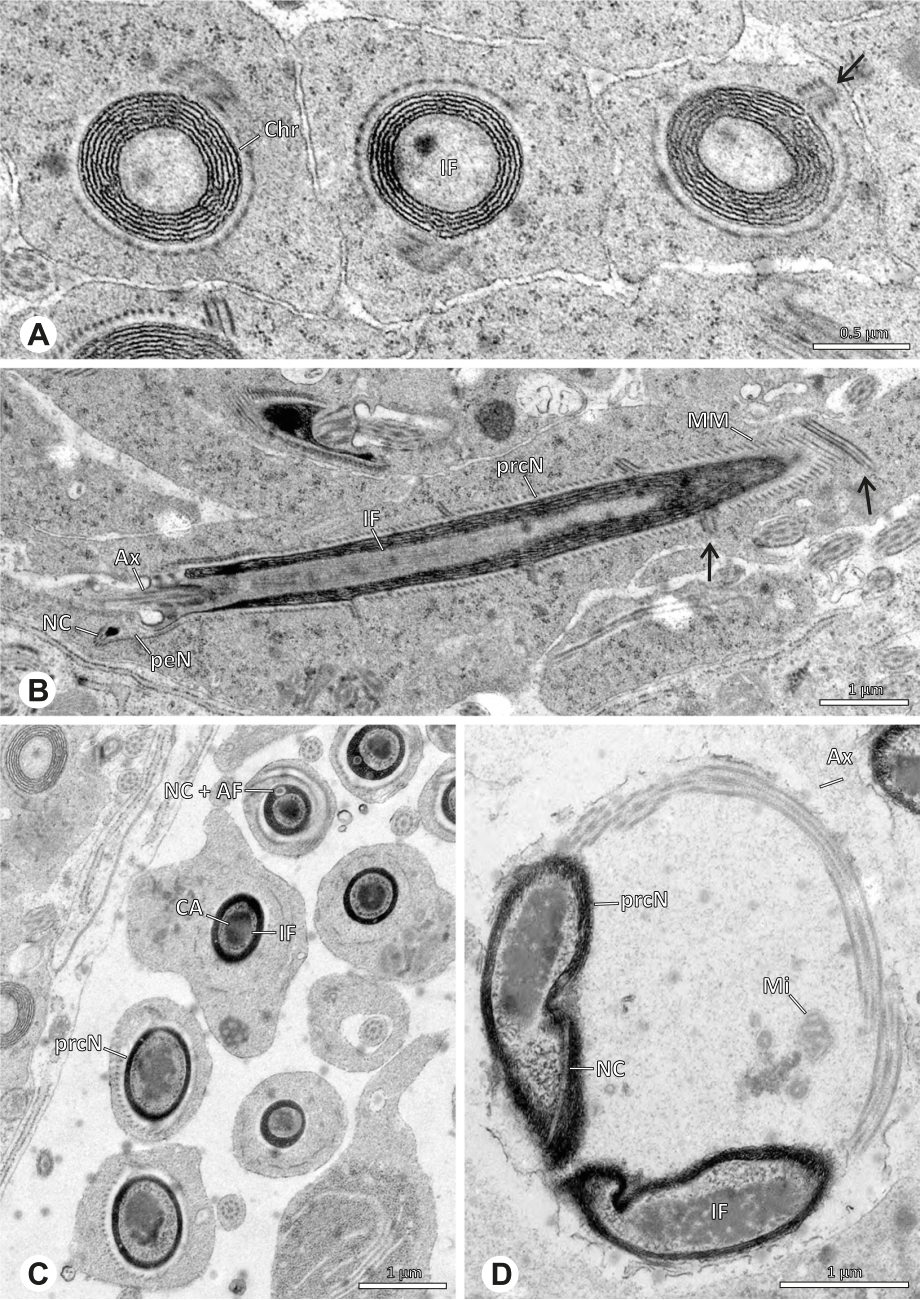
Spermiogenesis of *Micropholcus fauroti*. TEM. **A** Mid spermatids, cross section. Note the chromatin condensation pattern and the helical band (arrow). **B** Mid to late spermatid. Note the helical band of nuclear material on the surface of the prcN (arrows) and the short peN. **C** Late spermatids, cross section. The nuclear canal shifts into the periphery of the prcN as soon as it meets the implantation fossa. **D** Coiled spermatid. Note the electron dense content of the implantation fossa

**Spermatozoa. **Acrosomal complex. AF ends clearly before the axonemal basis. Nucleus. prcN tubular, nearly radially symmetric, helical band of nuclear material present (Fig. 59B). IF deep, filled with electron dense granules and containing electron dense homogenous anterior centriolar adjunct material (Fig. 59C, D). NC runs centrally through the prcN and shifts to the periphery within the peN (Fig. 59B). Chromatin condensation homogenous.

**Sperm transfer form. **Spherical cleistospermia, surrounded by a secretion sheath.

**Notes on Spermiogenesis.** Mid-spermatids show a regular chromatin condensation organized in a spiral manner (Fig. 59A). During this stage, short helical extensions are present (Fig. 59A arrow), which in late spermatids form a helical band surrounding the prcN (Fig. 59B arrow). Late spermatids show a dense and still streak-like condensation pattern, with a very elongated nucleus; the IF is deep, voluminous, and filled with electron dense material (Fig. 59C).

### Pholcinae | *Panjange camiguin* Huber, 2015 (Figs. 60, 61)

**Fig. 60:**
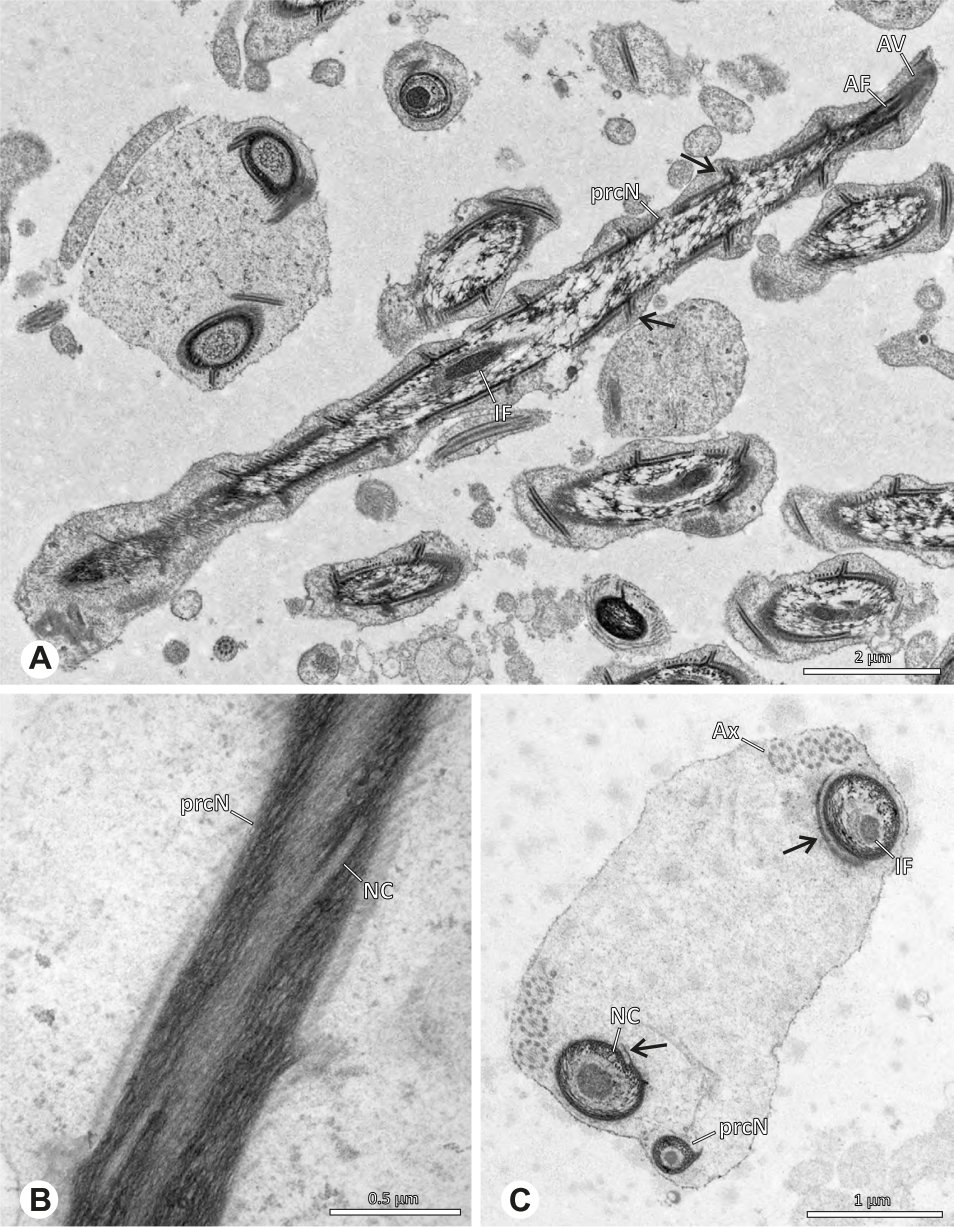
Spermiogenesis of *Panjange camiguin* TEM. **A** Late spermatid, longitudinal section. The cylindrical acrosomal vacuole as well as the deep IF filled with granular material are well visible. Note the helical band of nuclear material along the precentriolar portion of the nucleus (arrows). **B** Late spermatid. As soon as the nuclear canal meets the IF, it is shifted to the periphery of the prcN. **C** Early sperm transfer form. The helical band is also present in the coiled state (arrow)

**Fig. 61:**
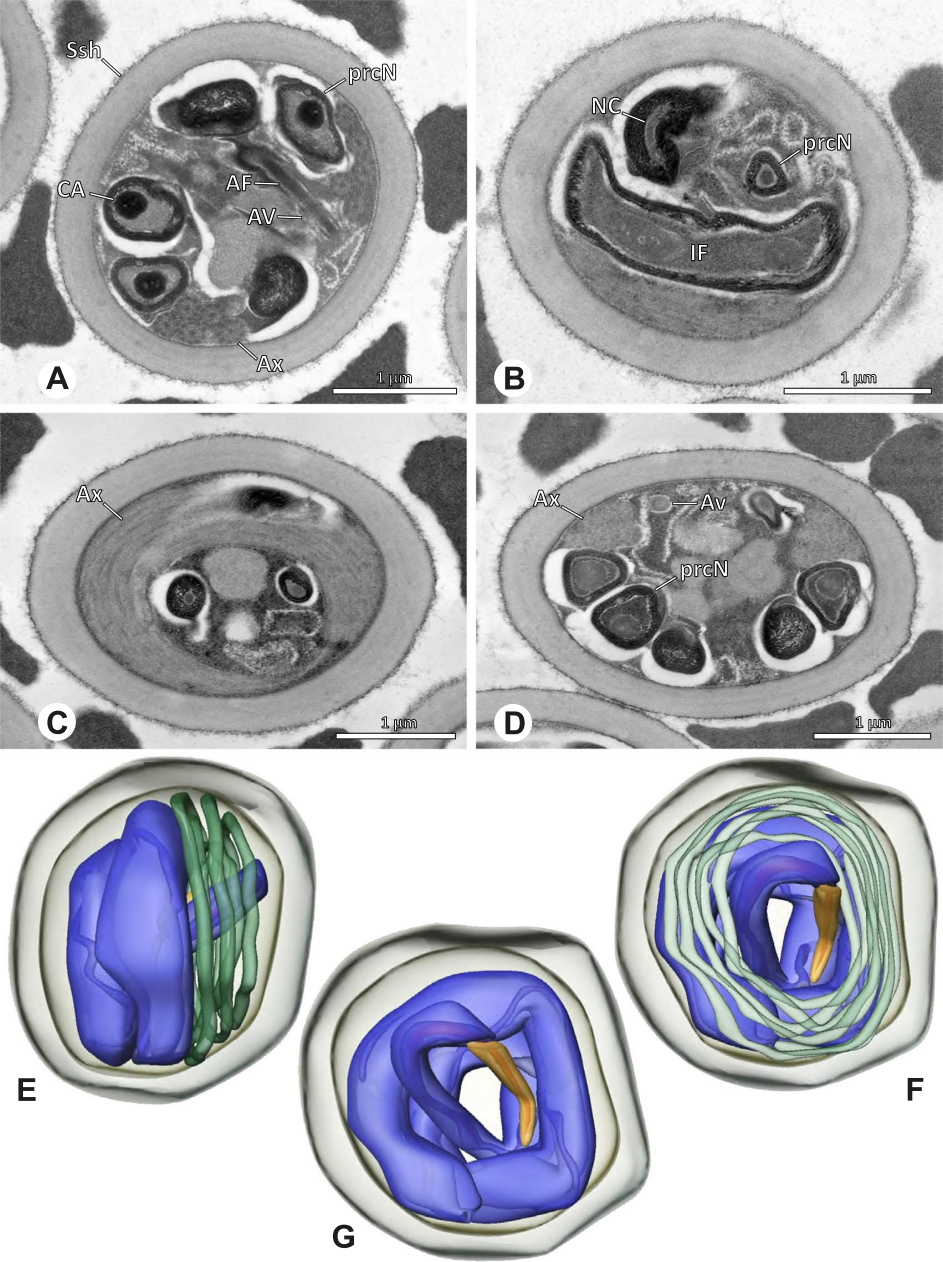
Ultrastructure and 3D surface reconstruction of the cleistospermia of *Panjange camiguin*. **A**, **B**, **C**, **D** Cleistospermia in the lumen of the deferent duct. Note the multilayered secretion sheaths and the electron dense anterior centriolar adjunct material (**A**) within the IF filled with granular material (**B**). The coiling of the axoneme besides the coiled nucleus is visible in (**C**). **E**, **F**, **G** 3D surface reconstruction of a cleistosperm

**Spermatozoa.** Acrosomal complex. AV conical, subacrosomal space extends throughout the entire AV. AF stout, projecting centrally through the NC into the anterior part of the prcN (Figs. 60A and 61G). Nucleus. prcN tubular, nearly radially symmetric (Fig. 61G). Helical band of nuclear material around the prcN present (Fig. 60A arrows). NC runs helically in the periphery of the prcN (Figs. 60B and 61E). IF deep, extends centrally into more than half of the prcN and heterogeneously filled with granular material as well as heterogeneous electron-dense anterior centriolar adjunct material (Fig. 60A). peN very short. Chromatin condensation dense and homogenous.

**Sperm transfer form.** Spherical to oval cleistospermia, surrounded by a secretion sheath (Fig. 61A-D). AV and anterior tip of the prcN together form a loop situated centrally in the cleistosperm (Fig. 61E, G). Ax coiled five times beside the nucleus and surrounding the anterior portion of the prcN (Fig. 61E).

**Notes on spermiogenesis.** Late spermatid stages show a very elongated prcN, equipped with a helical band (Fig. 60A arrows) At this stage, the IF is deep, narrow, and filled with electron dense material (Fig. 60A, B). During the coiling process, the IF widens notably and occupies most of the prcN (Fig. 60C).

**Seminal secretions.** One type of secretion, roundish to elongated, homogenously electron dense (Fig. 4M).

### Pholcinae | *Pehrforsskalia conopyga* Deeleman-Reinhold & van Harten, 2001 (Figs. 62 and 63)

**Fig. 62:**
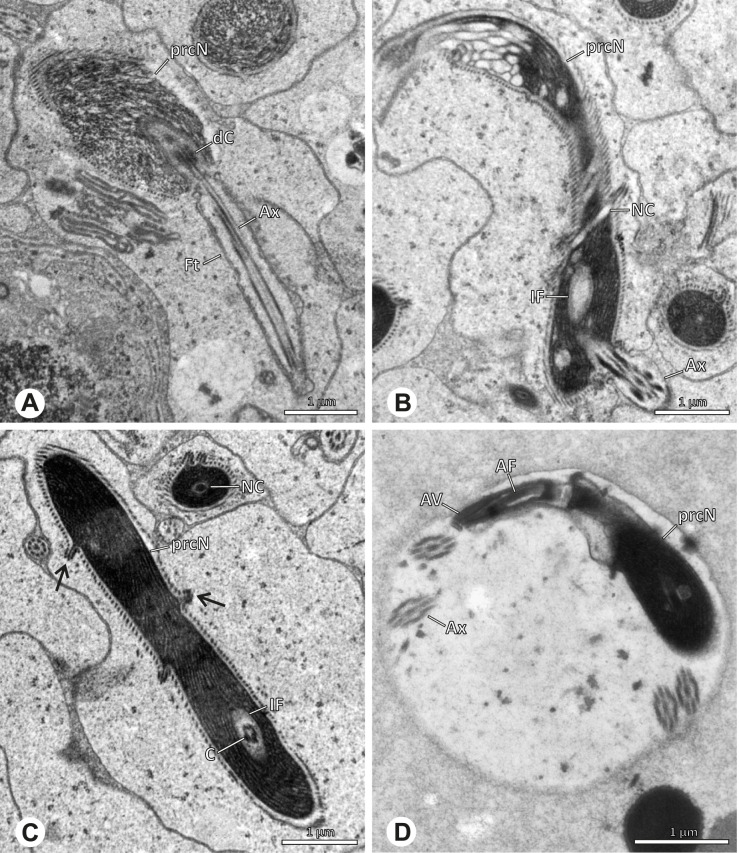
Spermiogenesis of *Pehrforsskalia conopyga*. TEM. **A** Early spermatid. Note the granular condensation pattern of the chromatin. **B** Mid spermatid. The chromatin condensation is mostly dense, with few more lightly condensed areas. **C** Late spermatid. Note the helical band of nuclear material along the prcN (arrow). **D** Coiled spermatid within the lumen of the testes. Note the cylindrical AV

**Fig. 63:**
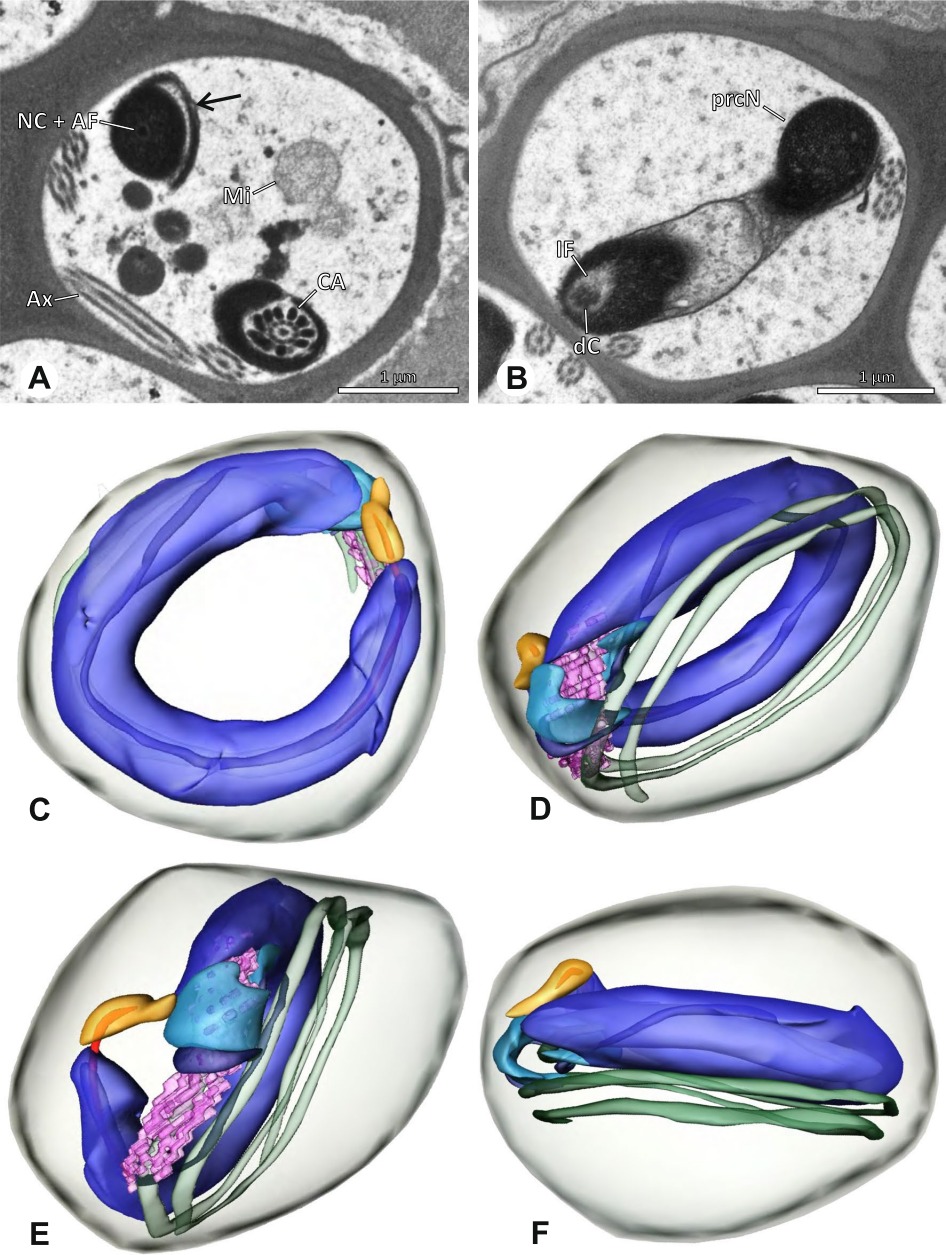
Ultrastructure and 3D surface reconstruction of the sperm transfer form of *Pehrforsskalia conopyga*. **A**, **B** Cleistospermia in the lumen of the deferent duct. Note the lamellar centriolar adjunct material and the helical band (arrow) in (**A**). **C**, **D**, **E**, **F** 3D surface reconstruction of a cleistosperm

**Spermatozoa.** Acrosomal complex. AV cylindrical, subacrosomal space extends throughout the entire AV (Figs. 62D and 63F). AF extends in the NC through the anterior third of the prcN. Nucleus. prcN long, tubular, nearly radially symmetric, with a reduced helical band (Fig. 63A arrow). NC runs centrally through the prcN and is situated in the periphery within the peN (Fig. 63C). IF extends into less than half of the prcN. Posterior centriolar adjunct material present as a collar of short layered lamellae (Fig. 63A). peN short and flat (Fig. 63E). Chromatin condensation homogenous.

**Sperm transfer form.** Spherical cleistospermia surrounded by a thin secretion sheath (Fig. 63A, B). Nucleus coiled once (Fig. 63F), Ax coiled twice beside the nucleus (Fig. 63D, F). Cytoplasm homogenous, mitochondria present (Fig. 63A).

**Notes on Spermiogenesis.** In early spermatid stages, chromatin condensation appears to be granulomatous. In mid to late spermatids, the chromatin condensation appears mostly very dense, while some areas are much more lightly condensed (Fig. 62B). The prcN has a helical band (Fig. 62C, D arrows), and the centriolar adjunct material starts to form at this stage.

**Seminal secretions.** One type of secretion, round and homogenously electron dense (Fig. 5A).

### Pholcinae | *Pholcus* spp. (Figs. 64, 65 and 66)

**Fig. 64:**
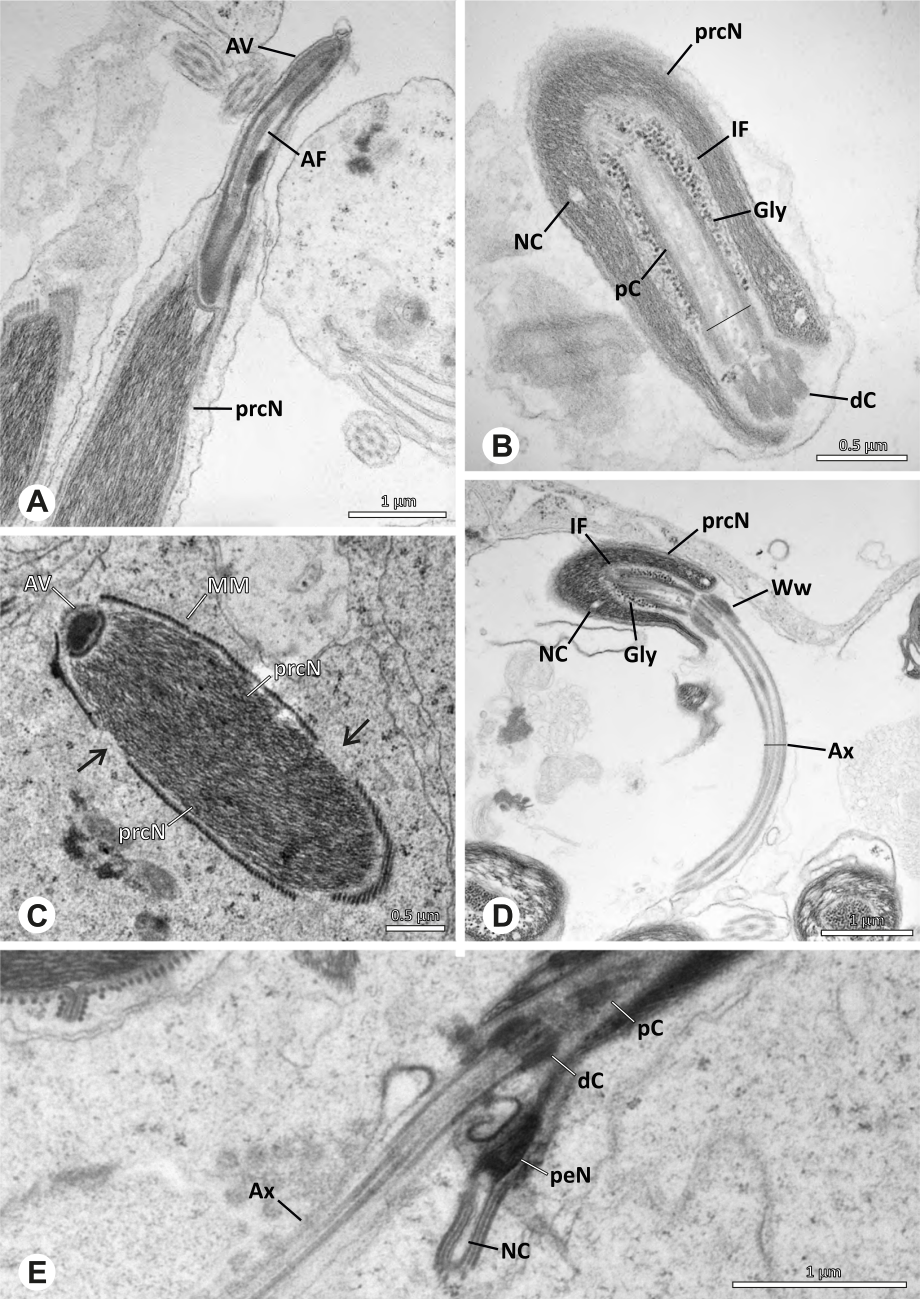
Spermiogenesis of *Pholcus* spp. **A** Mid spermatid of *P. opilionoides*. Note the cylindrical AV. **B** Mid spermatid of *P. opilionoides*. Note the prolonged proximal centriole and the IF filled with glycogen. **C** Mid spermatid of *P. guineensis*. Note the gaps in the manchette of microtubules (arrows). **D** Late spermatid of *P. opilionoides*. The nuclear canal is shifted to the periphery of the prcN as soon as it meets the implantation fossa. **E** Late spermatid of *P. guineensis*, longitudinal section. Note the short peN as well as the electron dense material surrounding the distal centriole

**Fig. 65:**
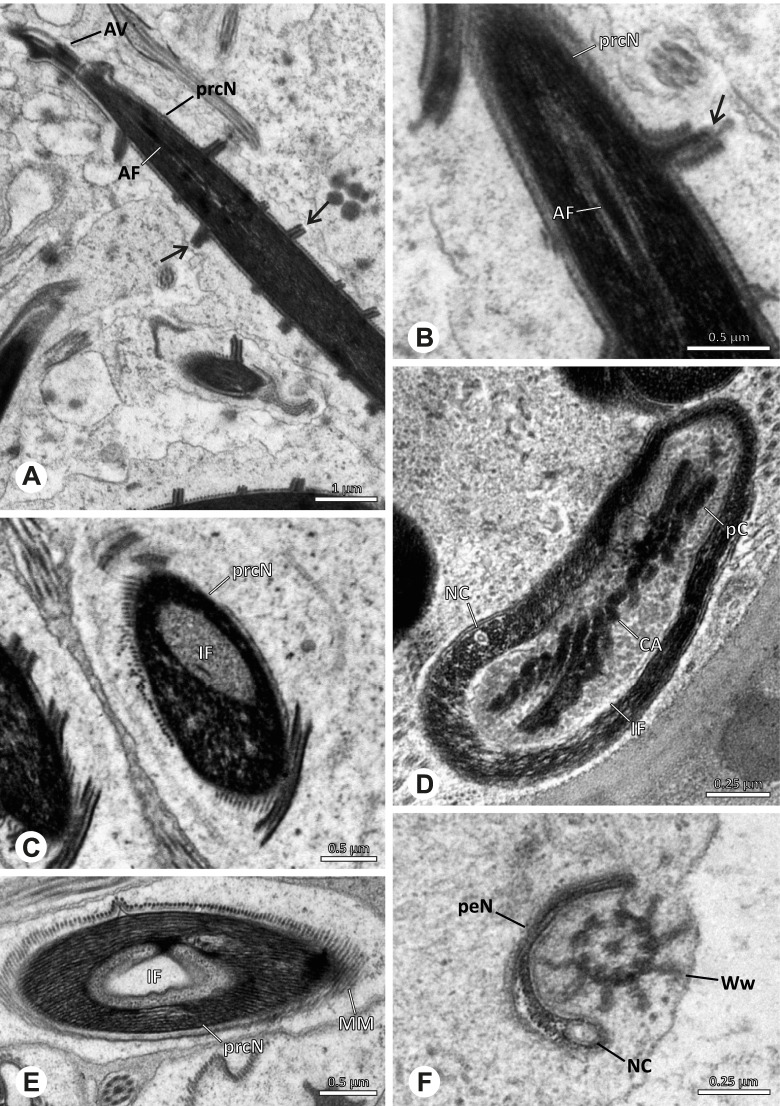
Spermiogenesis of *Pholcus* spp. **A** Late spermatid of *P. kindia*, longitudinal section. Note the cylindrical AV as well as the prominent helical band of nuclear material (arrows). **B** Late spermatid of *P. kindia*. Note the helical band (arrow) and the centrally running nuclear canal with the acrosomal filament. **C** Late spermatid of *P. attuleh*., cross-section. The IF is filled with glycogen. **D** Late spermatid of *P. bamboutos*. The IF is filled with glycogen-like material and the pC is in contact with electron dense filamentous anterior centriolar adjunct material. **E** Late spermatid of *P. bamboutos*, cross-section. The IF is peripherally filled with glycogen-like material. **F** Coiled spermatid of *P. kindia*, close-up. Note the electron dense material in “water wheel” configuration

**Fig. 66:**
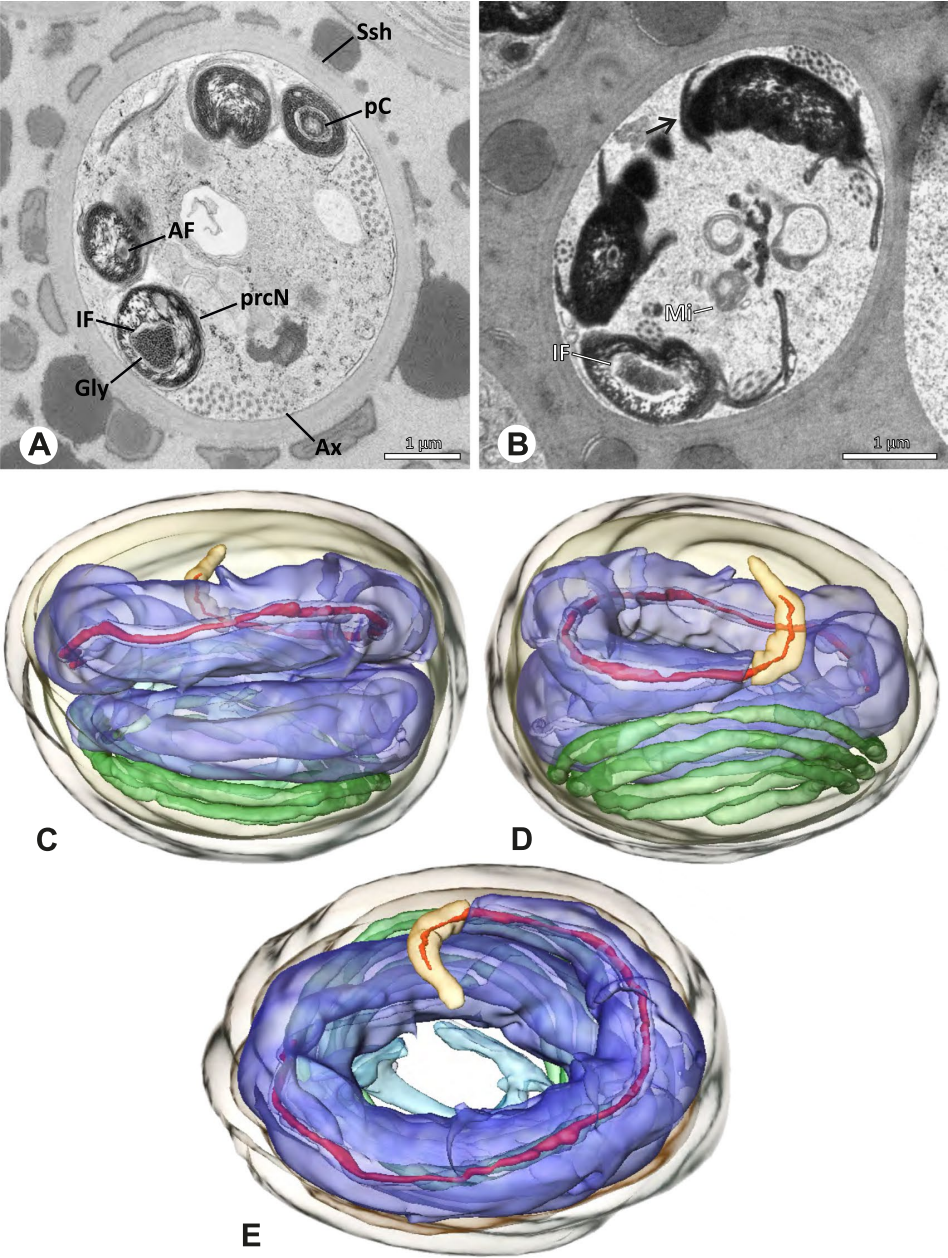
Ultrastructure and 3D surface reconstruction of the cleistospermia of *Pholcus* spp. **A** Cleistosperm of *P. opilionoides*, cross-section. Note the IF filled with glycogen. **B** Cleistosperm of *P. bamboutos*. Note the helical band of nuclear material along the nucleus (arrow). **C**, **D**, **E** 3D surface reconstruction of a cleistosperm of *P. bamboutos*, representing the configuration for the studied representatives of the genus. Note the central nuclear canal

**Spermatozoa.** Acrosomal complex. Cylindrical to conical, subacrosomal space extends throughout the entire AV (Fig. 64A). AF stout, extends into the NC through about half of the prcN. Nucleus. prcN tubular and nearly radially symmetric (Fig. 65A), with prominent helical band of nuclear material (Fig. 65A, B arrows). NC runs centrally through the prcN (Fig. 65B) and shifts into the periphery within the peN (Figs. 64D, E and 65F). IF occupies about half of the prcN and is filled with glycogen [*P. opilionoides* (Schrank, 1781); *P. kindia* Huber, 2011; Figs. 64B, 65C and 66A] or granular, glycogen-like material (*P. attuleh* Huber, 2011; *P. bamboutos* Huber, 2011; Figs. 65E and 66B). Centrioles in contact with electron dense material in “water wheel” configuration (Figs. 65F and 64D), proximal centriole notably elongated (Fig. 64B). Electron dense fibrillar centriolar adjunct material restricted to the IF present in *P. bamboutos* (Fig. 65D). peN short and flat (Fig. 66E). Chromatin condensation homogenous.

**Sperm transfer form.** Spherical cleistospermia surrounded by a secretion sheath (Fig. 66A, B). Nucleus spirally coiled (Fig. 66E), axoneme coiled three to five times beside the nucleus (Fig. 66D). Cytoplasm heterogenous, mitochondria present (Fig. 66B).

**Notes on Spermiogenesis.** Early spermatids are characterized by a centrally situated NC and gaps in the MM (Fig. 64C arrows). In mid to late spermatids, a prominent helical band develops (Fig. 65A arrows). The content of the IF as well as the material surrounding the centrioles in “water wheel configuration” only appear in late spermatids, together with the developing anterior centriolar adjunct material, if present.

**Seminal secretions.** *Pholcus bamboutos:* One type of secretion, round and homogenous (Fig. 5B). *P. kindia:* Two types of secretions, one homogenously electron dense, the other compact and granular (Fig. 5C). *P. opilionoides:* Two types of secretions, both irregularly shaped but of different electron densities (Fig. 6C). *P. attuleh*: One type of secretion; round to curved, homogenous margin with scattered electron lucent patches in the center.

### Pholcinae | *Quamtana oku* Huber, 2003 (Figs. 67 and 8)

**Fig. 67:**
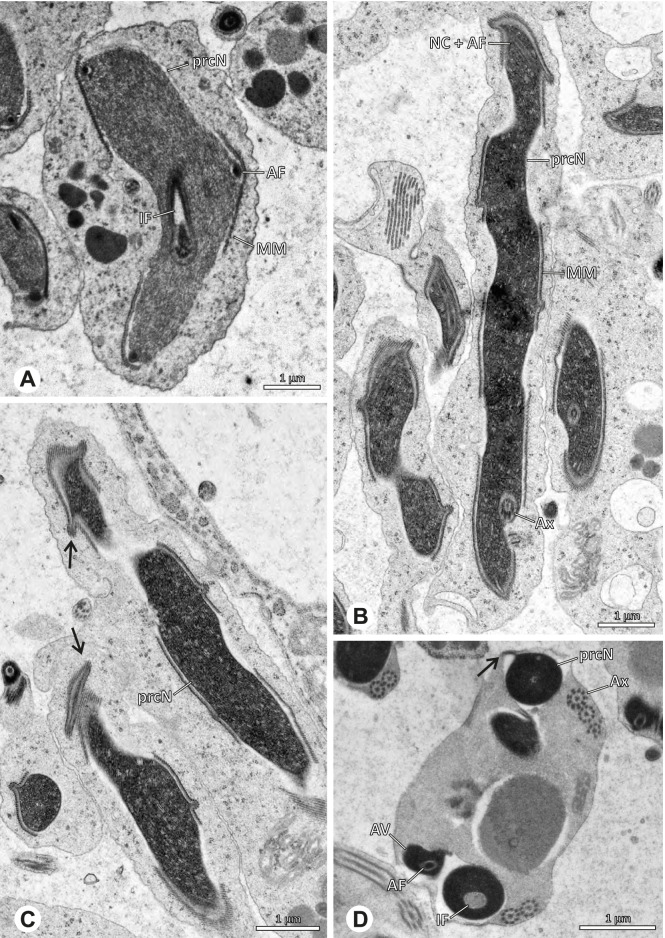
Spermiogenesis of *Quamtana oku*. TEM. **A** Mid spermatid. Note the gaps in the manchette of microtubules. **B** Late spermatid. The gaps in the manchette of microtubules are prominent at this stage. Note the position of the NC and AF. **C** Late spermatid. A helical band of nuclear material is present along the nucleus (arrows). **D** Coiled spermatid. The helical band remains present also after coiling (arrow)

**Fig. 68:**
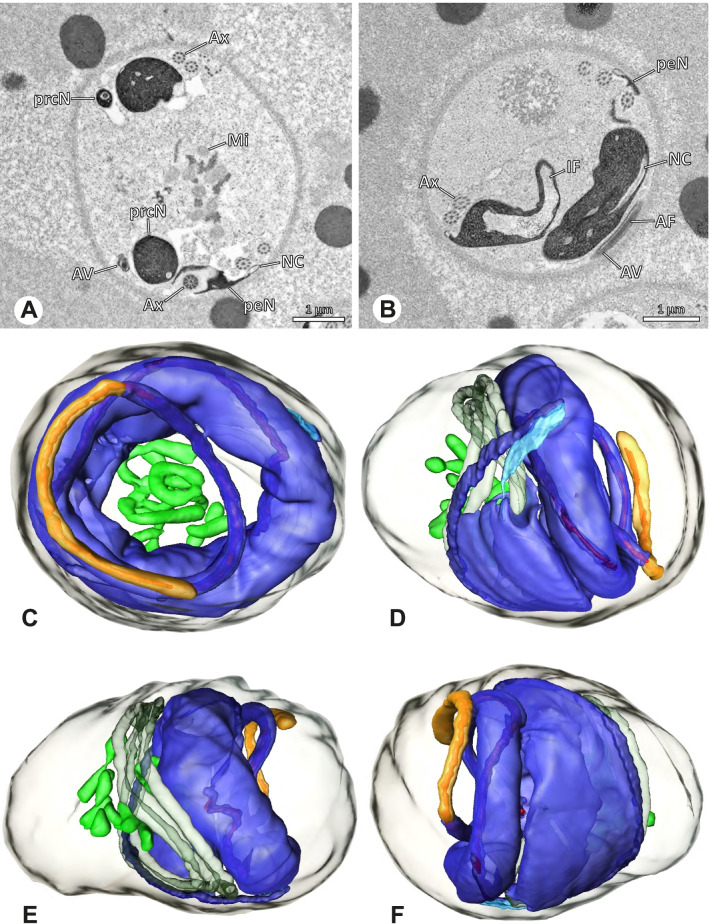
Ultrastructure and 3D surface reconstruction of the cleistospermia of *Quamtana oku*. TEM. **A**, **B** Cleistospermia in the lumen of the deferent duct. Note the variation in diameter of the prcN. **C**, **D**, **E**, **F** 3D surface reconstruction of a cleistosperm. Note the almost filiform anterior part of the prcN

**Spermatozoa.** Acrosomal complex. AV long and cylindrical with subacrosomal space extends throughout the entire AV (Fig. 68C). AF stout, extends halfway through the prcN (Fig. 68C, D). Nucleus. prcN nearly radially symmetric (Fig. 67D) and cylindrical, becomes very thin, almost filiform in its anterior part (Fig. 68C). NC runs centrally through the thin anterior part of prcN and laterally through the remaining part of the prcN and peN (Fig. 68B, C, D). IF shallow (Fig. 68B). peN narrow and flat to triangular in cross section (Fig. 68B, D). Chromatin condensation homogenous.

**Sperm transfer form.** Spherical cleistospermia, surrounded by a secretion sheath (Fig. 68A, B). prcN and peN spirally coiled, AV bent on top of prcN (Fig. 68C, D, F). Ax coiled three times beside the nucleus (Fig. 68E). Cytoplasm heterogenous, mitochondria present (Fig. 68A).

**Notes on Spermiogenesis.** In mid spermatids, the nuclear surface starts to form lateral protrusions (Fig. 67A). A rather inconspicuous helical band develops in late spermatids (Fig. 67C).

**Seminal secretions.** One type of secretion, globular and loosely scattered, homogenously electron dense (Fig. 5D).

### Pholcinae | *Spermophora senoculata* (Dugès, 1836) (Fig. 69)

**Fig. 69:**
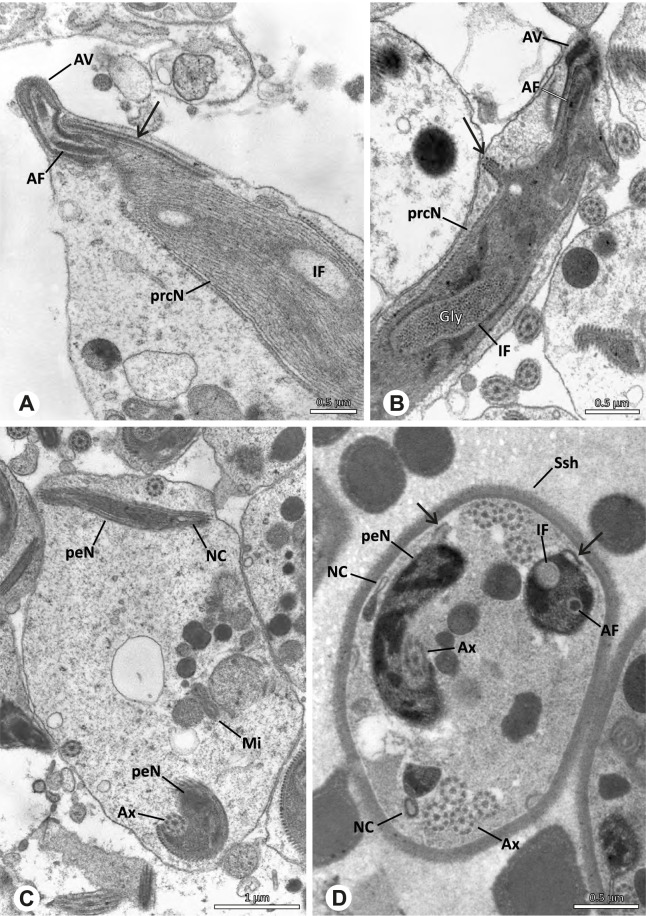
Spermiogenesis and cleistosperm of *Spermophora senoculata*. TEM. **A** Early to mid spermatid. Note the depth of the IF and the electron dense plate besides the prcN (arrow). **B** Mid to late spermatid, longitudinal section. Note the presence of a helical band of nuclear material along the prcN (arrow) and the IF filled with glycogen. **C** Coiled spermatid. Note the peripheral position of the NC along the peN. **D** Cleistosperm in the lumen of the deferent duct. The helical band is still present at this stage (arrow)

**Spermatozoa.** Acrosomal complex. AV cylindrical, subacrosomal space narrow (Fig. 69A). AF stout, extends into the NC and ends before the axonemal basis. Nucleus. prcN slender, cylindrical, nearly radially symmetric, IF deep and filled with glycogen (Fig. 69B). Helical band of nuclear material present along the prcN (Fig. 69B arrow). peN partially enclosing the Ax while winding around it (Fig. 69C, D). NC projecting centrally through the anteriormost part of the prcN (Fig. 69B) and shifted into the periphery along the peN (Fig. 69C, D).

**Sperm transfer form.** Spherical to oval cleistospermia surrounded by a secretion sheath, Ax coiled four times around the nucleus; cytoplasm heterogenous, electron dense, secretions and mitochondria present (Fig. 69D).

**Notes on spermiogenesis.** In early to mid spermatids, the anterior pole of the nucleus and the base of the ACV are flanked by a thin, electron-dense plate (Fig. 69A arrow), the MM shows partial gaps and the IF appears empty (Fig. 69A). Mid to late spermatids develop a prominent helical band along the prcN, the IF fills with glycogen (Fig. 69B).

**Seminal secretions.** One type of secretions, roundish with electron dense center and less dense margin (Fig. 6B).

### Pholcinae | *Spermophora awalai* Huber, 2014 (Figs. 70 and 71)

**Fig. 70:**
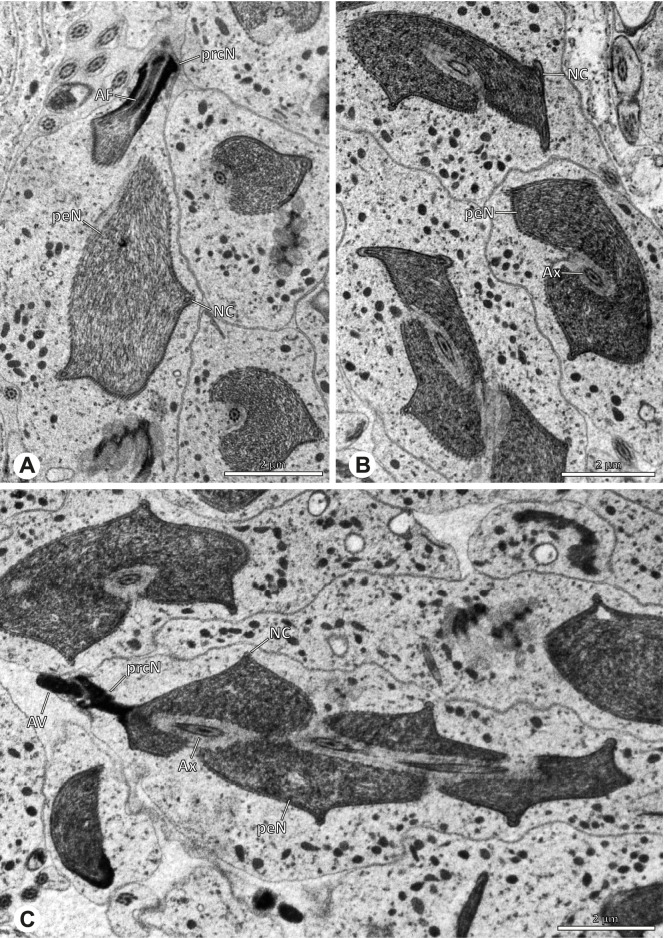
Spermiogenesis of *Spermophora awalai*. TEM. **A** Mid to late spermatid. Note the short prcN. **B** Late spermatid. The peN is twisting around the axoneme. **C** Late spermatid, longitudinal section. Note the short prcN, the long peN twisting around the axoneme and the elevated position of the NC within the peN

**Fig. 71:**
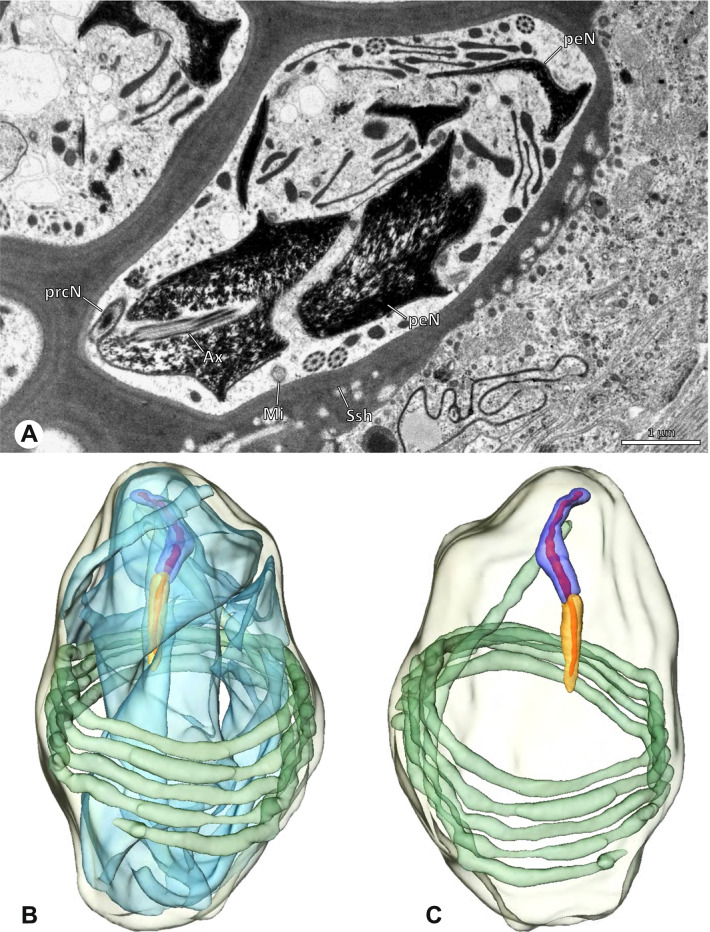
Ultrastructure and 3D surface reconstruction of the cleistospermia of *Spermophora awalai* (**A**) Cleistosperm in the lumen of the deferent duct. **B**, **C** 3D surface reconstruction of a cleistosperm. Note the elongated peN (**B**). The peN is omitted for the purpose of clarity in (**C**)

**Spermatozoa.** Acrosomal complex. AV cylindrical, subacrosomal space extends throughout the entire AV (Fig. 71C). AF extends into the NC until the axonemal basis (Fig. 71B). Nucleus. prcN much shorter than the peN, nearly radially symmetric, tubular (Fig. 71A, C). IF small. peN long and voluminous, with various thin extensions (Figs. 70C and 71A, B), twisted around the axoneme resulting in a helical appearance (Fig. 70C). NC runs centrally through the prcN and shifts to a lateral position within the extensions of the peN (Figs. 70C and 71C).

**Sperm transfer form.** Oval cleistospermia, surrounded by a secretion sheath (Fig. 71A). Nucleus bent and twisted around itself, with the short prcN as well as the AV resting centrally within the cleistosperm (Fig. 71B, C). Ax coiled five times centrally around the peN (Fig. 71B). Cytoplasm with secretions; mitochondria present (Fig. 71A).

**Notes on spermiogenesis.** In mid and late spermatids, the NC is located in the lateral extensions of the long peN (Fig. 70A). The manchette of microtubules is present on the outer surface of the peN, but not on the inner side near the Ax (Fig. 70A, B). The chromatin condensation is homogenous.

**Seminal secretions.** Two types of secretions present, one elongated and electron lucent, the other roundish with center more electron dense than margin (Fig. 5E).

### Sperm size and minimum diameter of spermophor

**Fig. 72:**
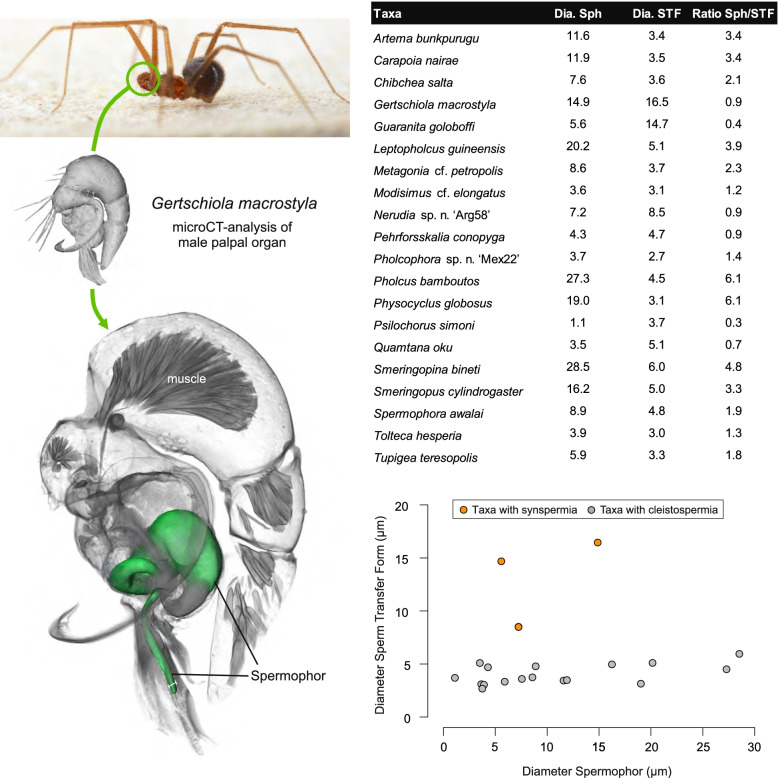
Minimum diameters of sperm and of the spermophor in the male genital bulbs in Pholcidae. Minimum diameters of spermophor were obtained using micro-CT data (illustrated here with *Gertschiola macrostyla*) and the minimum diameter of the sperm using TEM data. The scatter plot shows that there is no obvious correlation between the diameters of sperm and spermophor

The diameter of the thinnest portion of the spermophor as well as the dimension of sperm transfer forms were measured based on image data from micro-computed tomography (spermophor) and TEM (transfer forms) for 20 species representing the five subfamilies (Fig. 72). Species with cleistospermia possess relatively small transfer forms with a diameter ranging from 2.7 µm (*Pholcophora* sp. n. ‘Mex22’), to approximately 5 µm (*Quamtana oku* 5.1 µm, *Leptopholcus guineensis* 5.1 µm, *Smeringopus cylindrogaster* 4.9 µm), while the minimum diameter of the according spermophor varies greatly from 1.1 µm (*Psilochorus simoni*) to 28.5 µm (*Smeringopina bineti*). In species with synspermia, transfer forms were much larger with diameters between 8.5 µm (*Nerudia* sp. n. ‘Arg58’) and 16.5 µm (*Gertschiola macrostyla*). However, the diameter of the according spermophor was in the range of those for species with cleistospermia.

## Discussion

The present study is the first to provide a detailed comparative analysis of sperm morphology of pholcid spiders. We unraveled a remarkable diversity throughout this family and will discuss our results in the light of their evolutionary, functional and phylogenetic implications.

### Evolutionary implications

#### Acrosomal complex

The acrosomal complex of spider spermatozoa comprises the acrosomal vacuole with a posterior invagination, and the subacrosomal space which contains electron dense material that differentiates into the acrosomal filament during early spermiogenesis (e.g. [28]). Especially the acrosomal vacuole varies in shape among spiders [22], but it is rather uniform across the studied pholcids. The acrosomal filament consists of F-actin fibrils [44] and projects into the nuclear canal – the length of the acrosomal filament, however, varies among spider taxa [22]. In pholcids, an acrosomal filament that ends before the axonemal basis seems to be characteristic for the family, with few reversions to a longer filament in the subfamily Modisiminae (*Carapoia, Otavaloa, Psilochorus*). The functional role of the acrosomal filament in spiders is not known. As it is involved in the fertilization process in other animal taxa (e.g. [46, 47]), such a function is plausible in spiders as well. However, the exact processes of fertilization in spiders are unknown. Alberti [28] hypothesized that the acrosomal filament influences the definite shape of the nucleus during spermiogenesis. This hypothesis is also supported by the findings of Lipke and Michalik [48] for the caponiid spider *Caponina alegre*, where the nucleus is directionally twisting around a central acrosomal filament.

#### Nucleus

*Microtubules in the implantation fossa during spermiogenesis. *The development of the implantation fossa begins already at early stages of spermiogenesis, as it coincides with the chromatin condensation [21, 30]. The implantation fossa can contain various materials [Fig. 12 in 22], whose functions are largely unexplored. Previously, the presence of microtubules inside the implantation fossa during spermiogenesis has been reported only for the oonopid genus *Orchestina* [31] and the pholcid *Holocnemus pluchei* (Smeringopinae) [19, 20]. In both taxa, the microtubules are later displaced, either by centriolar adjunct material (*Orchestina*) or by glycogen (*Holocnemus*). Our analyses suggest that the presence of microtubules during spermiogenesis is characteristic for the subfamily Smeringopinae. However, a displacement of the microtubules by centriolar adjunct material or glycogen in mature spermatozoa could not be observed in all investigated taxa. *Smeringopus* cf. *roeweri* showed distinct electron dense filamentous material in the implantation fossa of mature sperm (Fig. 48A, B), which however was already present besides the microtubules in late spermatids [Fig. 5G in 22]. The function of microtubules during spider spermiogenesis is unclear, but it has been suggested that they might play a role in the formation of the nucleus [22]. This might be especially important for taxa with elongated nuclei and a deep implantation fossa where a stabilizing function is plausible. Our data support this hypothesis, as a deep implantation fossa is characteristic for Smeringopinae.

**Fig. 73:**
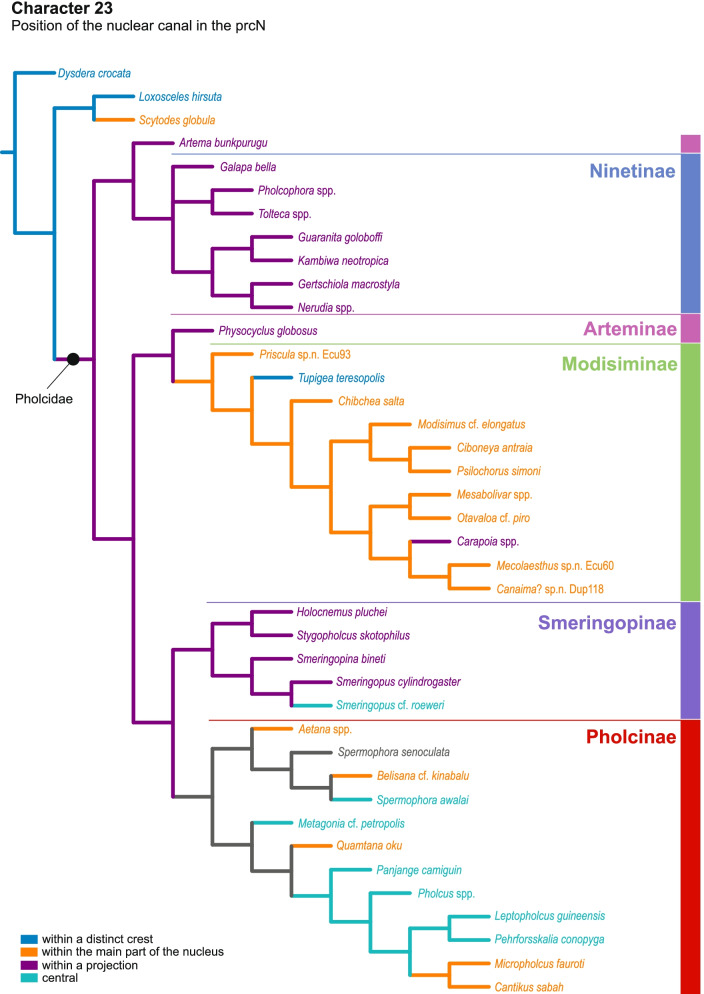
Scenario of the evolution of the position of the nuclear canal (Character 23) across the pholcid tree of life. Character tracing using unambiguous optimization obtained with WinClada [50], phylogeny based on Huber et al*.* (2018) [7]

*Nuclear canal.* As mentioned above, the acrosomal filament extends into the nuclear canal. The position of the nuclear canal relative to the nucleus can be manifold, especially with regard to the precentriolar part [22, 28]. Our analysis revealed that in Pholcidae, all previously described configurations of the nuclear canal can be observed. The position of the nuclear canal in a distinct projection along the precentriolar portion of the nucleus (Char. 23:1) appears to be characteristic for Ninetinae and Smeringopinae, with the exception of *Smeringopus* cf. *roeweri*, where the nuclear canal appears to run centrally within the nucleus (Fig. 48B). The nuclear canal projecting centrally through the precentriolar portion of the nucleus has previously been described only for *Pholcus phalangioides* by Alberti and Weinmann [16] and by Michalik and Uhl [17]. The term “central” here refers to the position of the nuclear canal before being displaced posteriorly to the periphery by the implantation fossa. Our analysis revealed that the central nuclear canal is characteristic for certain ‘basal’ pholcine species (Fig. 73) such as *Metagonia* cf. *petropolis* and *Spermophora awalai* and most abundant in “Pholcinae group 3” sensu Huber, Eberle and Dimitrov [7], as it is present in *Panjange camiguin*, *Pehrforsskalia conopyga*, *Leptopholcus guineensis* and several species of *Pholcus*. The central position of the nuclear canal in these pholcine taxa might be related to the general morphology and development of the nucleus, as the acrosomal filament might play a role in nuclear shaping as it has been suggested for *Caponina alegre* (see above). Therefore, a central position of the acrosomal filament can have a significant influence on the deviation from the usually asymmetric shape of spermatids in spiders [22, 49], leading to the impression of a nearly radially symmetric nucleus in many Pholcinae.

Another noteworthy configuration is present in *Tupigea teresopolis*, where the nuclear canal is located within a distinct crest (Char. 23:2) (Fig. 43A). This configuration has previously been reported for *Dysdera* sp. (Dysderidae) [16] and *Loxosceles hirsuta* [22]. Such a position within a distinct projection could benefit the development of a more slender shape of the nucleus (e.g. Figs. 45 and 47). In Ninetinae, a compacting of the nucleus due to the position of the nuclear canal could be an adaptation for space-efficient arrangement of sperm cells into synspermia (see also below).

**Fig. 74:**
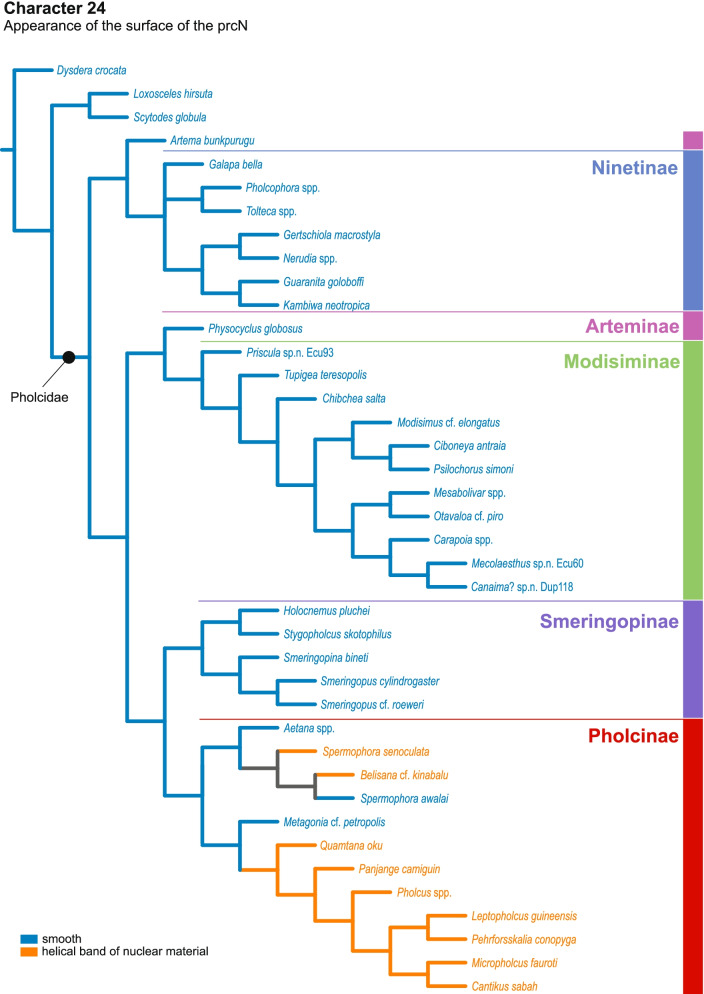
Scenario of the evolution of the appearance of the surface of the precentriolar portion of the nucleus (Character 24) across the pholcid tree of life. Character tracing using unambiguous optimization obtained with WinClada [50], phylogeny based on Huber et al*.* (2018) [7]

*Appearance of the precentriolar part of the nucleus.* The precentriolar part of the nucleus is known to have various modifications in spiders, regardless of an association with the nuclear canal [22]. Those modifications range from longitudinal ridges (Char. 41:3 in Lipke and Michalik [31]; Char. 24:2 in Michalik and Ramírez [22] to specific extensions of nuclear material surrounding the nucleus and forming a helical band [16, 17]. The latter has been considered to be a specific character within Pholcidae with possible phylogenetic implications as it was observed in several taxa within the subfamily Pholcinae [17, 22]. Our data confirm the presence of a helical band in numerous taxa within Pholcinae being characteristic at least for parts of this subfamily (Fig. 74). According to Alberti and Weinmann [16], the helical band develops during spermiogenesis due to gaps in the manchette of microtubules surrounding the nucleus, which we also observed in all taxa with a helical band (e.g. Fig. 67). In contrast, the pholcine *Spermophora awalai* does not possess a helical band, as in fact the prominent extensions of the nucleus shown in Figs. 69 and 70 are part of the postcentriolar elongation of the nucleus while the precentriolar part is very reduced (Figs. 70C and 71C). This conspicuous absence of a helical band in combination with a shortened precentriolar part of the nucleus can also be observed in other genera within Pholcinae such as *Aetana* and *Metagonia* (Figs. 50D and 58). We hypothesize that the occurrence of a helical band might be connected to the presence of a sufficiently long precentriolar portion of the nucleus. The function of the helical band is not known, but Michalik and Uhl [17] considered it to be involved in fertilization or to influence sperm motility.

*Shape of the nucleus – the special case of Aetana.* Alberti [28] described the development of the postcentriolar elongation of the nucleus as starting from one side of the posterior margin of the implantation fossa and subsequently coiling around the axoneme as it elongates, resulting in an asymmetric appearance of the nucleus. In the genus *Aetana*, the posterior elongation begins to coil around the axoneme in early spermatids, but fuses completely during maturation resulting in a tube-like hollow appearance (Fig. 51). Furthermore, the peN is extremely elongated in relation to the precentriolar part of the nucleus, a configuration which can also be found in *Spermophora awalai* (Pholcinae). However, the latter shows a peN that is tightly coiled around the axoneme but lacks the ultimate fusion of the coils and does therefore not form a closed tube. Comparable configurations have been described for other spider taxa as well: In several tetragnathid species, spermatids exhibit a short precentriolar part of the nucleus together with a very long postcentriolar elongation, which twists around the axoneme but ultimately does not fuse like in *Aetana* [51]. The caponiid spider *Caponina alegre* also exhibits a twisted nucleus, but there, the mechanism of directional nuclear twisting differs as the precentriolar portion of the nucleus winds around the central acrosomal filament [48].

In the case of *Aetana*, the peN contains the axoneme and the centriolar adjunct material, which could potentially have an impact on sperm motility (see also below). Moreover, it was shown for various other spider taxa, that the length of the peN is positively correlated with the length of the axoneme [31, 33, 48]. This is also the case in *Aetana*, especially when considering that the peN encloses a substantial portion of the axoneme (Fig. 51A), which adds to a hypothesized functional role in motility. From an evolutionary perspective, the length of the axoneme and therefore also the peN in spiders might be influenced by postcopulatory sexual selection. An analogous phenomenon has been suggested for insects where sperm morphology is positively correlated with the complexity of the female genital tract [52].

#### Axoneme

*Centriolar adjunct material.* The centriolar adjunct material shows a remarkable variation among pholcids, ranging from uniformly electron dense material expanding into the implantation fossa (Char. 47:0), for example in *Panjange camiguin* (Fig. 61A), to more complex organizations posterior of the axonemal basis like in *Mesabolivar* spp. (Fig. 36C, D). The presence of centriolar adjunct material seems to be a derived state in pholcids, since the early branching subfamilies Ninetinae and Arteminae do not show this structure.

With regard to the organization of the centriolar adjunct, the subfamily Modisiminae stands out as most studied species have a collar-like arrangement of dense lamellae around the anterior part of the axoneme. This lamellated organization of the posterior centriolar adjunct is not known from any other spider group with the exception of the pholcine *Pehrforsskalia conopyga*, where it is less extensive in size compared to most of the investigated Modisiminae (Fig. 63A). The function of the centriolar adjunct is unknown for spider spermatozoa. Centriolar adjunct material has been reported for other animal taxa such as mammals [53, 54] and birds [55]. In insects, the centriolar adjunct material is associated with the so-called pericentriolar material (PCM), an electron dense aggregation in contact with the centrioles, as it is built of similar proteins such as γ-tubulin [56, 57]. Since the PCM is considered to be related to the development of the manchette of microtubules surrounding the nucleus during spermiogenesis, a functional relation of the centriolar adjunct material to nuclear shaping has been hypothesized in insects [58], and also mammals [59]. Another possible role of the centriolar adjunct material is related to sperm motility. Dallai, Paoli, Mercati and Lupetti [58] suggested that the centriolar adjunct could support the connection between nucleus and axoneme, thereby acting as a structure for accumulation of mechanical energy, leading to a more uniformly beating axoneme. Such a functional comparison to a mechanical spring, as well as a connection to the motility of the axoneme in general, could as well be considered for spiders with centriolar adjunct material located posterior of the centrioles. For example, the centriolar adjunct material in *Mesabolivar* spp. does not only resemble a layered spring in longitudinal section (Fig. 36F), but also consists of one lamella per microtubule doublet (Fig. 36G), suggesting a functional correlation between centriolar adjunct and axoneme. Michalik, Aisenberg, Postiglioni and Lipke [60] hypothesized that the length of the posterior centriolar adjunct material might be positively correlated with the depth of the implantation fossa in spiders of the RTA-clade. Our study supports this hypothesis for Pholcidae, as several Modisiminae investigated in this study show a short implantation fossa and a centriolar adjunct material accompanying the axoneme along approximately two thirds of its length (Figs. 36 and 30).

#### Transfer forms

**Fig. 75:**
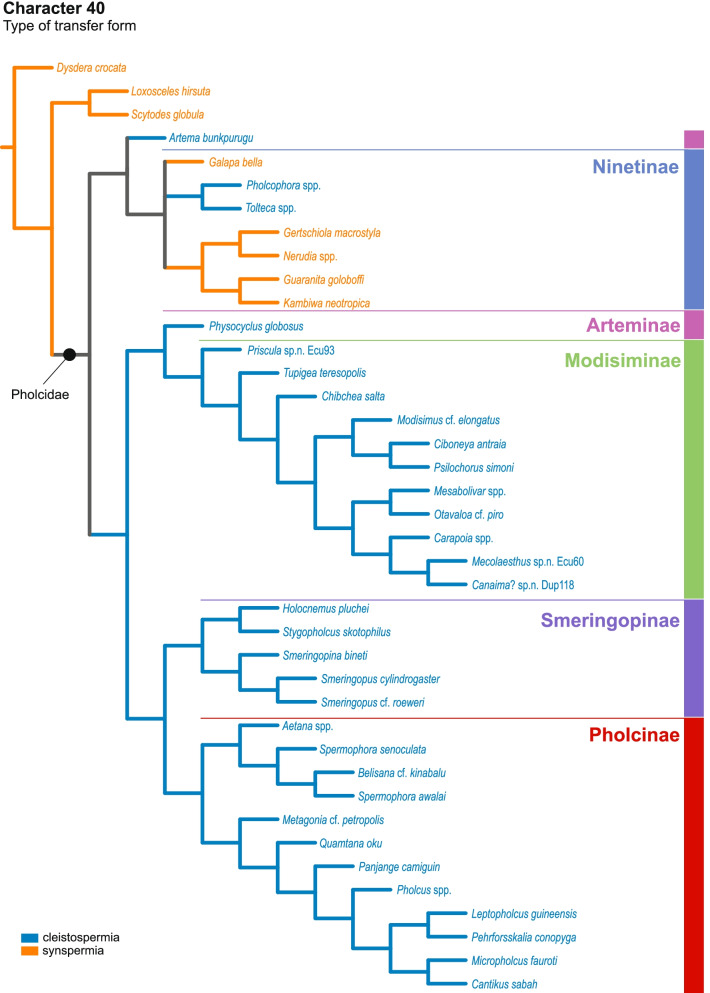
Scenario of the evolution of the type of sperm transfer form (Character 40) across the pholcid tree of life. Character tracing using unambiguous optimization obtained with WinClada [50], phylogeny based on Huber et al*.* (2018) [7]

Spiders transfer sperm either individually (cleistospermia) or conjugated (multiple sperm physically united). Synspermia are considered primary conjugates as sperm cells within each conjugate stem from one spermatocyte, whereas in contrast, secondary conjugates (coenospermia, rouleaux) form after separation of spermatids [22, 61]. Except for most Ninetinae, which transfer synspermia (Char. 40: 2), all other studied pholcids transfer cleistospermia (Char. 40:1) (Fig. 75). Within Ninetinae, a transformation from synspermia to cleistospermia (in the genera *Pholcophora* and *Tolteca*) took place (see also below). The occurrence of different types of transfer forms within the same family has been previously reported for example in Oonopidae [16, 22, 31].

In oonopids, the mechanism of formation of transfer forms showed certain particularities, such as an initial fusion of spermatids during spermiogenesis, before spermatids individualize later to ultimately form cleistospermia [16]. Strong indications for the initial fusion of multiple spermatids in early stages of spermiogenesis are also observable in several pholcids which transfer cleistospermia, like *Carapoia nairae* (Fig. 9A), *Chibchea salta* (Fig. 9B), *Mesabolivar* spp. (Fig. 9C, D)*, Smeringopus* cf. *roeweri* (Fig. 9E)*, Holocnemus pluchei* (Fig. 9F) *and Pholcophora* spp*.* (Fig. 21A). The mechanism observable in *Pholcophora* spp. seems comparable to that observed in oonopids. On the other hand, the formation of synspermia in ninetines is more similar to the descriptions of Michalik, Dallai, Giusti and Alberti [45] (Dysderiidae), or, partially, to those of Costa-Ayub and Faraco [44] (Sicariidae). It seems that, within Synspermiata, a reversion to cleistospermia, if occurring, generally follows a comparable mechanism where the initial fusion of developing spermatids is not necessarily a definitive indicator for the final transfer form.

Within Ninetinae, synspermia vary considerably in their organization. For example, the number of fused spermatids ranges from 16 (*Nerudia, Kambiwa*) to 64 (*Galapa*). Such intraspecific variation is known also from other spider families, for example Oonopidae [31], Caponiidae [48] and Orsolobidae [33], as well as from insects [62, 63].

Sperm conjugates are known to be often equipped with various internal membranes [16, 31], which have been hypothesized to be involved in sperm activation. Since spiders transfer sperm in an inactive coiled state, a re-activation within the female after copulation is mandatory [42, 64]. The exact activation processes are not unraveled, but they may have crucial implications for post-copulatory sexual selection [65]. A conspicuous organization of internal membranes is the so-called vesicular area, which has been described for several taxa with synspermia (e.g. [16, 45, 48]) but only for two species with cleistospermia, *Oonops domesticus* Dalmas, 1916 (Oonopidae) and *Psilochorus simoni* (Modisiminae) [16, 21]. Our data show the presence of a vesicular area in two further Modisiminae species with cleistospermia: *Modisimus elongatus* (Fig. 38D) and *Canaima*? sp. n. ‘Dup118’ (Fig. 29C). *Priscula* sp. n. ‘Ecu93’ has a distinct membranous area surrounding parts of the axoneme, which, however, lacks the electron density of a vesicular area (Fig. 41C). The presence of a vesicular area is known also from various other arachnids such as Ricinulei [66]. The exact function of the vesicular area has not been resolved. However, hypotheses include an involvement in the capacitation (e.g. uncoiling) process in the female genital tract, as it has been described for example for anactinotrichid mites [67]. This “precapacitation” [16] seems especially beneficial for taxa with synspermia as hypothesized by Costa-Ayub and Faraco [44].

Sperm transfer forms in spiders have a secretion sheath that encapsulates the spermatozoa (Char. 38:0) (e.g. [28]). Exceptions are only known from a few species of Caponiidae [48], Orsolobidae [33], Theridiidae [68], and Oonopidae [31]. Functionally, a protective purpose of the secretion sheath has been postulated [28], which can also be related to sperm storage in the female after copulation [60]. Unsheathed sperm however, could be an adaptation to postcopulatory processes, as the activation of sperm includes decapsulation, i.e. dissolving the secretion sheath initiated by the female [64, 69]. Unsheathed sperm have therefore been suggested to “bypass” this step in the sperm activation process [33]. It was further suggested in that study that the protective function of the secretion sheath should be taken over by different structures such as specific seminal secretions. Our data revealed the presence of unsheathed synspermia in *Galapa bella*. Here, a functional replacement for the missing secretion sheath could be the tile-like secretions in the seminal fluid, which densely surround the synspermia (Fig. 12D). The embedding of transfer forms in a matrix of secretions for putative protective purposes has also been described for other Synspermiata, such as the genus *Oonops* (Oonopidae) [31]. A specific case can be found in the synspermia of *Kambiwa neotropica*. Here, the sheathed synspermia contain spermatozoa that are surrounded by a matrix of secretions as well as internal tile-like membranous structures (Fig. 18). A protective function can be assumed for the secretion sheath, but the internal secretions and structures might serve the same or different purposes possibly related to sperm activation and sperm storage. For example, we speculate that if decapsulation and unpacking of synspermia are processes strongly separated from each other in time, the secretion in the synsperm may take over the function of the secretion sheath.

*Sperm transfer form size.* The size of sperm transfer forms in Pholcidae varies greatly among species, especially due to the presence of different types of transfer forms (i.e., synspermia and cleistospermia). Most strikingly, the synspermia of Ninetinae can be about four times larger than the cleistospermia of Pholcinae (Fig. 72: Sperm transfer form (STF) diameter: *Gertschiola macrostyla*: 16.5 µm, *Pholcus bamboutos*: 4.5 µm). Also, the shape and therefore the length of transfer forms vary remarkably among the investigated pholcid taxa: while most cleistospermia are nearly spherical, synspermia can expand enormously in length and measure up to more than five times the length of certain artemine cleistospermia (e.g. *Galapa bella*: 19.1 µm, *Artema bunkpurugu*: 3.3 µm). As mentioned above, all investigated ninetine species transfer synspermia, except for *Pholcophora* and *Tolteca*, which transfer cleistospermia. Their cleistospermia do not differ much in size from those of other pholcids (Fig. 72: *Tolteca*: 3.0 µm, *Pholcophora*: 2. 7 µm, *Tupigea*: 3.3 µm, *Pholcus* 4.5 µm), but the minimum diameters of the spermophors range among the smallest of all studied taxa (Fig. 72: *Tolteca*: 3.9 µm; *Pholcophora*: 3.7 µm).

Previous studies suggested a correlation between STF and spermophor diameter [30–32]. In Pholcidae we found considerable variation in both the diameter of transfer forms and the minimum diameter of the spermophor but we did not find an obvious correlation between the two variables. Both STF as well as the spermophor are flexible and elastic to a certain extent and our measures (in particular those of spermophor diameter) are based on single specimens only. Thus, our measures are not precise, explaining ratios below 1. Furthermore, we cannot exclude the possibility that transfer forms compactify in the further course of sperm transfer and storage. We believe that the general conclusion still holds because of the number of species measured, but this aspect clearly needs further study with increased sample sizes.

The variability of transfer form size could potentially be driven by the morphology of the female genitalia and by post-copulatory sexual selection, as described for nephilid spiders [70] and for insects (e.g. [35, 71]). As sperm mixing reportedly occurs in *Pholcus phalangioides* [15], the influence of sperm competition on the size of sperm transfer forms has to be considered. Our data suggest that in Ninetinae, only one transfer unit can pass through the thinnest portion of the spermophor at a time, regardless of the kind of transfer form that is used (i.e., synspermia or cleistospermia) (Fig. 72 “Ratio”). In contrast, for most of the remaining Pholcidae, up to approximately six cleistospermia can pass at once (e.g., *Pholcus bamboutos*, *Physocyclus globosus*). Nevertheless, the transfer of one synsperm at a time, even of those containing the fewest spermatozoa (e.g., *Nerudia*: 16), transports more sperm cells than the combined number of cleistospermia do in the case of *Pholcus bamboutos* or *Physocyclus globosus* per time unit. This makes an inclusion of individual copulation duration across pholcid species necessary to allow for further hypotheses. However, data on reproductive behavior, especially in Ninetinae, are largely lacking. This does not take into account potential impacts of fluid dynamics (e.g. flow velocity) in the male or the female genitalia, which have been shown to be highly sensitive with regard to structural variations, described for example in leaf beetles [72]. Nevertheless, the transfer of comparably large transfer forms in pholcids could serve in the context of sperm competition by occupying as much space as possible in the female sperm storage site and therefore reducing or blocking access for subsequent males, as has been suggested for Oonopidae [31]. However, the exact drivers of the evolution of sperm transfer forms remain dubious. For further understanding, more studies on female genitalia and the mating system in different pholcid taxa, especially the subfamily Ninetinae, are required.

#### Seminal secretions

Sperm in spiders are transferred with secretions produced in the testis and/or deferent ducts. These seminal secretions are structurally very diverse across spiders and may occupy various functional roles [40]. In pholcids, a variety of seminal secretions has been documented before [17, 20, 21]. Our data corroborate the structural diversity of seminal secretions in pholcids (Figs. 4, 5 and 6), suggesting that seminal secretions are under considerable selection and possibly involved in post-copulatory processes, as suggested previously for the wolf spider *Schizocosa malitiosa* (Tullgren, 1905) by Aisenberg and Costa [41]. In the ninetine species *Kambiwa neotropica* and *Galapa bella*, the tile-like seminal secretions resemble each other greatly, as do the synspermia (Figs. 5G, K; 18; 12). However, the synspermia of *Galapa* do not have a secretion sheath, contrary to *Kambiwa*. This suggests that the function of the secretions is more complex than just serving a purely protective role during sperm transfer or after copulation as described above for other cases of unsheathed sperm. In the theridiid spider *Tidarren argo* Knoflach & van Harten, 2001, a functional role of seminal secretions in terms of securing paternity has been discussed, as secretions are “cheaper” to produce than sperm and could therefore act as “cheap fillers” by simply occupying the maximum space within the female genital tract in order to impede insemination by subsequent males [43, 73]. An influence of various sperm-associated products within the ejaculate on fertilization success has been documented for other animal groups such as Leptidoptera [74–76] and *Drosophila* [77]. Further investigations, especially on the mating system and the fate of sperm after copulation, are needed to allow for more detailed insights into the functional role of seminal secretions in pholcids as well as in spiders in general.

#### Character evolution and phylogenetic implications

Our analysis clearly shows that many sperm characters within pholcid spiders are homoplastic, reflecting a rather dynamic evolution of sperm (Figs. 73 and 77). Character mapping revealed only few unambiguous synapomorphies as e. g. the presence of microtubules in the implantation fossa in Smeringopinae (Char. 20:1, see Fig. 77).

*Synspermia and cleistospermia.* Ninetinae are distinct from all remaining Pholcidae by the presence of synspermia as transfer forms (Fig. 75). The genus *Pholcophora,* as part of the “North and Central American/Caribbean clade” was suggested to be close to the Mexican genus *Tolteca* based on their geographic distributions [7]. Since in our study *Pholcophora* and *Tolteca* are the only ninetines with cleistospermia, our data suggest that the transformation from synspermia to cleistospermia took place once within Ninetinae, i.e. in the “North and Central American/Caribbean clade”. The genera *Guaranita* and *Kambiwa* both share similar spermatozoa and transfer form morphologies. Especially the synspermia with elongated nuclei as well as the peculiar secretions and structures surrounding the cluster of spermatozoa are very characteristic for both taxa. In contrast, the remaining Ninetinae taxa as e.g. *Nerudia* and *Gertschiola* show synspermia with stout nuclei that also lack the specific surrounding structures found in the synspermia of *Guaranita* and *Kambiwa.* A further Ninetinae genus represented in our taxon sampling is the genus *Galapa*. Huber, Eberle and Dimitrov [7] noted that the relationship of this genus with the remaining Ninetinae taxa was “entirely obscure”. Our analysis revealed very complex synspermia for this genus, which show an arrangement of spermatozoa that is very different compared to other Ninetinae. A single synsperm of *Galapa* comprises more than 60 individual spermatozoa, which are arranged in four clusters stacked on top of each other. The characteristics of the individual spermatozoa however resemble those of other Ninetinae.

**Fig. 76:**
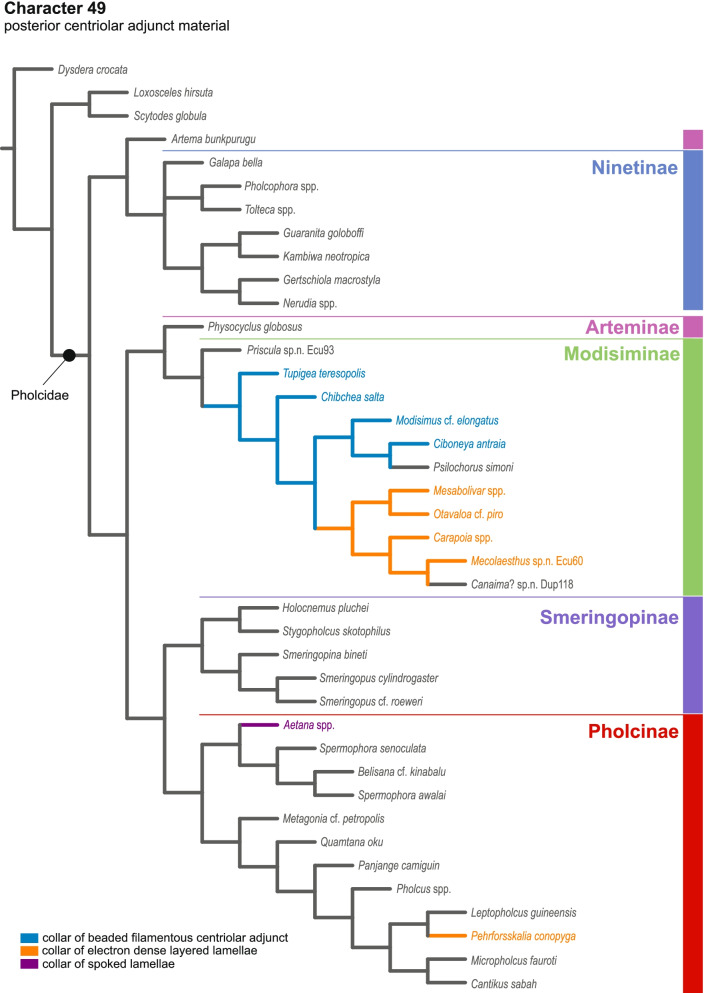
Scenario of the evolution of the organization of the posterior centriolar adjunct material (Character 49) across the pholcid tree of life. Character tracing using unambiguous optimization obtained with WinClada [50], phylogeny based on Huber et al*.* (2018) [7]

*Posterior centriolar adjunct.* Our data revealed that the posterior centriolar adjunct material (Char. 49) is a highly interesting character within Modisiminae (Fig. 76). The presence of a posterior centriolar adjunct shaped as a collar of electron dense material seems to be synapomorphic for most Modisiminae, where it is either shaped as layered lamellae (Char. 49:2) or beaded filaments (Char. 49:3). The former state appears to be a synapomorphy uniting the ‘*Mesabolivar* clade’ (all clade names sensu Huber, Eberle and Dimitrov [7]; herein *Mesabolivar* spp., *Otavaloa*), the ‘Venezuelan clade’ (herein *Mecolaesthus* sp.) and *Carapoia* (but not *Canaima,* whose phylogenetic position is dubious). The latter state (Char. 48:3) appears only in ‘’basal’ Modisiminae’ (sensu [7]) (herein *Tupigea teresopolis*, *Chibchea salta*, *Modisimus elongatus*, *Ciboneya antraia*), except for *Psilochorus simoni* and *Priscula*. Within the subfamily, the posterior centriolar adjunct material appears to have evolved from small beaded filaments (Char. 49:3) to longer and coherent lamellae (Char. 49:2). This assumption is supported by the difference in length of the individual lamellae, as in *Mecolaesthus* and *Carapoia* the lamellae of the centriolar adjunct material are conspicuously shorter than in the ‘*Mesabolivar* clade’, acting as a link between the beaded configuration and the extensive elongation of the lamellae. For the genus *Priscula*, our data revealed a notable similarity with *Psilochorus simoni* (data extracted from [21]), as does the investigated *Canaima* sp., with the reversion to an absence of posterior centriolar adjunct material being the most striking characteristic.

Outside of Modisiminae, a lamellated posterior centriolar adjunct material is only present in the pholcine *Pehrforsskalia conopyga.* This striking convergence could be explained with the hypothesized functions of the centriolar adjunct material (see above).

*Nucleus.* Nuclear characters are highly homoplastic, and of particular interest for Pholcinae as this subfamily shows the highest diversity in the pre- and postcentriolar part of the nucleus. For example, the postcentriolar elongation can be very long as in *Spermophora awalai* and *Aetana* spp. (representatives of the "group 1" sensu [7]) or rather short as in *Pholcus* and related genera ("group 3" sensu [7]). A character only present in Pholcinae is the helical band in the precentriolar part of the nucleus (Fig. 74). This structure was thought to be exclusive for *Pholcus* and its closest relatives (“group 3”), but occurs also in other Pholcinae like *Belisana* cf. *kinabalu* and *Spermophora senoculata* (“group 1”). Interestingly, a helical band is absent in the only representative of “group 2”, *Metagonia* cf. *petropolis*, and future studies should focus on putatively close relatives such as *Zatavua* and on further representatives of “group 1” to clarify the evolution of this structure within Pholcinae.

**Fig. 77:**
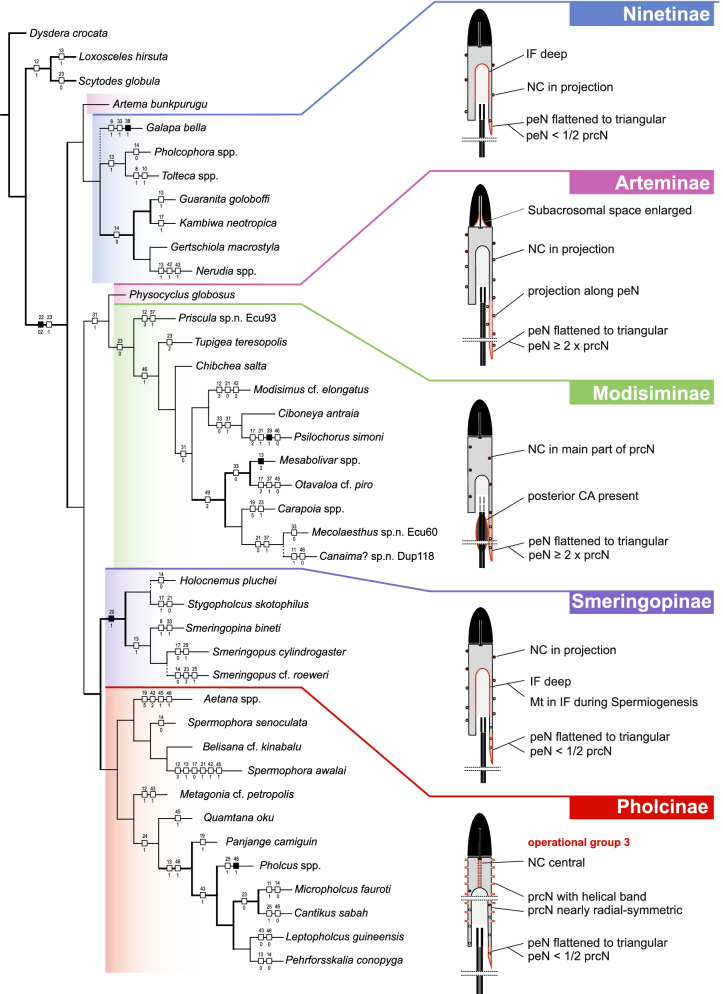
Sperm characters mapped on the phylogeny of Huber et al*.* (2018) [7]. Thick continuous lines: modest to full bootstrapping branch support, i.e. ≥ 70%; thin continuous lines: low bootstrapping branch support, i.e. < 70%; discontinuous lines: taxa not included in Huber et al*.* (2018) [7]—possible phylogenetic position based on comments in Huber et al*.* (2018) [7]. Number above squares: number of character; number below squares: character state. Full squares: characters without homoplasy; empty squares: homoplasic characters. On the right, characteristics of spermatozoa by subfamily are shown: The spermatozoa of each subfamily can be generally characterized by a combination of different character states highlighted in red in the schematic drawings

*Diversity on subfamily level.* According to Huber, Eberle and Dimitrov [7], the division of Pholcidae into five subfamilies is well supported by molecular data. Our observations reflect the distinctness of each subfamily as sperm characters allow—to a certain extent—for a definition of subfamily-related spermatozoa types (Fig. 77). This is especially interesting for Arteminae. Here, molecular data suggest a position of the genus *Artema* separate from ‘other Arteminae’ (herein represented by *Physocyclus globosus*) as sister to Ninetinae, which was however doubted with regard to somatic characters [7]. With regard to sperm morphology, the positioning of *Artema* separated from “other Arteminae” cannot be supported, since both taxa share most of spermatozoa and transfer form related characters (Fig. 77).

## Conclusions

Our study revealed a high morphological diversity in sperm morphology, which can be related to subfamily level to a certain extent as summarized in Fig. 77. The evolution of sperm characters appeared to be highly dynamic, with convergences across all subfamilies. Our analyses further provide a rich knowledge base for future studies on the morphology and evolution of the reproductive system of daddy long leg spiders. Moreover, we document several unique sperm traits not known in other spiders, such as the helical band in Pholcinae and the lamellate centriolar adjunct in Modisiminae. The functional roles of these structures remain unclear, but we suggest a correlation with sperm motility as a working hypothesis. Seminal secretions in Pholcidae are highly taxon-specific and may play a crucial role in pholcid reproduction. Future studies should focus on the biochemical composition of glandular products of the reproductive system as briefly explored by Uhl [78] for secretions of the female genitalia of *Pholcus phalangioides*. Finally, the interaction between the female reproductive tract and sperm needs to be explored in detail to understand the evolution of sperm (see also [42]). So far, for spiders, no data are available on the interaction of spermatozoa and female structures, but a co-evolution of sperm traits and female genitalia has recently been demonstrated in insects [35].

## Methods

### Taxon sampling

We studied 46 species from 33 genera, representing the five currently accepted subfamilies of Pholcidae (for details see Additional file 1). Depending on the availability of material, we studied between one to three specimen per species. As in other spider groups, we did not expect and could not observe an intraspecific variability of sperm characters, which also reflects what is described for other animal groups (e.g. [79]).

### Fixation and embedding

The primary male reproductive systems were dissected in the field or in the lab using 0.1 M phosphate buffer (PB) with 1.8% sucrose. Immediately after dissection, tissues were fixed overnight in 2.5% glutardialdehyde in PB (all taxa except for Ninetinae) or Karnovsky’s solution [80]; Ninetinae]. Afterwards, samples were washed with PB followed by a post fixation in buffered 2% osmium tetroxide solution for 2 h. Subsequently, samples were washed in PB and dehydrated using a graded series of ethanol. Embedding was carried out using the Embed812 resin embedding kit (Science Services GmbH, München, Germany). For the ninetine specimens, the samples were transferred into a “VacuTherm” vacuum heating cabinet (Thermo Fisher Scientific, Waltham, Massachusetts, USA) during the final pre-embedding step and incubated at 40 °C and 100 mbar for 3 × 30 min. Polymerization of the resin blocks was carried out in a heating cabinet at 60 °C for a minimum of 24 h.

### Histology

Semi-thin sections were used for the analysis of the general organization of the testes and deferent ducts. Embedded samples were sectioned using a Leica EM UC6 ultra-microtome (Leica Microsystems GmbH, Wetzlar, Germany), with a DiATOME histo Jumbo diamond knife (Diatome Ltd., Nidau, Switzerland) at a thickness of 700 nm. Staining was done with toluidine blue at 70 °C.

### Transmission electron microscopy

Ultra-thin sections as well as serial ultra-thin sections were obtained using a Leica EM UC6 ultra-microtome with a DiATOME ultra diamond knife at a section thickness of 70 nm. The sections were then transferred to formvar-coated copper slot grids (G2500C, Plano GmbH, Wetzlar, Germany), followed by staining with uranyl acetate and lead citrate for 4 min each. Sections were examined using a JEOL JEM-1011 Transmission Electron Microscope (JEOL Ltd., Akishima, Japan) with an Olympus Mega View III digital camera (Olympus K.K., Tokio, Japan) operated by an iTEM software package (iTEM Software, Whiteley, UK) as well as a Zeiss LEO 906 Transmission Electron Microscope (Carl Zeiss Microscopy GmbH, Jena, Germany).

### Micro-CT analysis of male genitalia

Male pedipalps were fixed with Karnovsky’s fixative or 80% ethanol, followed by dehydration using graded series of ethanol and staining in a 1% iodine solution in absolute ethanol overnight. Subsequently, the samples were re-transferred to absolute ethanol and dried using the automated critical point dryer Leica EM CPD300 (Leica Microsystems GmbH, Wetzlar, Germany). Samples were mounted and scanned using an Xradia MicroXCT-200 X-ray imaging system (Carl Zeiss Microscopy GmbH, Jena, Germany) at different magnifications and source voltages according to the specimen size.

### 3D Reconstruction of sperm transfer form and spermophor

3D reconstructions of sperm transfer forms are based on image stacks of serial ultra-thin sections (see above) from the deferent ducts. The image stacks were aligned using the Fiji plug-in TrackEM2 (following [81]) as well as Amira 6.4 (FEI Software, now Thermo Fisher Scientific, Waltham, Massachusetts, USA). The spermophor was reconstructed based on the microCT data. Surface reconstructions, data visualization and measurements were carried out in Amira 6.4 (for details see also [33]). Image stacks can be found in Morphobank (http://morphobank.org/permalink/?P4245).

### Graphical processing

If necessary, the contrast of images was enhanced using Corel PHOTO-PAINT 2017. The plates were composed with CorelDRAW 2017 (both Corel Corp., Ottawa, Ontario, Canada).

### Characters and reconstruction of their evolution

The data matrix includes a total of 40 terminals scored for 48 characters (see Additional file 2 and matrix stored in Morphobank project, see above). Genera with several species are each represented by one terminal, as follows: *Aetana* spp. 2 species, *Carapoia* spp. 2 species, *Mesabolivar* spp. 2 species, *Nerudia* spp. 2 species, *Pholcophora* spp. 2 species, *Pholcus* spp. 4 species, *Tolteca* spp. 2 species. Additionally, data for two further species of Pholcidae (*Psilochorus simoni, Holocnemus pluchei*) as well as for three outgroup species (*Loxosceles hirsuta* Mello-Leitão, 1931*, Scytodes globula* Nicolet, 1849*, Dysdera crocata* C. L. Koch, 1838) were extracted from Michalik and Ramírez [22].

Character conceptualization and terminology follows Michalik and Ramírez [22] (below abbreviated as MiRa) as well as Lipke and Michalik [31]. Character evolution was traced under parsimony using the software packages Mesquite 3.61 [82], Winclada [50] and TNT [83]. We used the phylogeny of Huber, Eberle and Dimitrov [7]. Investigated taxa not included in this phylogeny were placed following information given by Huber, Eberle and Dimitrov [7].

Based on our new data, we redefine two of the characters proposed by MiRa as follows (numbers correspond to MiRa):

- **Character 21:** Shape of postcentriolar elongation of the nucleus (in cross-section): 0 = round to oval; 1 = flattened to triangular; 2 = MiRa: “with a distinct projection” now scored in Character 45; 3 = flag-shaped (Fig. 7E in [31]).
- **Character 27:** Centriolar adjunct material redefined (see new characters 46–49). Original numbering of remaining characters stays unaffected.

Newly coded characters, continuing the numbering of MiRa [22] and Lipke and Michalik [31]:

- **Character 45: ***Projection along postcentriolar elongation of nucleus*: 0 = absent; 1 = present (as in Fig. 38D).
- **Character 46: ***Centriolar adjunct material*: 0 = absent; 1 = present.
- **Character 47: ***Centriolar adjunct material*: 0 = anterior (restricted to IF); 1 = posterior (centrioles towards posterior).
- **Character 48: ***Anterior centriolar adjunct:* 0 = electron dense homogenous (as in Fig. 61A); 1 = fibrillar (as in Fig. 65D).
- **Character 49: ***Posterior centriolar adjunct material:* 0 = electron dense chambered centriolar adjunct around anterior part of the axoneme (Fig. 6F in [22]); 1 = fibrillar chambered centriolar adjunct around anterior part of the axoneme; 2 = collar of electron dense layered (*new term*) lamellae around anterior part of the axoneme (Fig. 36); 3 = collar of beaded filamentous centriolar adjunct around anterior part of the axoneme (Figs. 31 and 32); 4 = collar of spoked lamellae around anterior part of axoneme (Fig. 50).

## Supplementary Information

**Additional file 1.** Voucher data.

**Additional file 2.** Morphological characters included in this study.

## Data Availability

The datasets generated and/or analyzed during the current study are available in the Morphobank repository, http://morphobank.org/permalink/?P4245.

## Abbreviations

AF: Acrosomal filament
AV: Acrosomal vacuole
Ax: Axoneme
C: Centriole
CA: Centriolar adjunct material
Chr: Condensed chromatin
Clei: Cleistospermium
Cys: Cyst of developing spermatids
dC: Distal centriole
DD: Deferent duct
ED: Ejaculatory duct
FT: Flagellar tunnel
GD: Golgi derivatives
Gly: Glycogen
IF: Implantation fossa
LuD: Lumen of deferent duct
LuT: Lumen of testis
Mi: Mitochondrion
MM: Manchette of microtubules
N: Nucleus
NC: Nuclear canal
pC: Proximal centriole
peN: Postcentriolar elongation of nucleus
prcN: Precentriolar part of nucleus
Sec: Secretions
Spt: Spermatid
SSh: Secretion sheath
STF: Sperm transfer form
Syn: Synsperm
VA: Vesicular area
Ww: “Water wheel” (sensu [16])

## Colour-coding in 3D-Models

Beige: Secretion sheath
Red: Acrosomal filament
Orange: Acrosomal vacuole
Dark green: Axoneme
Light green: Mitochondria
Magenta: Centriolar adjunct material
Dark blue: Precentriolar elongation of the nucleus
Light blue: Postcentriolar elongation of the nucleus

## References

[1] World Spider Catalog. Version 23.0. [http://wsc.nmbe.ch]

[2] Huber BA (2021). Beyond size: sexual dimorphisms in pholcid spiders. Arachnology.

[3] Eberle J, Dimitrov D, Valdez-Mondragon A, Huber BA. Microhabitat change drives diversification in pholcid spiders. BMC Evol Biol. 2018; 18. 10.1186/s12862-018-1244-8 PMC6145181 30231864

[4] Dimitrov D, Astrin JJ, Huber BA (2013). Pholcid spider molecular systematics revisited, with new insights into the biogeography and the evolution of the group. Cladistics.

[5] Huber BA (2000). New world pholcid spiders (Araneae : Pholcidae): A revision at generic level. B Am Mus Nat Hist.

[6] Huber BA (2011). Phylogeny and classification of Pholcidae (Araneae): an update. J Arachnol.

[7] Huber BA, Eberle J, Dimitrov D (2018). The phylogeny of pholcid spiders: a critical evaluation of relationships suggested by molecular data (Araneae, Pholcidae). Zookeys.

[8] Cargnelutti F, Calbacho-Rosa L, Córdoba-Aguilar A, Peretti AV (2022). Successive matings affect copulatory courtship but not sperm transfer in a spider model. Biol J Linn Soc.

[9] Cargnelutti F, Calbacho-Rosa L, Peretti AV (2021). Genital movements are not restricted to spermatozoa transfer in a haplogyne spider. Ethology.

[10] Huber BA, Nuneza OM (2015). Evolution of genital asymmetry, exaggerated eye stalks, and extreme palpal elongation in *Panjange* spiders (Araneae: Pholcidae). Eur J Taxon.

[11] Huber BA (2004). Evidence for functional segregation in the directionally asymmetric male genitalia of the spider *Metagonia mariguitarensis* (Gonzalez-Sponga) (Pholcidae: Araneae). J Zool.

[12] Huber BA (2006). Cryptic female exaggeration: The asymmetric female internal genitalia of *Kaliana yuruani* (Araneae: Pholcidae). J Morphol.

[13] Huber BA, González AP (2001). Female genital dimorphism in a spider (Araneae: Pholcidae). J Zool.

[14] Uhl G (1994). Genital morphology and sperm storage in *Pholcus phalangioides* (Fuesslin, 1775) (Pholcidae; Araneae). Acta Zoologica.

[15] Uhl G (1998). Mating behaviour in the cellar spider, *Pholcus phalangioides,* indicates sperm mixing. Anim Behav.

[16] Alberti G, Weinmann C (1985). Fine structure of spermatozoa of some labidognath spiders (Filistatidae, Segestriidae, Dysderidae, Oonopidae, Scytodidae, Pholcidae; Araneae; Arachnida) with remarks on spermiogenesis. J Morphol.

[17] Michalik P, Uhl G (2005). The male genital system of the cellar spider *Pholcus phalangioides* (Fuesslin, 1775) (Pholcidae, Araneae): development of spermatozoa and seminal secretion. Front Zool.

[18] Rosati F, Baccetti B, Dallai R. The spermatozoon of Arthropoda. X. Araneids and the lowest Myriapods. Comparative Spermatology. 1970:247–254.

[19] Lopez A, Boissin L. Spermatide d'Holocnemus pluchei (Scop.)(Arachnida, Araneida, Pholcidae): étude ultrastructurale. Bull Soc Zool Fr. 1976; 101:423–431.

[20] Michalik P, Dallai R, Giusti F, Mercati D, Alberti G (2005). Spermatozoa and spermiogenesis of *Holocnemus pluchei* (Scopoli, 1763) (Pholcidae, Araneae). Tissue Cell.

[21] Michalik P, Huber BA (2006). Spermiogenesis in *Psilochorus simoni* (Berland, 1911) (Pholcidae, Araneae): evidence for considerable within-family variation in sperm structure and development. Zoology.

[22] Michalik P, Ramírez MJ (2014). Evolutionary morphology of the male reproductive system, spermatozoa and seminal fluid of spiders (Araneae, Arachnida) – Current knowledge and future directions. Arthropod Struct Dev.

[23] Fernández R, Kallal RJ, Dimitrov D, Ballesteros JA, Arnedo MA, Giribet G, Hormiga G (2018). Phylogenomics, diversification dynamics, and comparative transcriptomics across the spider tree of life. Curr Biol.

[24] Kallal RJ, Kulkarni SS, Dimitrov D, Benavides LR, Arnedo MA, Giribet G, Hormiga G (2021). Converging on the orb: denser taxon sampling elucidates spider phylogeny and new analytical methods support repeated evolution of the orb web. Cladistics.

[25] Ramírez MJ, Magalhaes ILF, Derkarabetian S, Ledford J, Griswold CE, Wood HM, Hedin M (2021). Sequence Capture Phylogenomics of True Spiders Reveals Convergent Evolution of Respiratory Systems. Syst Biol.

[26] Foelix R. Biology of Spiders, 3rd ed: Oxford University Press; 2011.

[27] Alberti G. Chelicerata. Reproductive biology of invertebrates. 2000; 9(Part B):311–388.

[28] Alberti G (1990). Comparative spermatology of Araneae. Acta Zoologica Fennica.

[29] Simon E (1893). Histoire Naturelle des Araignées.

[30] Alberti G, Coyle FA. Ultrastructure of the primary male genital system, spermatozoa, and spermiogenesis of Hypochilus pococki (Araneae, Hypochilidae). J Arachnol. 1991:136–149.

[31] Lipke E, Michalik P (2015). Evolutionary morphology of the primary male reproductive system and spermatozoa of goblin spiders (Oonopidae; Araneae). Bull Am Mus Nat Hist.

[32] Michalik P, Haupt J, Alberti G (2004). On the occurrence of coenospermia in mesothelid spiders (Araneae: Heptathelidae). Arthropod Struct Dev.

[33] Lipke E, Ramírez MJ, Michalik P (2014). Ultrastructure of spermatozoa of Orsolobidae (Haplogynae, Araneae) with implications on the evolution of sperm transfer forms in Dysderoidea. J Morphol.

[34] García-González F, Simmons LW (2007). Shorter sperm confer higher competitive fertilization success. Evolution.

[35] Simmons LW, Garcia-Gonzalez F (2021). Can Sexual Selection Drive the Evolution of Sperm Cell Structure?. Cells.

[36] den Boer SP, Baer B, Boomsma JJ (2010). Seminal fluid mediates ejaculate competition in social insects. Science.

[37] Den Boer SPA, Boomsma JJ, Baer B (2008). Seminal fluid enhances sperm viability in the leafcutter ant *Atta colombica*. Behav Ecol Sociobiol.

[38] Pitnick S, Wolfner M, Suarez S, Birkhead T, Hosken D, Pitnick S (2009). Ejaculate-female and sperm-female interactions. Sperm biology- an evolutionary perspective.

[39] Wigby S, Sirot LK, Linklater JR, Buehner N, Calboli FCF, Bretman A, Wolfner MF, Chapman T (2009). Seminal Fluid Protein Allocation and Male Reproductive Success. Curr Biol.

[40] Michalik P (2009). The male genital system of spiders (Arachnida, Araneae) with notes on the fine structure of seminal secretions. Contrib Nat Hist.

[41] Aisenberg A, Costa FG (2005). Females Mated without Sperm Transfer Maintain High Sexual Receptivity in the Wolf Spider *Schizocosa malitiosa*. Ethology.

[42] Herberstein ME, Schneider JM, Uhl G, Michalik P (2011). Sperm dynamics in spiders. Behav Ecol.

[43] Michalik P, Knoflach B, Thaler K, Alberti G (2005). The spermatozoa of the one-palped spider *Tidarren argo* (Araneae, Theridiidae). J Arachnol.

[44] Costa-Ayub CL, Faraco CD (2007). Ultrastructural aspects of spermiogenesis and synspermia in the brown spider *Loxosceles intermedia* (Araneae: Sicariidae). Arthropod Struct Dev.

[45] Michalik P, Dallai R, Giusti F, Alberti G (2004). The ultrastructure of the peculiar synspermia of some Dysderidae (Araneae, Arachnida). Tissue Cell.

[46] Griffin FJ, Shigekawa K, Clark WH (1988). Formation and structure of the acrosomal filament in the sperm of *Sicyonia ingentis*. J Exp Zool.

[47] Psenicka M, Rodina M, Linhart O (2010). Ultrastructural study on the fertilisation process in sturgeon (*Acipenser*), function of acrosome and prevention of polyspermy. Anim Reprod Sci.

[48] Lipke E, Michalik P (2012). Formation of primary sperm conjugates in a haplogyne spider (Caponiidae, Araneae) with remarks on the evolution of sperm conjugation in spiders. Arthropod Struct Dev.

[49] Michalik  P (2007). Spermatozoa and spermiogenesis of Liphistius cf. phuketensis (Mesothelae, Araneae, Arachnida) with notes on phylogenetic implications. Arthropod Struct Dev.

[50] Nixon K. WinClada, version 1.00. 08. In*.* Ithaca, NY: Published by the author; 2002.

[51] Michalik P, Sacher P, Alberti G (2006). Ultrastructural observations of spermatozoa of several tetragnathid spiders with phylogenetic implications (Araneae, Tetragnathidae). J Morphol.

[52] Higginson DM, Miller KB, Segraves KA, Pitnick S (2012). Female reproductive tract form drives the evolution of complex sperm morphology. P Natl Acad Sci USA.

[53] Fawcett DW, Phillips DM (1969). The fine structure and development of the neck region of the mammalian spermatozoon. Anat Rec.

[54] Avidor-Reiss T, Mazur M, Fishman EL, Sindhwani P (2019). The role of sperm centrioles in human reproduction–the known and the unknown. Front Cell Dev Biol.

[55] Aire TA, Ozegbe P (2012). Components and development of the centriolar complex during and beyond spermiogenesis in a passeridan bird, the Masked weaver (*Ploceus velatus*). Tissue Cell.

[56] Oakley BR (1999). γ-Tubulin. Curr Top Dev Biol.

[57] Wilson P, Zheng Y, Oakley C, Oakley B, Borisy G, Fuller M (1997). Differential expression of two γ-tubulin isoforms during gametogenesis and development in *Drosophila*. Dev Biol.

[58] Dallai R, Paoli F, Mercati D, Lupetti P (2016). The centriole adjunct of insects: need to update the definition. Tissue Cell.

[59] Fouquet J, Kann M, Soues S, Melki R (2000). ARP1 in Golgi organisation and attachment of manchette microtubules to the nucleus during mammalian spermatogenesis. J Cell Sci.

[60] Michalik P, Aisenberg A, Postiglioni R, Lipke E (2013). Spermatozoa and spermiogenesis of the wolf spider *Schizocosa malitiosa* (Lycosidae, Araneae) and its functional and phylogenetic implications. Zoomorphology.

[61] Higginson DM, Pitnick S (2011). Evolution of intra-ejaculate sperm interactions: do sperm cooperate?. Biol Rev.

[62] Liebrich W, Hanna PJ, Hess O (1982). Evidence for asynchronous mitotic cell divisions in secondary spermatogonia of *Drosophila*. Int J Invertebr Reprod.

[63] Schärer L, Da Lage J-L, Joly D (2008). Evolution of testicular architecture in the Drosophilidae: A role for sperm length. BMC Evol Biol.

[64] Vöcking O, Uhl G, Michalik P (2013). Sperm dynamics in spiders (Araneae): ultrastructural analysis of the sperm activation process in the Garden Spider *Argiope bruennichi* (Scopoli, 1772). PLoS ONE.

[65] Tuni C, Schneider J, Uhl G, Herberstein ME (1813). Sperm competition when transfer is dangerous. Philos Trans R Soc B.

[66] Talarico G, Hernandez LG, Michalik P (2008). The male genital system of the New World Ricinulei (Arachnida): ultrastructure of spermatozoa and spermiogenesis with special emphasis on its phylogenetic implications. Arthropod Struct Dev.

[67] Alberti G, Coons L, Harrison F, Foelix R (1999). Acari: mites. Chelicerata, Arthropoda.

[68] Lopez A, Boissin L (1975). Observation de spermatozoides non enkystés chez une araignée du genre *Phoroncidia* (Araneae, Theridiidae). Bull Soc Zool Fr.

[69] Uhl G. Mating behaviour and female sperm storage in *Pholcus phalangioides* (Fuesslin) (Araneae). Mem Queensl Mus. 1993; 33(2).

[70] Kuntner M, Coddington JA, Schneider JM (2009). Intersexual arms race? Genital coevolution in nephilid spiders (Araneae, Nephilidae). Evolution.

[71] Presgraves DC, Baker RH, Wilkinson GS (1999). Coevolution of sperm and female reproductive tract morphology in stalk-eyed flies. Proc Royal Soc B.

[72] Matsumura Y, Gürke S, Tramsen HT, Gorb SN (2020). 3D printed spermathecae as experimental models to understand sperm dynamics in leaf beetles. BMC Zoology.

[73] Michalik P, Knoflach B, Thaler K, Alberti G (2010). Live for the moment - Adaptations in the male genital system of a sexually cannibalistic spider (Theridiidae, Araneae). Tissue Cell.

[74] Cook PA, Wedell N (1996). Ejaculate dynamics in butterflies: a strategy for maximizing fertilization success?. Proc R Soc B: Biol Sci.

[75] Swallow JG, Wilkinson GS (2002). The long and short of sperm polymorphisms in insects. Biol Rev Camb Philos Soc.

[76] Wedell N (2005). Female receptivity in butterflies and moths. J Exp Biol.

[77] Holman L, Snook RR (2008). A sterile sperm caste protects brother fertile sperm from female-mediated death in *Drosophila pseudoobscura*. Curr Biol.

[78] Uhl G (1996). Sperm storage secretion of female cellar spiders (*Pholcus phalangioides*; Araneae): a gel-electrophoretic analysis. J Zool.

[79] Rheubert J, Messak JA, Siegel DS, Gribbins KM, Trauth SE, Sever DM (2017). Inter- and intraspecific variation in sperm morphology of *Sceloporus consobrinus* and *Sceloporus undulatus* (Squamata: Phrynosomatidae). Biol J Linn Soc.

[80] Karnovsky MJ (1965). A Formaldehyde-Glutaraldehyde Fixative of High Osmolality for Use in Electron Microscopy. J Cell Biol.

[81] Saalfeld S, Fetter R, Cardona A, Tomancak P (2012). Elastic volume reconstruction from series of ultra-thin microscopy sections. Nat Methods.

[82] Maddison W, Maddison D. Mesquite: A modular system for evolutionary analysis. Version 3.61. 2019. In*.*; 2019.

[83] Goloboff PA, Catalano SA (2016). NT version 1.5, including a full implementation of phylogenetic morphometrics. Cladistics.

