# Supplementary material for: Evolutionary morphology of sperm in pholcid spiders (Pholcidae, Synspermiata)
Source: BMC Zool. 2022 Sep 26;7:52. doi: 10.1186/s40850-022-00148-3 (PMC10127419; doi:10.1186/s40850-022-00148-3)
Supplement: Supplementary file 1 — Additional file 1. Voucher data. [file 40850_2022_148_MOESM1_ESM.pdf]

Appendix 1. Collecting data of the studied taxa and depository information of voucher specimens (ZFMK, Zoologisches Forschungsmuseum Alexander Koenig, Bonn, MACN, Museo Argentino Ciencias Naturales, Buenos Aires, Argentina, ZIMG, Zoologisches Institut und Museum, Greifswald)

Ninetinae

Galapa bella (Gertsch & Peck, 1992) (PM-0347) - GALÁPAGOS ISLANDS: Santa Cruz, 1 km SW Garrapatero beach (0.7007°S, 90.2280°W), 22 Aug. 2019, leg./det. B.A. Huber; ZFMK

Gertschiola macrostyla (Mello-Leitão, 1941) (PM-0002) – ARGENTINA: San Juan, Valle Fértil, Chucuma, 22.01.2012, leg. J.M.A. Navarro, det. C. Grismado; ZFMK

Guaranita goloboffi Huber, 2000 (PM-0326) – ARGENTINA: Salta, Cabra Corral, 'site 3', ~3.5 km SE of dam (25.2907°S, 65.3057°W), 21.iii.2019, leg./det. B.A. Huber; ZFMK

Guaranita goloboffi Huber, 2000 (PM-0338, PM-0339) – ARGENTINA: Catamarca, ~5 km NW Chumbicha (28.8152°S, 66.2478°W), 28-29.iii.2019, leg./det. B.A. Huber; ZFMK

Kambiwa neotropica (Kraus, 1957) (PM-0250, PM-0251) – Brazil, Rio Grande do Norte, near Felipe Guerra, Lajedo do Arapuá (5°31.75'S, 37°36.86'W), 8.vi.2015, leg./det. B.A. Huber; ZFMK

***Nerudia* sp. n. 'Arg58'** (PM-0320) – ARGENTINA: Salta, ~15 km NW Campo Quijano (24.7918°S, 65.7297°W), 19.iii.2019, leg./det. B.A. Huber; ZFMK

***Nerudia* sp. n. 'Mic20'**(PM-0334) – ARGENTINA: Catamarca, ~10 km N Belén (27.5641°S, 67.0058°W), 25.iii.2019, leg./det. B.A. Huber; ZFMK

***Pholcophora* sp. n. 'Mex22'** (PM-0702) **-** MEXICO: Guerrero, ~5 km S Papanoa (17.2711°N, 101.0328°W), 4.x.2019, leg./det. B.A. Huber; ZFMK

***Pholcophora* sp. n. 'Mex157'** (PM-0706) - MEXICO: Puebla, ~35 km SE Tehuacan, N of Calapa bridge (18.1652°N, 97.2605°W), 24.x.2019, leg./det. B.A. Huber; ZFMK

Tolteca hesperia (Gertsch, 1982) (PM-0704, PM-0705) - MEXICO: Sinaloa, ~3 km S Rosario (22.9584°N, 105.8490°W), 9.x.2019, leg./det. B.A. Huber; ZFMK

***Tolteca* sp. n. ‘Mex169’** (PM-0707, PM-0709) - MEXICO: Oaxaca, ~3 km N San Pedro Totolapa (16.6976°N, 96.3180°W), 26.x.2019, leg./det. B.A. Huber; ZFMK

***Tolteca* sp. n. ‘Mex169’** (PM-0711) - MEXICO: Oaxaca, ~17 km NW Tehuantepec (16.3919°N, 95.3865°W), 27.x.2019, leg./det. B.A. Huber; ZFMK

Arteminae

Artema bunkpurugu Huber & Kwapong, 2013 (PM-0739) - GUINEA: Basse-Guinée, Kindia (10°03’N, 12°51’W), 5.xii.2008, leg./det. B.A. Huber; ZFMK

Physocyclus globosus (PM-0118) - CUBA: Pinar del Río, Viñales (22.622°N, 83.737°W), 22.iv.2012, leg./det. B.A. Huber; ZFMK

Modisiminae

***Canaima*? sp. n. ‘Dup118’** (PM-EC-084) – ECUADOR: Prov. St. Domingo, Otonga forest, 07.12.2009, leg. P. Michalik, det. B.A. Huber; ZFMK

Carapoia lutea (PM-0735) – ARGENTINA: Misiones, PN Iguazú, Area Garganta del Diablo, 16-20 May 2005, leg. M. Ramírez, P. Michalik, det. B. A. Huber, MACN

Carapoia nairae Huber, 2016 (PM-0740) - BRAZIL: Espirito Santo, Vargem Alta, Fazenda Monte Verde (20°27.6-28.2’S, 40°59.5’-41°00.2’W), 2-3.x.2010, leg./det. B.A. Huber; ZFMK

Chibchea salta Huber, 2000 (PM-0754) - ARGENTINA: Jujuy province, P. Nacional Calilegua, Monolito, 23° 40' 52" S, 64° 54' 05" W, 06-11.XII.2008, leg. C. Grismado, M. Izquierdo, F. Labarque, G. Rubio, M. Burger, P. Michalik, P. Carrera, A. Ojanguren, C. Mattoni, det. C. Grismado, MACN

Ciboneya antraia Huber & Pérez, 2001 (PM-0121) - CUBA: Pinar del Río: Viñales, Cueva del Cable (22.667°N, 83.707°W), 20.iv.2012, leg./det. B.A. Huber; ZFMK

***Mecolaesthus* sp. n. ‘Ecu60’** (PM-EC-036) – ECUADOR: Napo, 10 km Road Archidona-Rio Hollin, 29.11.2009, leg. P. Michalik, det. B.A. Huber; ZFMK

Mesabolivar iguazu Huber, 2000 (PM-0755) – ARGENTINA: Misiones, PN Iguazú, Macuco trail, 16-20 May 2005, leg. M. Ramírez, P. Michalik, det. C. Grismado, MACN

Mesabolivar cyaneotaeniatus (Keyserling, 1891) (PM-0742) – BRAZIL: Rio de Janeiro Prov., Cachoeira da Pedra Branca near Parati (23°11,8’S, 44°46,0’W), 22.viii.2007, leg./det. B.A. Huber; ZFMK

Modisimus elongatus Bryant, 1940 (PM-0119) – CUBA: Pinar del Río, Viñales, forest at base of mogote, ‘site 1’ (22.622°N, 83.737°W), 22.iv.2012, leg./det. B.A. Huber; ZFMK

Otavaloa* cf. *piro (PM-EC-067) – ECUADOR: Yasuni, Botanica trail, 02.12.2009, leg. P. Michalik, det. B.A. Huber; ZFMK

***Priscula* sp. n. ‘Ecu93’** (PM-EC-040) – ECUADOR: Napo, 10 km Road Archidona-Rio Hollin, 29.11.2009, leg. P. Michalik, det. B.A. Huber; ZFMK

Tupigea teresopolis Huber, 2000 (PM-0750) – BRAZIL: Rio de Janeiro: 4 km NW Penedo, 22◦24.5'S, 44◦33.0' to 33.4'W, 14–16 Aug. 2007, leg./det. B.A. Huber; ZFMK

Smeringopinae

Smeringopina bineti (Millot, 1941) (PM-0753) – GUINEA: Basse-Guinée, near Kindia (10°00.8’N, 12°48.6’W), 5.xii.2008, leg./det. B.A. Huber; ZFMK

Smeringopus* cf. *roeweri (PM-0751) – TANZANIA: Singida province, Mahenge, close to the village of Mwaru, 4° 47' 50.2" S, 34° 15' 54.5"E, July 2002, leg. P. Michalik, det. B.A. Huber; ZFMK

Smeringopus cylindrogaster (Simon, 1907) (PM-0749) – CAMEROON: Littoral Region, near Loum (4°43.6’N, 9°42.5’E), 24.iv.2009 leg./det. B. Huber; ZFMK

Stygopholcus skotophilus Kratochvíl, 1914 (PM-0226) – BOSNIA AND HERZEGOVINA: Republika Srpska, Pavlova pećina (St. Paul’s cave) (42.6665°N, 18.3078°E), 26.05.2014, leg./det. B.A. Huber; ZFMK

Pholcinae

Aetana poring Huber, 2015 (PM-0227) – MALAYSIA-BORNEO: *Sabah*: Mt. Kinabalu, forest along Silau Silau Trail, 6.010-6.017°N, 116.537-116.543°E, 6.viii.2014, leg./det. B.A. Huber; ZFMK

Aetana loboc Huber, 2015 (PM-0222) – PHILIPPINES: Bohol Island, near Loboc, above Loboc River (9.651°N, 124.022°E), 05.03.2014, leg./det. B.A. Huber; ZFMK

Belisana* cf. *kinabalu (PM-0230, PM-0231) – MALAYSIA-BORNEO: Sabah, Sepilok, Rainforest Discovery Centre, forest along Pitta Trail, 5.875-5.878°N, 117.937-117.942°E, 9.viii.2014, leg./det. B.A. Huber; ZFMK

Cantikus sabah (Huber, 2011) (PM-0228) – MALAYSIA-BORNEO: Sabah, Sepilok, Rainforest Discovery Centre, forest along Pitta Trail (5.875-5.878°N, 117.937-117.942°E), 9.viii.2014, Leg./det. B.A. Huber; ZFMK

Leptopholcus guineensis Millot, 1941 (PM-0741) – GUINEA: Basse-Guinée, near Kindia (10°00.8'N, 12°48.6'W), 5.xii.2008, leg./det. B.A. Huber; ZFMK

Metagonia* cf. *petropolis (PM-0743) – BRAZIL: Rio de Janeiro, 4 km NW Penedo, 22◦24.5'S, 44◦33.0' to 33.4'W, 14–16 Aug. 2007, leg./det. B.A. Huber; ZFMK

Micropholcus fauroti (Simon, 1887) (PM-0249) – BRAZIL: Rio Grande do Norte, Apodi (5.66°S, 37.80°W), 8.vi.2015, leg./det. B.A. Huber; ZFMK

Panjange camiguin Huber, 2015 (PM-0220) – PHILIPPINES: Bohol Island, near Loboc, above Loboc River (~9.655°N, 124.015°E), 05.03.2014, leg./det. B.A. Huber; ZFMK

Pehrforsskalia conopyga Deeleman-Reinhold & van Harten, 2001 (PM-0745) – CAMEROON: Northwest Region, near Oku (6°14.2'N, 10°31.5'E), 17.iv.2009, leg./det. B.A.Huber; ZFMK

Pholcus attuleh Huber, 2011 (PM-0738) – CAMEROON: Southwest Region, near Dschang, Attuleh, 'site 1' (5°27.7'N, 9°56.5'E), 20.iv.2009, leg./det. B.A. Huber; ZFMK

Pholcus bamboutos Huber, 2011 (PM-0746) – CAMEROON: West Region, near Mbouda, Bamboutos (5°37.3'N, 10°06.7'E), 19.iv.2009, leg./det. B. Huber; ZFMK

Pholcus kindia Huber, 2011 (PM-0737) – GUINEA: Basse-Guinée, near Kindia (10°00.8'N, 12°48.6'W), 5.xii.2008, leg./det. B.A. Huber; ZFMK

Pholcus opilionides (Schrank, 1781) (PM-0736) – GERMANY: Mecklenburg-Vorpommern, Kirchdorf, 14.09.2005, leg./det. G.Alberti, ZIMG

Quamtana oku Huber, 2003 (PM-0747) – CAMEROON: Northwest Region, near Oku (6°14.2’N, 10°31.5’E), 17.iv.2009, leg./det. B.A. Huber; ZFMK

Spermophora awalai Huber, 2014 (PM-0748) – CAMEROON: Southwest Region, near Dschang, Attuleh, site 1 (5°27.7’N, 9°56.5’E), 20.iv.2009, leg./det. B.A. Huber; ZFMK

Spermophora senoculata (Dugès, 1836) (PM-0734) – SPAIN: L'Estartit, July 1984, leg./det. G. Alberti; ZFMK
