# Supplementary material for: Evolutionary morphology of sperm in pholcid spiders (Pholcidae, Synspermiata)
Source: BMC Zool. 2022 Sep 26;7:52. doi: 10.1186/s40850-022-00148-3 (PMC10127419; doi:10.1186/s40850-022-00148-3)
Supplement: Supplementary file 2 — Additional file 2. Morphological characters included in this study. [file 40850_2022_148_MOESM2_ESM.docx]

**Appendix 2.** Morphological characters included in this study. Characters are adapted from Michalik and Ramírez [22] (abbreviated as “MiRa”) and Lipke and Michalik [31]. Note, that these characters are intended to be applicable for sperm morphology across all spider families, hence certain characters included are invariant or inapplicable for Pholcidae.

**Character 1**

Testes: 0 = paired; 1 = distally fused; 2 = proximally fused; 3 = completely fused.

*Length: 2, CI: 0.5, RI: 0*

All pholcid species for which data regarding the gross morphology of the reproductive system are available have paired testes.

**Character 2**

Shape of the testes (if not completely fused): 0 = tubular; 1 = oval; 2 = spherical.

*Autapomorphic.*

All pholcid species studied with respect to this character have tubular testes except for the modisimine species *Psilochorus simoni*, which has oval testes (Michalik and Huber []21]).

**Character 3**

Testes diameter: 0 = >2x of deferent duct; 1 = ±equal to deferent duct.

*Uninformative.*

**Character 4:** Deferent ducts: 0 = long (>1.5 length of testis); 1 = short (< length of testis).

Note that character state 0 comprises extensive morphological variation as the deferent duct can be more than ten times the length of the testis.

*Length: 4, CI: 0.25, RI: 0*

Most studied species have short deferent ducts. Long deferent ducts occur in *Galapa* *bella* (Ninetinae), *Physocyclus* *globosus* (Arteminae), and *Panjange* *camiguin* (Pholcinae). The plesiomorphic condition in Pholcidae is unclear.

**Character 5:** Seminal vesicle: 0 = absent; 1 = present.

*Uninformative.*

**Character 6:** Spermatogenesis: 0 = continuous throughout adulthood; 1 = permanently terminated in late subadult or early adult stage indicated by the absence of generative tissue within the testes.

*Uninformative.*

**Character 7:** Spermatozoa in adult deferent ducts: 0 = present; 1 = depleted.

*Uninformative.*

**Character 8:** General appearance of the acrosomal vacuole: 0 = cylindrical (nearly same diameter over the whole length); 1 = conical (widens toward the posterior); 2 = screw-shaped; 3 = twisted.

*Length: 7, CI: 0.14, RI: 0.14*

Most of the investigated species share a cylindrical acrosomal vacuole, which is apparently plesiomorphic for Pholcidae. A conial vacuole seems to have evolved several times independently in ninetines, smeringopines, and pholcines.

**Character 9:** Shape of the acrosomal vacuole near the anterior pole of the nucleus: 0 = straight; 1 = constricted.

*Uninformative.*

**Character 10:** Posterior part of the acrosomal vacuole: 0 = planar; 1 = extending into the anterior indentation of the nucleus.

*Length: 13, CI: 0.08, RI: 0.08*

Most of the investigated species have an acrosomal vacuole that is planar at its posterior part. However, in several taxa within all five pholcid subfamilies, a configuration of an acrosomal vacuole extending into an anterior indentation of the nucleus is present. The plesiomorphic condition for Pholcidae is unclear.

**Character 11:** Subacrosomal space: 0 = narrow (similar to the diameter of the AF); 1 = enlarged toward posterior (several times the diameter of the AF).

*Length: 6, CI: 0.17, RI: 0.17*

The subacrosomal space in most investigated pholcids appeared to be narrow. It is enlarged towards posterior in *Artema* *bunkpurugu*, *Physocyclus globosus*, *Priscula* sp. n. Ecu93, *Canaima* sp. n. Dup118 and *Micropholcus fauroti*. The plesiomorphic condition for Pholcidae is unclear.

**Character 12:** Length of acrosomal filament: 0 = until end of nuclear canal; 1 = ends clearly after the axonemal basis; 2 = ends clearly before axonemal basis; 3 = ends in the region of the axonemal basis.

*Length: 9, CI: 0.22, RI: 0.13*

An acrosomal filament that ends clearly before the axonemal basis is common in and probably plesiomorphic for Pholcidae. In some Modisiminae and in the pholcine *Spermophora* *awalai* it ends in the region of the axonemal basis, while it ends after the axonemal basis in other Modisiminae and in the pholcine *Metagonia* cf. *petropolis*.

**Character 13:** Chromatin condensation pattern in early to mid spermatids: 0 = fibrillar; 1 = fibrillar with electron-dense streaks; 2 = globular (only in early spermatids); 3 = granular.

*Length: 11, CI: 0.18, RI: 0.36*

In many of the studied species, the condensation pattern of chromatin in early to mid spermatids is fibrillary, which is apparently the plesiomorphic condition in Pholcidae. Exceptions are a globular pattern in *Mesabolivar* spp. and a fibrillary pattern with electron dense streaks in several representatives of Ninetinae, Modisiminae, Smeringopinae, and Pholcinae. A granular condensation pattern does not occur in pholcids.

**Character 14:** Anterior pole of the nucleus: 0 = planar; 1 = indented, bowl-shaped; 2 = deeply indented, extends in the periphery throughout the anterior third of the nucleus. The indentation is defined by the presence of the electron-dense plate on its internal border.

*Length: 13, CI: 0.08, RI: 0.33*

This character is evolutionary highly unstable. Most subfamilies include species with a planar anterior pole of the nucleus and species with an indented shape of the anterior pole of the nucleus. Character state 2 was never observed in Pholcidae.

**Character 15:** Anterior third of the precentriolar region of the nucleus (prcN): 0 = straight; 1 = twisted.

*Uninformative*.

**Character 16:** Plate-like extension of the nucleus on one side of the acrosomal vacuole: 0 = absent; 1 = present.

*Length: 2, CI: 0.5, RI: 0*

Only the the pholcines *Aetana* spp. and *Spermophora* *senoculata* shared a plate-like extension of the nucleus.

**Character 17:** Depth of the implantation fossa: 0 = small (ca. <1/3 of prcN length); 1 = medium (about half of prcN length); 2 = large (ca. >2/3 prcN length).

*Length: 15, CI: 0.13, RI: 0.32*

The depth of the implantation fossa varies strongly among the investigated species. All three character states occur in pholcids. Notably, a shallow implantation fossa may be synapomorphic for a group of modisimines such as *Modisimus* *elongatus*, *Ciboneya antraia*, *Mesabolivar* spp., *Carapoia* spp., *Mecolaesthus* sp. n. Ecu69 and *Canaima*? sp. n. Dup118; however, a shallow implantation fossa is also shared by some representatives of Smeringopinae and Pholcinae. The plesiomorphic condition for Pholcidae is unclear.

**Character 18:** Location of the implantation fossa: 0 = prcN; 1 = peN.

*Uninformative*.

**Character 19:** Type of content of the implantation fossa: 0 = glycogen; 1 = distinct centriolar adjunct; 2 = globular secretion; 3 = mitochondria; 4 = anterior part of the axoneme; 5 = only centrioles.

*Length: 8, CI: 0.25, RI: 0*

Glycogen is the most common content of the implantation fossa in the studied species and probably plesiomorphic for Pholcidae; less common are a distinct anterior centriolar adjunct (*Panjange* *camiguin*, *Micropholcus fauroti*) and only centrioles (*Carapoia* spp., *Aetana* spp.).

**Character 20:** Microtubules inside implantation fossa during spermiogenesis: 0 = absent; 1 = present.

*Length: 1, CI: 1, RI: 1*

The presence of microtubules in the implantation fossa during spermiogenesis was recovered as a synapomorphy for the subfamily Smeringopinae.

**Character 21:** Shape of the postcentriolar elongation of the nucleus (in cross-section): 0 = round to oval; 1 = flattened to triangular; 2 = *MiRa: “with a distinct projection” now scored in Character 45*; 3 = flag-shaped.

*Length: 6, CI: 0.17, RI: 0.29*

The postcentriolar elongation of the nucleus appeared to be mostly flattened to triangular in the studied pholcids. This is probably the plesiomorphic condition. A round to oval configuration evolved repeatedly in Modisiminae, Smeringopinae, and Pholcinae.

**Character 22:** Length of the postcentriolar elongation of the nucleus: 0 = <1/2 of the prcN; 1 = similar length as the prcN; 2 = >2 of the prcN; 3 = >5 of the prcN; 4 = < 1/5 of prcN; 5 = absent.

*Length: 8, CI: 0.38, RI: 0.69*

The length of the postcentriolar elongation of the nucleus relative to the precentriolar portion varied among the investigated species. A short peN appears to be synapomorphic for Ninetinae, Smeringopinae and a large clade within Pholcinae (*sensu* Huber *et al*. [7]). Both artemine species included in this study (*Artema* *bunkpurugu*, *Physocyclus* *globosus*) as well as most modisimines and the pholcine *Metagonia* cf. *petropolis* showed a peN length >2 x prcN. Very long postcentriolar elongations of the nucleus (>5x prcN) were only observed in *Aetana* spp. and *Spermophora* *awalai*. The plesiomorphic condition for Pholcidae is unclear.

**Character 23:** Position of the nuclear canal (in the precentriolar part of the nucleus): 0 = within the main part of the nucleus; 1 = within a projection; 2 = within a distinct crest; 3 = central.

*Length: 11, CI: 0.27, RI: 0.62*

The position of the nuclear canal along the prcN is highly variable in Pholcidae but relatively consistent within subfamilies (except Pholcinae). In Ninetinae, Arteminae, and most Smeringopinae (except *Smeringopus* cf. *roeweri*) the nuclear canal is situated in a projection. This is presumably plesiomorphic for Pholcidae. In most Modisiminae (except *Tupigea* *teresopolis* and *Carapoia* spp.) it runs within the main part of the prcN. In Pholcinae, the canal either runs within the main part of the prcN or it has a central position.

**Character 24:** Appearance of the surface of the prcN: 0 = smooth (sometimes with elevated NC; see char. 23); 1 = helical band of nuclear material (independent of NC); 2 = irregularly folded (longitudinal ridges); 3 = threadlike.

*Length: 3, CI: 0.33, RI: 0.75*

A smooth prcN is plesiomorphic for Pholcidae. A helical band of nuclear material on the surface of the precentriolar part of the nucleus only occurs in Pholcinae, possibly with more than one origin.

**Character 25:** Length of the proximal centriole: 0 = approx. as distal centriole; 1 = >2 x as long as distal centriole.

*Length: 3, CI: 0.33, RI: 0*

Most investigated taxa share the plesiomorphic sate of two centrioles of the same length. Only *Smeringopus* cf. *roeweri* and the pholcines *Cantikus* *sabah* and *Pholcus* spp. show an elongated distal centriole.

**Character 26:** Position of proximal centriole: 0 = adjacent to distal centriole; 1 = distant from distal centriole.

*Uninformative*.

**Character 27:** Centriolar adjunct material redefined (see new characters 46, 47, 48). Original numbering of remaining characters stays unaffected*.*

**Character 28:** Axonemal pattern: 0 = 9 + 3; 1 = 9 + 0; 2 = 12 + 0.

*Uninformative*.

**Character 29:** Length of the axoneme (indicated by number of coils in mature spermatozoa): 0 = >2.5 coils; 1 = <2 coils.

*Length: 3, CI: 0.33, RI: 0.50*

Most studied species share the plesiomorphic state (axoneme coiled more than 2.5 times in mature spermatozoa). Exceptions (with fewer than two coils) occur in most Ninetinae (except *Pholcophora* and *Tolteca*) and in the smeringopine *Smeringopus cylindrogaster*.

**Character 30:** Midpiece: 0 = absent; 1 = present.

*Uninformative*.

**Character 31:** Glycogen around the anterior part of the axoneme: 0 = absent; 1 = present.

*Length: 4, CI: 0.25, RI: 0.40*

Glycogen around the anterior part of the axoneme occurs only in the artemine *Physocyclus* *globosus* and the modisimines *Priscula* sp. n. Ecu93, *Tupigea* *teresopolis*, *Chibchea* *salta* and *Psilochorus* *simoni*.

**Character 32:** Clusters of glycogen surrounded by membranes (after coiling process): 0 = present; 1 = absent.

*Uninformative.*

**Character 33:** Membrane stacks in mature spermatozoa: 0 = absent; 1 = present.

*Length: 7, CI: 0.14, RI: 0.14*

Membrane stacks in mature spermatozoa have repeatedly evolved in all subfamilies except Pholcinae, apparently with several reversals.

**Character 34**: Lipid inclusions: 0 = present; 1 = absent.

*Uninformative*.

**Character 35:** Giant body 0=present; 1= absent.

*Uninformative*.

**Character 36:** State of coiling of sperm cell components: 0 = incomplete; 1 = complete; 2 = not coiled (stretched).

*Uninformative*.

**Character 37:** Vesicular area: 0 = absent; 1 = present.

*Length: 6, CI: 0.16, RI: 0.28*

In Pholcidae, a vesicular area seems to have evolved independently in several modisimines: *Priscula* sp. n. Ecu93, *Ciboneya* *antraia*, *Psilochorus* *simoni*, *Otavaloa* cf. *piro*, *Mecolaesthus* sp. n. Ecu60 and *Canaima*? sp. n. Dup118. The absence of a vesicular area seems to be plesiomorphic for Pholcidae.

**Character 38:** Transfer form: 0 = encapsulated; 1 = unsheathed (not encapsulated).

*Autapomorphic*.

Unsheathed synspermia have only been observed in the ninetine *Galapa* *bella*.

**Character 39:** Formation site of the secretion sheath: 0 = deferent ducts; 1 = testes; 2 = ejaculatory duct.

*Autapomorphic*.

Except for the modisimine *Psilochorus simoni*, all studied species form their transfer forms in the deferent ducts.

**Character 40:** Type of transfer form: 0 = coenospermia; 1 = cleistospermia; 2 = synspermia; 3 = rouleaux.

*Length: 3, CI: 0.33, RI: 0.71*

Most studied Ninetinae share the plesiomorphic synspermia, while all representatives of the other subfamilies transfer cleistosperm. The only exception in Ninetinae are *Pholcophora* spp. and *Tolteca* spp., which also transfer cleistosperm.

**Character 41:** Shape of prcN: 0 = tubelike; 1 = helically contorted; 2 = irregularly shaped; 3 = distinct longitudinal ridges.

*Uninformative*.

**Character 42**: Length of AV: 0 = <0.5 of prcN; 1 = 0.5 of prcN; 2 = >0.5 of prcN.

*Length: 8, CI: 0.25, RI: 0*

Most of the investigated species have a short acrosomal vacuole (length of <0.5 x prcN). Exceptions are the artemines *Artema* *bunkpurugu* and *Physocyclus* *globosus*, the modisimine *Modisimus* *elongatus* and the pholcine *Aetana* spp., which have an acrosomal vacuole that is about half of the length of the prcN. Furthermore, the ninetine *Nerudia* spp., the modisimines *Tupigea* *teresopolis* and *Chibchea* *salta* as well as the pholcine *Spermophora* *awalai* possess an acrosomal vacuole that is longer than half the length of the prcN. The plesiomorphic condition is unclear.

**Character 43:** Golgi derivatives (after coiling process): 0 = absent. 1 = present. Golgi derivatives are provided with a double membrane and contain, e.g., secretions or mitochondria.

*Length: 5, CI: 0.20, RI: 0.33*

Golgi derivates have only been observed in the transfer forms of *Nerudia* spp., *Metagonia* cf. *petropolis*, *Micropholcus* *fauroti*, *Cantikus* *sabah*, *Pehrforsskalia* *conopyga* and *Pholcus* spp. The absence of Golgi derivates appears to be plesiomorphic for Pholcidae.

**Character 44:** Length of axoneme in relation to prcN: 0 = Ax > prcN. 1 = Ax < prcN. The length of the axoneme in relation to the nucleus is either precisely measured based on surface reconstructions, or estimated based on cross sections of the axoneme and nucleus visible in 2D TEM images.

*Uninformative*.

**Character 45:** Projection along postcentriolar elongation of nucleus: 0 = absent; 1 = present.

*Length: 11, CI: 0.09, RI: 0.33*

This character is evolutionarily highly unstable. The plesiomorphic state for Pholcidae is unknown.

**Character 46:** Centriolar adjunct material: 0 = absent; 1 = present.

*Length: 8, CI: 0.13, RI: 0.46*

Centriolar adjunct material appears to have evolved independently in the subfamilies Modisiminae and Pholcinae, in both cases with reversals. The absence of centriolar adjunct material appears to be plesiomorphic for Pholcidae.

**Character 47:** Position of centriolar adjunct material: 0 = anterior; 1 = posterior.

*Length: 3, CI: 0.33, RI: 0.33*

Anterior centriolar adjunct material was only observed in Pholcinae. Posterior centriolar adjunct occurs in many Modisiminae, but is also present in the pholcines *Aetana* spp. and *Pehrforsskalia* *conopyga*.

**Character 48:** Anterior centriolar adjunct: 0 = electron dense homogenous; 1 = fibrillar.

*Uninformative* (see below).

The pholcines *Panjange* *camiguin* and *Micropholcus* *fauroti* share electron dense and homogenous anterior centriolar adjunct material. A fibrillar configuration is shared by all studied species of *Pholcus*. It is thus phylogenetically informative, but here considered ‘uniformative’ because *Pholcus* species are pooled.

**Character 49:** Posterior centriolar adjunct material: 0 = electron dense chambered centriolar adjunct around anterior part of the axoneme; 1 = fibrillar chambered centriolar adjunct around anterior part of the axoneme; 2 = collar of electron dense layered (*new term*) lamellae around anterior part of the axoneme; 3 = collar of beaded filamentous centriolar adjunct around anterior part of the axoneme;

4 = collar of spoked lamellae around anterior part of axoneme.

*Length: 3, CI: 0.67, RI: 0.67*

Posterior centriolar adjunct material was observed in different configurations across the studied species. A collar of electron dense and layered lamellae seems to by synapomorphic for a group of modisimines including *Mesabolivar* spp., *Otavaloa* cf. *piro*, *Carapoia* spp. and *Mecolaesthus* sp. n. Ecu60; it evolved independently in the pholcine *Pehrforsskalia* *conopyga*. A collar of electron dense and beaded filaments only occurs in more ‘basal’ Modisiminae, including *Tupigea* *teresopolis*, *Chibchea* *salta*, *Modisimus* *elongatus* and *Ciboneya* *antraia*. A unique configuration among the studied taxa is present in the pholcines *Aetana* spp., which show a collar of spoked lamellae along the anterior part of the axoneme.
